# Acidity and basicity interplay in amide and imide self-association†
†Electronic supplementary information (ESI) available: Experimental and computational details. Protocol for the recording of ^1^H-DOSY and ^1^H-NMR titrations (**A1**, **A2**, **A4–A7**, **I1–I5** and **I8**). Correlations of the computed acidity and basicity with experimental data. Molecular graphs of the monomers and dimers of amides and imides computed with SMD-M06-2x/6-311++G(2d,2p) electron densities. Characterisation of selected HBs in terms of the topological properties of *ρ*(**r**) such as delocalisation index and the Interacting Quantum Atoms energy partition. *XYZ* coordinates and electronic energies of all species addressed in the paper. See DOI: 10.1039/c8sc01020j


**DOI:** 10.1039/c8sc01020j

**Published:** 2018-04-05

**Authors:** Wilmer E. Vallejo Narváez, Eddy I. Jiménez, Eduardo Romero-Montalvo, Arturo Sauza-de la Vega, Beatriz Quiroz-García, Marcos Hernández-Rodríguez, Tomás Rocha-Rinza

**Affiliations:** a Institute of Chemistry , National Autonomous University of Mexico , Ciudad Universitaria , Circuito Exterior, Del. Coyoacán , Mexico City , 04510 , Mexico . Email: marcoshr@unam.mx ; Email: tomasrocharinza@gmail.com

## Abstract

Amides dimerise more strongly than imides despite their lower acidity. Such an unexpected result has been rationalised in terms of the Jorgensen Secondary Interactions Hypothesis (JSIH) that involves the spectator (C=O_S_) and H-bonded (C=O_HB_) carbonyl groups in imides. Notwithstanding the considerable body of experimental and theoretical evidence supporting the JSIH, there are some computational studies which suggest that there might be other relevant intermolecular interactions than those considered in this model. We conjectured that the spectator carbonyl moieties could disrupt the resonance-assisted hydrogen bonds in imide dimers, but our results showed that this was not the case. Intrigued by this phenomenon, we studied the self-association of a set of amides and imides *via*^1^H-NMR, ^1^H-DOSY experiments, DFT calculations, QTAIM topological analyses of the electron density and IQA partitions of the electronic energy. These analyses revealed that there are indeed repulsions of the type O_S_···O_HB_ in accordance with the JSIH but our data also indicate that the C=O_S_ group has an overall attraction with the interacting molecule. Instead, we found correlations between self-association strength and simple Brønsted–Lowry acid/base properties, namely, N–H acidities and C=O basicities. The results in CDCl_3_ and CCl_4_ indicate that imides dimerise less strongly than structurally related amides because of the lower basicity of their carbonyl fragments, a frequently overlooked aspect in the study of H-bonding. Overall, the model proposed herein could provide important insights in diverse areas of supramolecular chemistry such as the study of multiple hydrogen-bonded adducts which involve amide or imide functional groups.

## Abstract 2

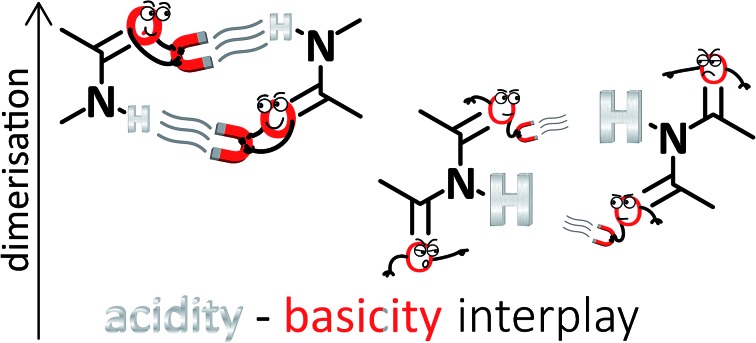
Simple acid–base properties explain the differences in amide and imide dimerisation, and represent an alternative to the secondary interactions hypothesis.

## Introduction

Hydrogen bonds (HBs) in amides and imides are ubiquitous directional forces in nature. HBs in these functional groups are responsible for the secondary interactions, along with other interactions of the tertiary and quaternary structures of proteins.^1^,^2^ Other molecules in which hydrogen bonding is relevant are nucleic acids. The recognition among nitrogenous bases such as uracil and thymine is crucial in the DNA, RNA and other related systems.^3^ Besides HBs, base stacking, steric and electrostatic effects can be important in the stabilisation of nucleic acids.^4^ Additionally, the dimerisation of amides and imides is of interest in several fields such as supramolecular chemistry in which the self-recognition of these functional groups is relevant.^5^,^6^

The formation of amide and imide dimers is frequently studied by NMR or IR spectroscopy to determine self-association constants in solution.^7^,^8^ Rebek and co-workers^9^ examined the intramolecular imide–imide and amide–amide association through NMR measurements. The corresponding results indicate that despite their lower acidity, amides exhibit stronger self-associations than imides. Electronic structure calculations made by Jorgensen and co-workers are consistent with these unexpected experimental observations.^10^ We consider herein a rationalisation of the surprising stronger self-association of amides as compared with imides based on three different criteria.

### (i) Repulsions involving the spectator carbonyls of imides

**Fig. 1:**
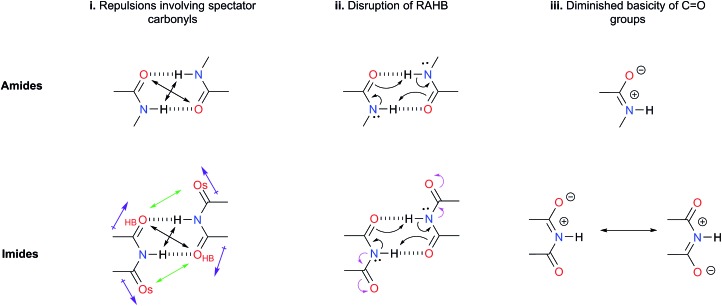
Hypotheses (i)–(iii) considered in this work to explain the larger self-association of amides as compared with that of imides.

The Jorgensen Secondary Interactions Hypothesis (JSIH),^11^ is usually invoked to explain the larger *K*_dimer_ of amides as compared to imides in spite of their lower acidic character.^9^,^10^ The JSIH considers that amides and imides have a similar kind of primary (hydrogen-bonded) interactions, namely O_HB_···H contacts as shown in Fig. 1-i. On the other hand, secondary electrostatic interactions (attractions or repulsions between 2.3 and 3.7 Å)^11^ among atoms in the neighbourhoods of HBs are different in amide and imide dimers. In both cases the molecular clusters present secondary repulsive interactions of the carbonyl oxygens (O_HB_···O_HB_) and hydrogens (H···H) involved in HBs between the monomers (black double arrows in the above-mentioned figure). Nevertheless, the imide dimers present two additional repulsive interactions O_HB_···O_S_ (green arrows), wherein O_S_ denotes the oxygen atom in a spectator carbonyl group. The repulsions O_HB_···O_S_ are also consistent with the notion of two nearly parallel repulsive C=O electric dipoles in the imide dimers (purple arrows). There is a considerable body of theoretical and experimental work which is concordant with the JSIH. For example, crystallographic data of some imides such as maleimide, *trans*-3,4-diphenylsuccinimide, and 1-methyl-hydantoin show that the separation between O_S_ and O_HB_ is very long (>4 Å)^12^ in agreement with the proposed repulsive character of the interaction of these atoms. Additionally, the JSIH has also been successfully used to study tetrapeptide analogues,^13^ stabilisation of base-pairs (A–T and G–C),^14^ and triply hydrogen bonded complexes.^15^

### (ii) Disruption of the resonance-assisted hydrogen bond (RAHB) in imide dimers

As schematised by the black arrows in Fig. 1-ii, the hydrogen bonds in both amide and imide dimers can be regarded as resonance-assisted hydrogen bonds. Electron withdrawing groups adjacent to these RAHBs^16^,^17^ could impair the electron flux across these interactions (magenta arrows shown at the bottom of Fig. 1-ii).

### (iii) Intrinsic acid/base properties of amides and imides

An analysis of the inductive and resonance effects in these compounds suggests not only a higher acidity of the protons in imides but also a smaller basicity of their oxygens, because of the distribution of the negative charge of the zwitterionic structure across two carbonyl groups as illustrated in Fig. 1-iii. The hydrogen bond strength relates to the acid/base properties of the hydrogen donor and the acceptor groups involved in the interaction. Nonetheless, the basicity of the carbonyl is often overlooked when addressing HBs involving this moiety. We conjectured that the acidity of the N–H fragment together with the basicity of the carbonyl group could be responsible for the differences in the self-association of imides and amides.

Given this background, we performed a combined experimental and theoretical study to elucidate the factors governing the self-association of amides and imides. We analysed hypotheses (i)–(iii) through ^1^H-NMR titrations, ^1^H-DOSY experiments, electronic structure calculations as well as quantum chemical topology tools, namely, the Quantum Theory of Atoms in Molecules (QTAIM)^18^ and the Interacting Quantum Atoms (IQA) energy partition.^19^ Our results indicate the existence of repulsions between O_HB_ and O_S_, but these interactions are more than compensated by other intermolecular attractions. Moreover, we found that criterion (iii) is the most suitable to explain the experimental tendencies of the self-association of imides and amides through an interplay of the respective basicity and acidity of the C=O and N–H groups. In other words, a weak HB acceptor carbonyl (as it is the case in many of the examined imides) can significantly weaken the investigated self-association processes despite a strong acidity of the imidic hydrogen and *vice versa*. Our results not only explain the studied phenomenon but also provide a model which could be exploited in diverse areas of supramolecular chemistry, such as the study of multiple hydrogen-bonding complexes which entail the amide and imide functional groups.

## Results and discussion

### Self-association of amides and imides: ^1^H-NMR and ^1^H-DOSY

First, we studied the dimerisation of 2-pyrrolidone (**A1**), an archetype for the study of the self-association of the functional groups concerned in this investigation,7b,^20^ in different deuterated solvents such as chloroform-*d*, acetonitrile-*d*_3_ and DMSO-*d*_6_, and also considered the reported value in CCl_4_ in order to select the most suitable solvent for this research. Another feature of 2-pyrrolidone which makes it particularly suitable for this purpose is the fact that it is liquid at room temperature and thereby its self-association is easier to study in deuterated solvents which cover a broad range of polarity. As expected, *K*_dimer_ diminishes with the polarity of the medium (Table 1), to the point that it is very complicated to reliably determine the self-association constant of **A1** in solvents as polar as DMSO. Additionally, common imides are usually found as crystalline solids and therefore they are rather insoluble in apolar environments. Hence, we decided to carry out the measurements of *K*_dimer_ in chloroform-*d* due to its optimal compromise between the solubility of the investigated systems and the possibility to accurately determine their self-association constants. The attainment of this compromise prevented the analysis of a larger collection of amides and imides than that considered in this paper.

**Table 1:** *K*_dimer_ of 2-pyrrolidone in different solvents at 25 °C

Solvent	*K*_dimer_ (M^–1^)
Carbon tetrachloride	145.0 (ref. 10)
Chloroform-*d*	2.7^*a*^
Acetonitrile-*d*_3_	0.3^*a*^
DMSO-*d*_6_	<0.1

^*a*^Error values below 1% (see Fig. S1 in the ESI).

Next, we employed ^1^H-DOSY experiments to evaluate if imide and amide dimers are indeed the predominant supramolecular aggregates in CDCl_3_. This circumstance is particularly relevant in the case of imides for which it has been suggested that larger clusters might be formed in solution^21^ while polymeric structures can occur in the solid state.^22^ More specifically, we used this technique to determine the hydrodynamic radii (*r*_H_) of the studied amide and imide dimers as reported in Table 2 (the structures of the referred compounds are displayed in Table 3). In addition, we also computed the theoretical hydrodynamic radii from electronic structure calculations (*r*_calc_). As expected, structurally similar compounds have close values for *r*_H_ and *r*_calc_, *e.g.*, the radii of amides **A1**, **A2** and imide **I4** are alike because all of them are unsubstituted five-membered rings. **A4**, **A5** and **I1** are likewise six-membered heterocycles with closely related structures as reflected in their values of *r*_calc_ and *r*_H_. Nevertheless, the cyclic chains in the uracil derivatives **I5** and **I8** and the benzo-derivatives **A6**, **A7** and **I3** introduce discrepancies between *r*_H_ and *r*_calc_. This condition occurs because the spherical particle model employed in the estimation of *r*_H_ does not represent the oblate spheroid character of these dimers. Despite these differences, the experimental *r*_H_ values are always smaller than the computed *r*_calc_ data (apart from **I8** for which *r*_calc_ and *r*_H_ are similar). These results indicate that the investigated amides and imides do not form trimers or larger clusters to an appreciable extent.

**Table 2:** Hydrodynamic radii estimated from ^1^H-DOSY experiments (*r*_H_) and computed with the SMD-M06-2x/6-311G++(2d,2p) approximation (*r*_calc_). The values are reported in angstroms

Compound	*r*_H_ ^*a*^	*r*_calc_
**A1**	3.49	4.04
**A2**	3.57	3.96
**A4**	4.16	4.29
**A5**	4.01	4.33
**A6**	3.77	5.30
**A7**	4.11	5.33
**I1**	4.33	4.46
**I2**	5.52	5.81
**I3**	5.04	6.18
**I4**	3.39	4.03
**I5**	5.12	6.30
**I8**	10.58	9.67

^*a*^TMS as an internal standard reference.^23^

**Table 3:** Amides and imides examined in this work. The dimerisation constants, *K*_dimer_, were measured in CDCl_3_ at 25 °C. The reported p*K*_a_ values in DMSO and H_2_O are show in square brackets and parentheses respectively

Key	Structure	*K*_dimer_ ^*a*^ (M^–1^)	p*K*_a_	Key	Structure	*K*_dimer_ ^*a*^ (M^–1^)	p*K*_a_	Key	Structure	*K*_dimer_ ^*a*^ (M^–1^)	p*K*_a_
**A1**	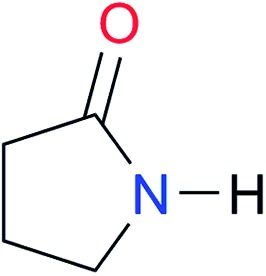	2.7	[24.2]^24^	**I1**	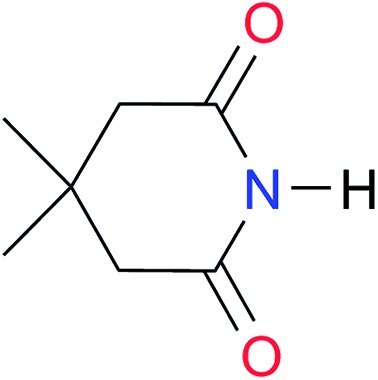	1.4, *K*_corr_: 0.4^*b*^	(11.4)^*c*^ ^,^^25^	**I8**	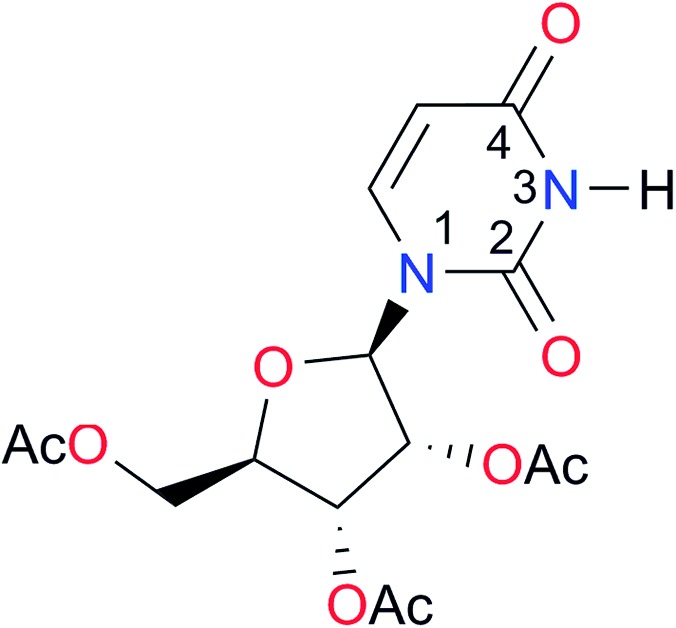	8.6	(9.0)^*d*^ ^,^^26^
**A2**	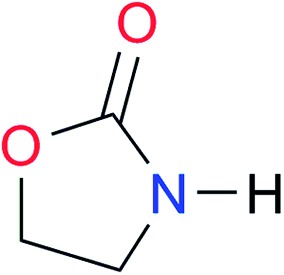	8.3	[20.8]^27^	**I2**	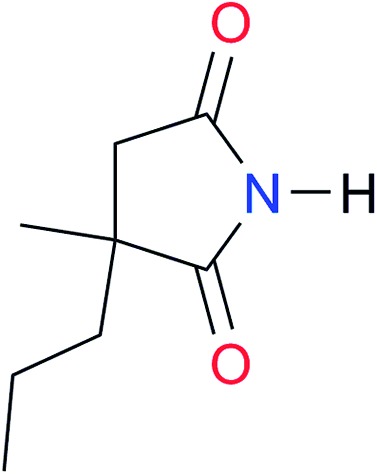	3.3, *K*_corr_: 0.8^*b*^	[14.7]^*c*^ ^,^^33^ (9.6)^*c*^ ^,^^28^	**I9**	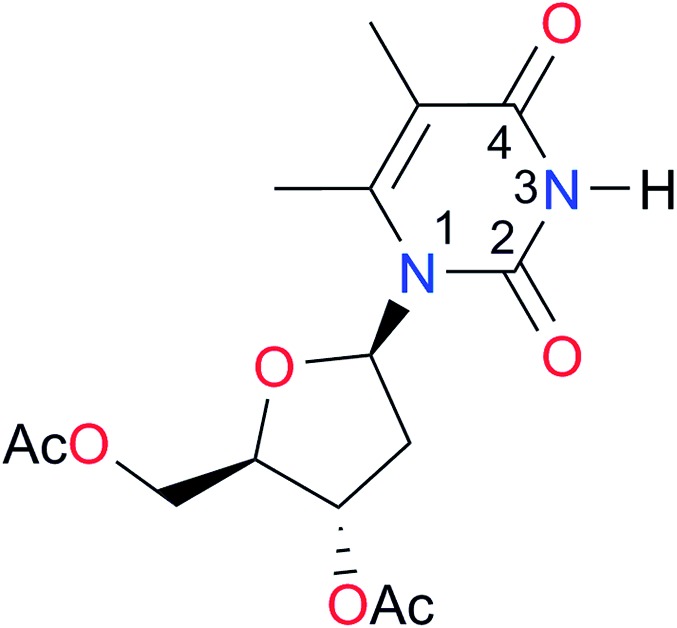	12.7 (ref. 29)	(9.3)^*d*^ ^,^^29^
**A3**	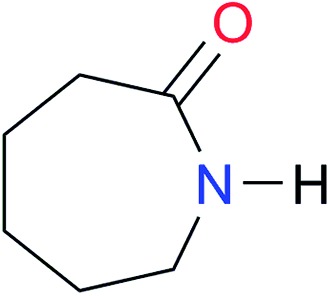	1.0 (ref. 30)		**I3**	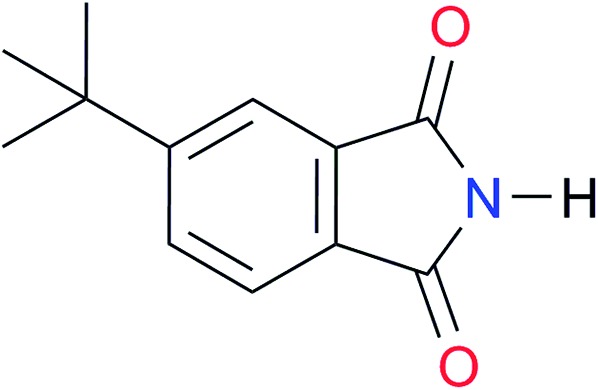	2.1, *K*_corr_: 0.5^*b*^	[13.4]^*c*^ ^,^^31^ (10.2)^*c*^ ^,^^32^	**I10**	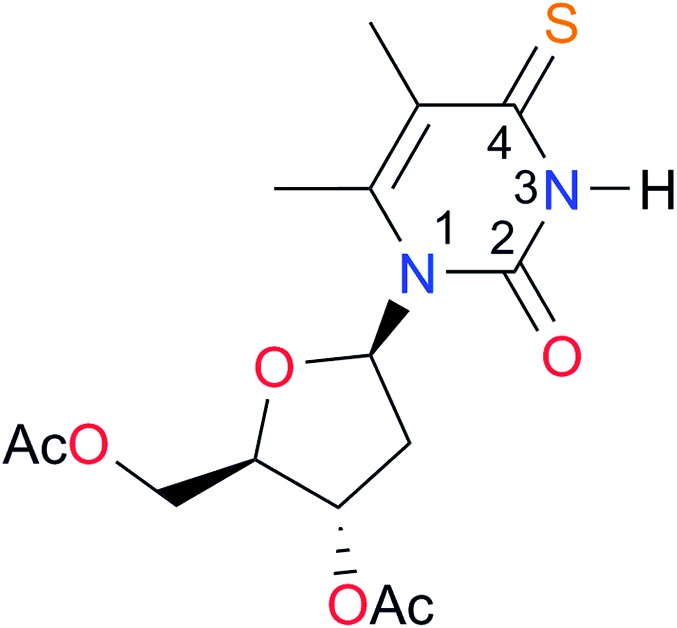	3.1 (ref. 29)	(8.2)^*d*^ ^,^^29^
**A4**	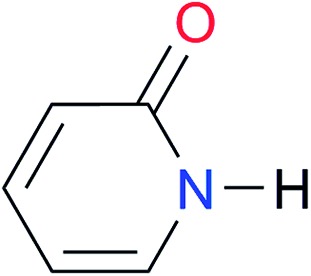	740.0	[17.0]^33^, (11.7)^34^	**I4**	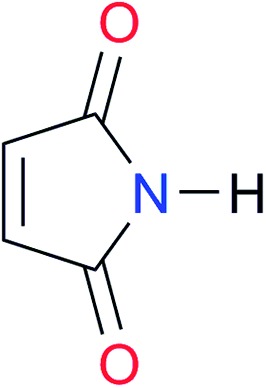	1.2, *K*_corr_: 0.3^*b*^	(4.4)^35^	**I11**	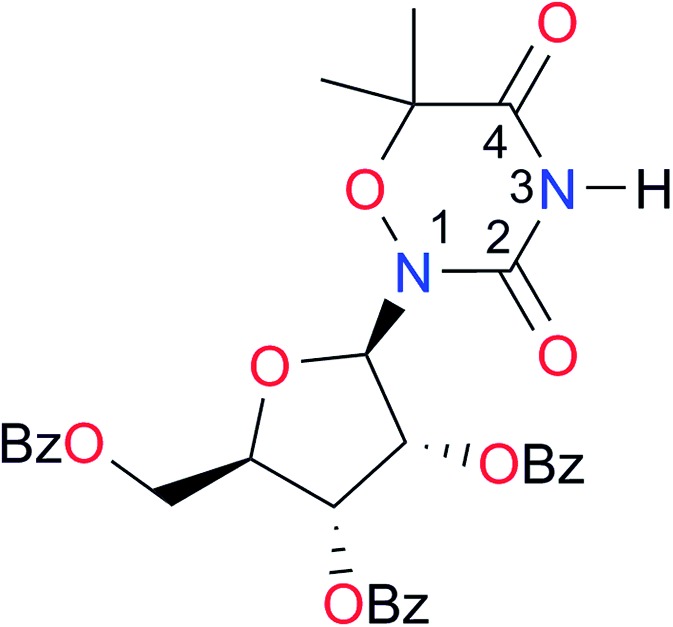	5.3 (ref. 29)	(8.7)^*d*^ ^,^^29^
**A5**	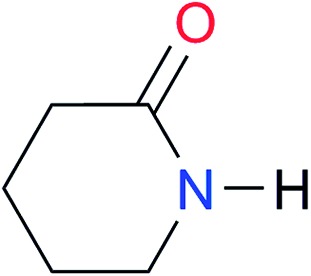	1.8	[26.6]^24^	**I5**	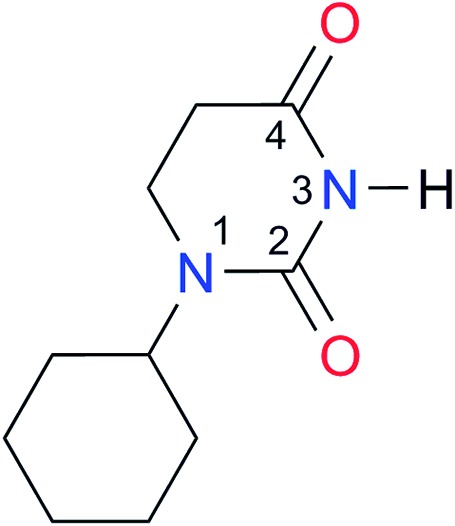	2.6	(9.7)^*e*^ ^,^^36^	**I12**	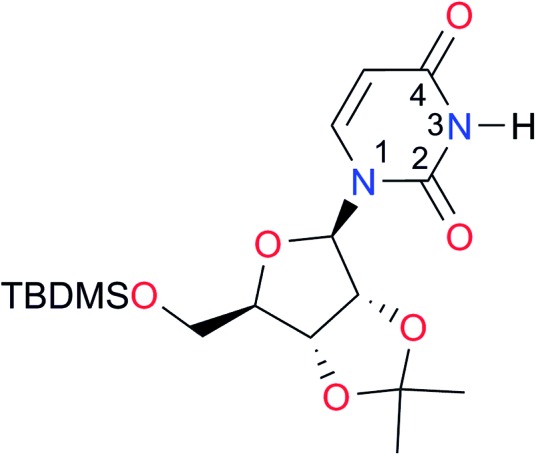	8.0 (ref. 37)	(9.0)^*d*^ ^,^^26^
**A6**	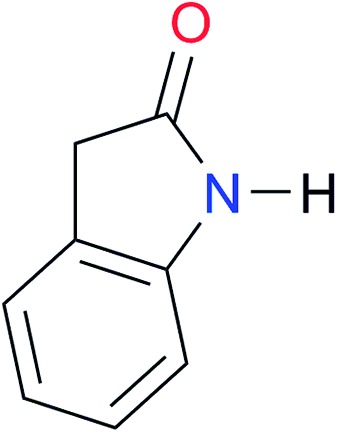	8.0	[18.5]^27^	**I6**	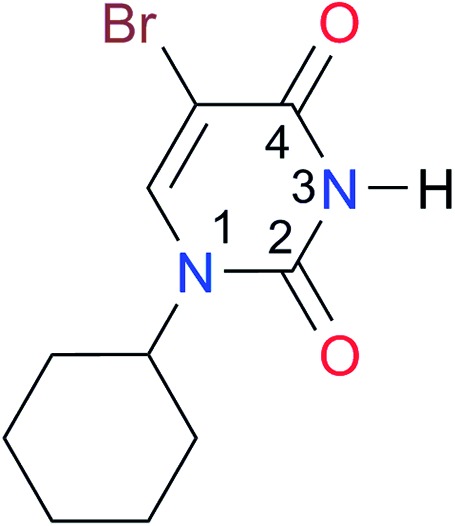	4.1 (ref. 8)		**I13**	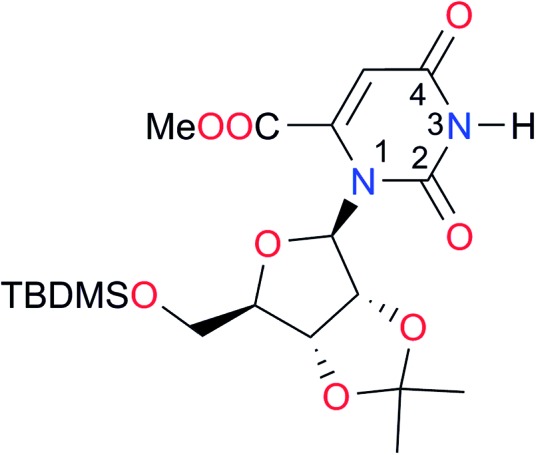	5.0 (ref. 37)	(7.9)^*d*^ ^,^^38^
**A7**	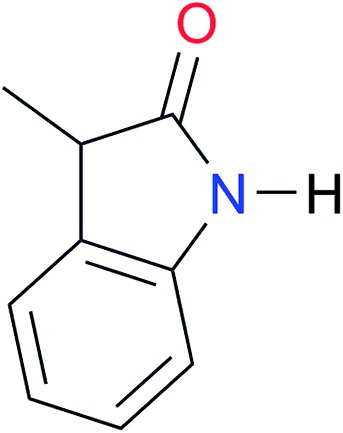	7.6		**I7**	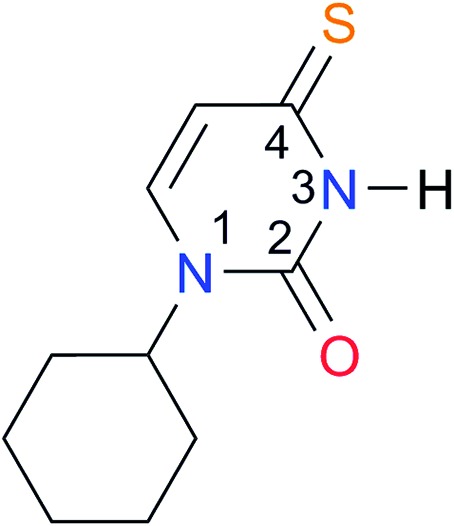	2.7 (ref. 8)	(8.4)^*e*^ ^,^^39^				

^*a*^The values of *K*_dimer_ were determined with errors lower than 2% (see Fig. S2–S13 in the ESI).

^*b*^Statistical factor applied to imides with equivalent (or nearly equivalent) carbonyls and value of the corresponding corrected self-association constant (*K*_corr_).

^*c*^p*K*_a_ of the compound without alkyl substituents.

^*d*^p*K*_a_ of the species without alcohol protecting groups.

^*e*^Data for molecules with a methyl instead of a cyclohexyl substituent.

Once we chose a suitable solvent and verified that the dimeric species are indeed predominantly formed in solution, we consider now the compounds shown in Table 3. This collection of systems contains a good variety of structures and even some previously reported derivatives of nitrogenous bases found in RNA.^3^ In agreement with previous studies,^9^ the self-association of amides is stronger than that of imides for molecules with the same ring size and unsaturation patterns, *e.g.*, **A5***vs.***I1**, **A1***vs.***I2**, **A6***vs.***I3** and **A4***vs.***I6–I13**. These results point out that an increase in acidity does not necessarily lead to a stronger self-association. For example, the decreasing orders of acidity of the structurally related imides (**I7–I11**) are **I7** ≈ **I10** > **I11** > **I8** > **I9**, while those of dimerisation are backwards. We found, however, also exceptions to this behaviour, for instance **A5***vs.***A4** and **I5***vs.***I8** in which acidity and self-association increase in the same direction. Based on the above experimental results, now we proceed to examine the three considered hypotheses concerning the comparison of the self-association between amides and imides.

#### (i) Repulsions involving the spectator carbonyls of imides

**Fig. 2:**
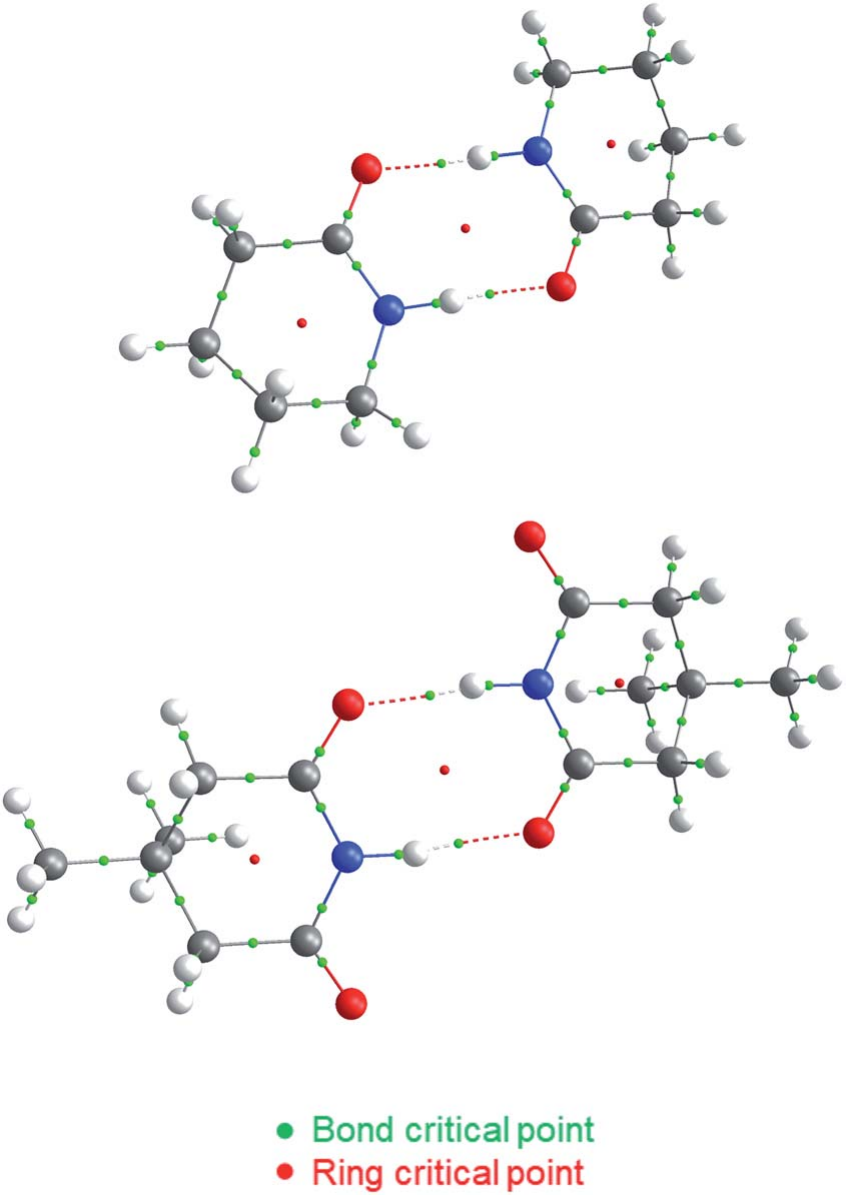
QTAIM molecular graphs for dimers of **A5** (top) and **I1** (bottom). The bond and ring critical points are indicated.

We test here the hypothesis that repulsions involving the spectator carbonyl groups are responsible for the smaller degree of dimerisation of imides as compared with that of amides. For this purpose, we used electron density topology analyses in accordance with the QTAIM theory and the IQA approach. The results shown in Fig. 2 indicate that the intermolecular interactions present in the amide **A5** and the imide **I1** dimers are only HBs. QTAIM shows no repulsion of either kind O_HB_···O_HB_, H···H or O_HB_···O_S_. In particular, we did not detect either attractive or repulsive interactions involving the spectator carbonyl moieties in the analysed imide dimers (Table S1 in the ESI† shows the QTAIM results for the complete set of investigated systems). Nevertheless, QTAIM can be too restrictive in the identification of relevant non-covalent interactions, as it is the case of the weak HB in ethylene glycol.^40^ Therefore, we decided to use the IQA energy partition to determine the attractive or repulsive character of the intermolecular interactions that involve the spectator carbonyls in imide dimers. The left part of Table 4 shows the IQA interaction energies of a spectator oxygen (O13 in the **I1** molecule displayed in green) with the atoms of another **I1** monomer (shown in orange). We note that the interaction between O_HB_ and O_S_ is indeed strongly repulsive (pair O13···O25 in Table 4) because of the electrostatic repulsion between oxygens in accordance with the JSIH. The same atom O_S_ presents however other pairwise interactions which contribute to the attraction between the two monomers (*e.g.*, pairs O13···C14 and O13···C15). After considering all the atoms of the neighbouring molecule, the atom O_S_ has an overall repulsion with the interacting monomer (+10.7 kcal mol^–1^). The nearly parallel C=O_HB_ and C=O_S_ dipoles also present a slight repulsive interaction between them (1.1 kcal mol^–1^ in each case).

**Table 4:** *E*_int_ (IQA) values with the largest magnitudes in homodimer 
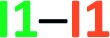
.^*a*^ The data are reported in kcal mol^–1^. The full set of IQA interaction energies can be found in Table S3 in the ESI

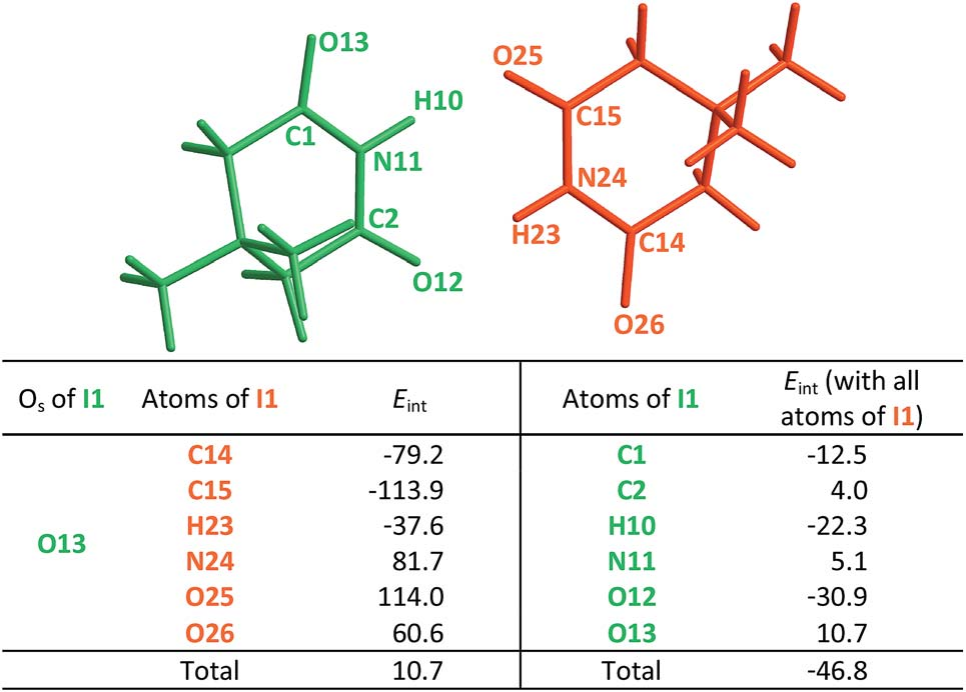

^*a*^The IQA deformation energies of the interacting monomers are 16.4 and 20.0 kcal mol^–1^, therefore the IQA formation energy of the molecular cluster is *E*_form_ = (16.4 + 20.0 – 46.8) kcal mol^–1^ = –10.4 kcal mol^–1^.

The same analysis can also be carried out for every atom in one monomer (say those diplayed in green in Table 4, with the corresponding results shown in the right of the same chart) to obtain the complete IQA interaction energy of the system. The results indicate that not only the atoms considered in the JSIH contribute significantly to the formation of the molecular cluster. In particular, the carbon of the spectator carbonyl group (C1) shows an attraction to the adjacent molecule. The attractive interaction of C1 towards the contiguous monomer more than compensates the repulsion of O13 (–12.5 kcal mol^–1^ + 10.7 kcal mol^–1^ = –1.8 kcal mol^–1^). We computed the same value for the other spectator carbonyl group. This attraction between the spectator carbonyl moiety and the whole interacting molecule evidences the numerous relevant intermolecular atomic pairwise interactions in the system.

The importance of the intermolecular interactions not considered by the JSIH in hydrogen-bonded dimers had already been pointed out by Popelier and Joubert.^41^ Their results were not in accordance with the JSIH in a detailed examination of the relative energetic stability of 27 naturally occurring H-bonded nitrogen base pairs in the gas phase. These researchers considered multipolar expansions of the electrostatic energy (i.e*.*, charge–charge, charge–dipole *etc.* contacts) which included up to terms which depend on *R*^–6^ (*e.g.* dipole–hexadecapole and quadrupole–octupole interactions), *R* being the distance between two multipoles. One of the main conclusions of this study concerns the difficulty to explain the relative stability of H-bonded clusters by only considering a subset of atomic pairs located in the boundaries between the interacting monomers as opposed to taking into account the whole set of intermolecular pairs of atoms in the system.

#### (ii) Disruption of the resonance-assisted hydrogen bonds in imide dimers

**Fig. 3:**
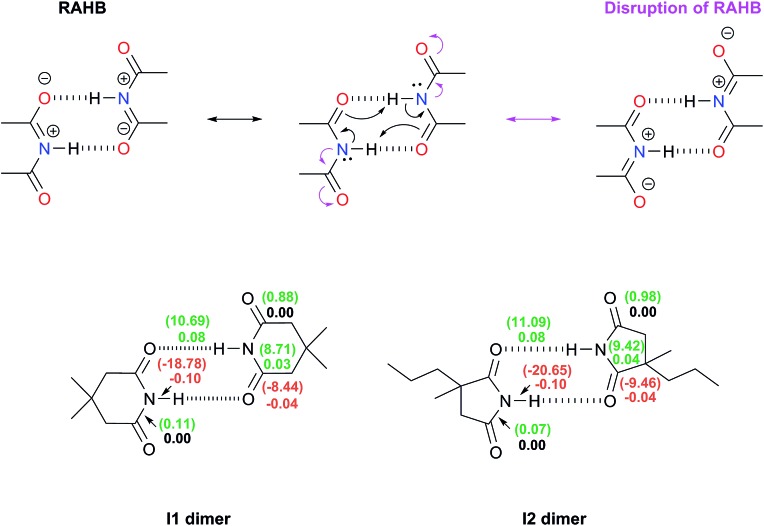
Top: representation of the RAHB and its potential disruption (in magenta) within imide dimers by virtue of the electron-withdrawing nature of their spectator carbonyl groups. Bottom: changes in the electron delocalisation indices around the RAHBs as a consequence of the dimerisation of **I1** and **I2**. The positive values of ΔDI = DI (in dimer) – DI (in monomer) are written in green while those which are negative are displayed in red. The corresponding change of the IQA exchange–correlation component, Δ|*V*_XC_|/kcal mol^–1^, between two atoms is indicated in parentheses.

QTAIM analyses allowed us to consider the potential disruption of the resonance-assisted hydrogen bonds in imide dimers by virtue of their spectator carbonyl groups (top of Fig. 3). The eventual hindrance of the RAHB in the imide adducts would be accompanied by changes in the QTAIM Delocalisation Indices (DIs)^42^ and the IQA exchange–correlation component of the C–N and C=O bonds as schematised in Fig. 1-ii. The DIs, which decrease because of self-association, describe interactions whose covalent character diminishes because of the intermolecular HBs. Conversely, a positive value for ΔDI evidences a chemical bond with an increased covalent character following the formation of the dimer. The same trend analysis can be applied to the change in the magnitude of the IQA exchange–correlation contribution Δ|*V*_XC_| of these bonds. The results shown in the bottom of Fig. 3 indicate that there is no such disruption of the RAHB in imide dimers due to these spectator carbonyl groups. The lengths (Table S10 in the ESI†) along with the Δ|*V*_XC_| values and the ΔDIs for the C–N and C=Os bonds are barely affected. Moreover, in most cases the ΔDI of the spectator C=O and the absolute value of *V*_XC_ increase slightly because of the formation of the RAHB in imide dimers as opposed to the flux of electrons suggested at the top of Fig. 3.

Additionally, the changes in the DIs and distances of the bonds involved in this RAHB are substantially larger than those of the neighbouring C–N and C=Os interactions. These data point out that the disruption of the RAHB in imides is not the factor which explains the larger self-association of amides with respect to imides, a condition consistent with the observation that electron delocalisation is not the most stabilizing effect in resonance-assisted hydrogen bonds.^44^

#### (iii) Intrinsic acid/base properties of amides and imides

**Fig. 4:**
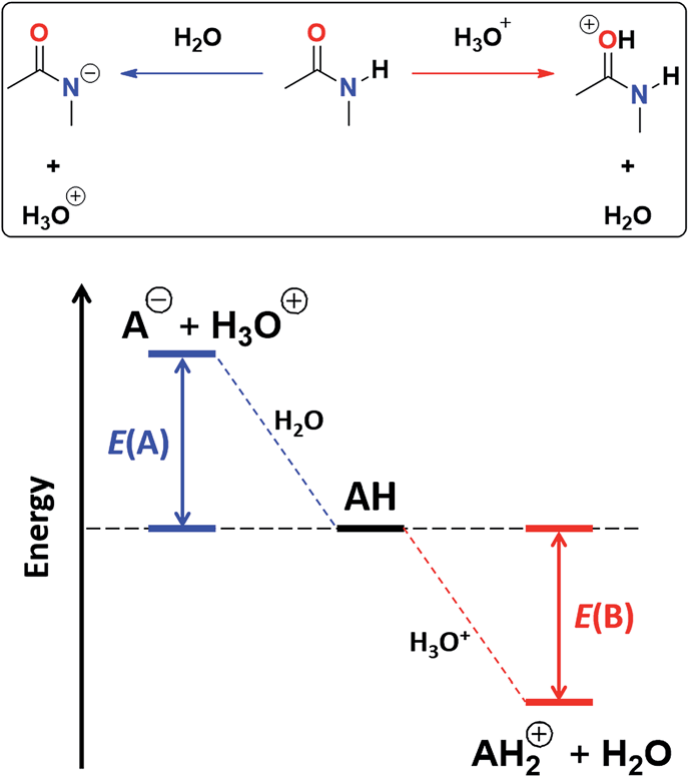
Combined acidity and basicity model of the monomeric species to address the homocoupling of amides and imides. |*E*(A)| and |*E*(B)| are the respective magnitudes of the energies associated with the deprotonation and protonation reactions of the N–H and C=O fragments.

We test now the hypothesis that the differences for self-association between amides and imides are governed by the acidity of the amidic or imidic hydrogen and the basicity of the acceptor carbonyl group. For this purpose, we considered a model which is based on the energies associated with the N–H deprotonation, *E*(A), and C=O protonation, *E*(B), as shown in Fig. 4.^45^ The acidity of the N–H proton is reduced with the magnitude of |*E*(A)| while a high value of |*E*(B)| indicates a strong basicity of the oxygen in the carbonyl group. The deprotonation energies for a wide variety of species which include imides, amides, (thio)ureas,^46^ squaramides^47^ and carboxylic acids^48^ correlate very well with the corresponding experimental p*K*_a_ values, ditto for |*E*(B)| and p*K*_BH+_, which indicates the p*K*_a_ of the conjugate acid of the species under consideration (see Tables S7, S8 and Fig. S29, S30 in the ESI† for the data of the complete set of compounds). Subsequently, we calculated |*E*(A)| and |*E*(B)| for compounds **A1–A7** and **I1–I13** (Table 5).

**Table 5:** ln *K*_dimer_ of the compounds shown in Table 3. The |*E*(B)| and |*E*(A)| values were computed with the SMD-(CHCl_3_)-M06-2x/6-311++G(2d,2p) approximation

Compound	|*E*(A)| (kcal mol^–1^)	|*E*(B)| (kcal mol^–1^)	ln *K*_dimer_
**A1**	90.6	27.0	0.97
**A2**	84.5	17.6	2.11
**A3**	95.4	28.0	0.01
**A4**	78.1	28.8	6.61
**A5**	94.4	28.6	0.59
**A6**	79.7	19.6	2.07
**A7**	79.9	19.8	2.03
**I1**	80.3	13.9	–1.08^*b*^
**I2**	75.4	12.9^*a*^	–0.18^*b*^
**I3**	73.8	10.8^*a*^	–0.63^*b*^
**I4**	73.6	7.7	–1.21^*b*^
**I5**	82.5	20.8^*a*^	0.96
**I6**	74.0	15.5	1.41
**I7**	74.9	13.7	0.99
**I8**	74.9	18.1^*a*^	2.15
**I9**	77.8	22.8^*a*^	2.54
**I10**	73.9	14.3^*a*^	1.13
**I11**	72.4^*c*^	11.9^*c*^	1.67
**I12**	75.5	19.5^*a*^	2.08
**I13**	71.7	15.3	1.61

^*a*^|*E*(B)| value of the most basic oxygen atom within the molecule.^43^ (*e.g.* in **I5**, |*E*(B)| for the other carbonyl group equals 16.1 kcal mol^–1^).

^*b*^ln *K*_dimer_ after the consideration of the statistical factor.

^*c*^Data for the compound without methyl groups.

**Fig. 5:**
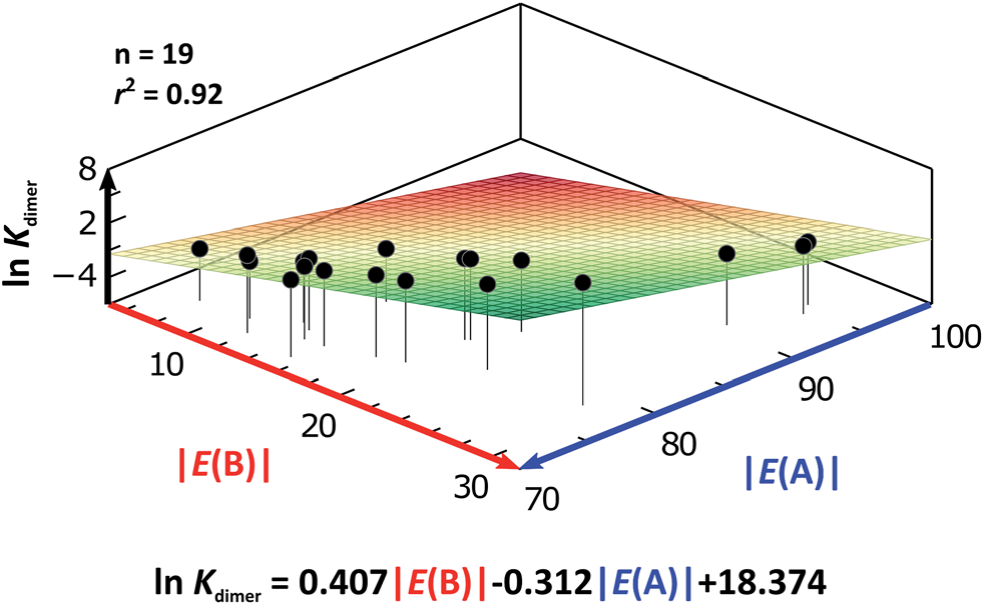
ln *K*_dimer_ as a function of |*E*(A)| and |*E*(B)| (given in kcal mol^–1^) for the compounds shown in Table 3 and the first-degree model adjusted for the distribution of points.

**Fig. 6:**
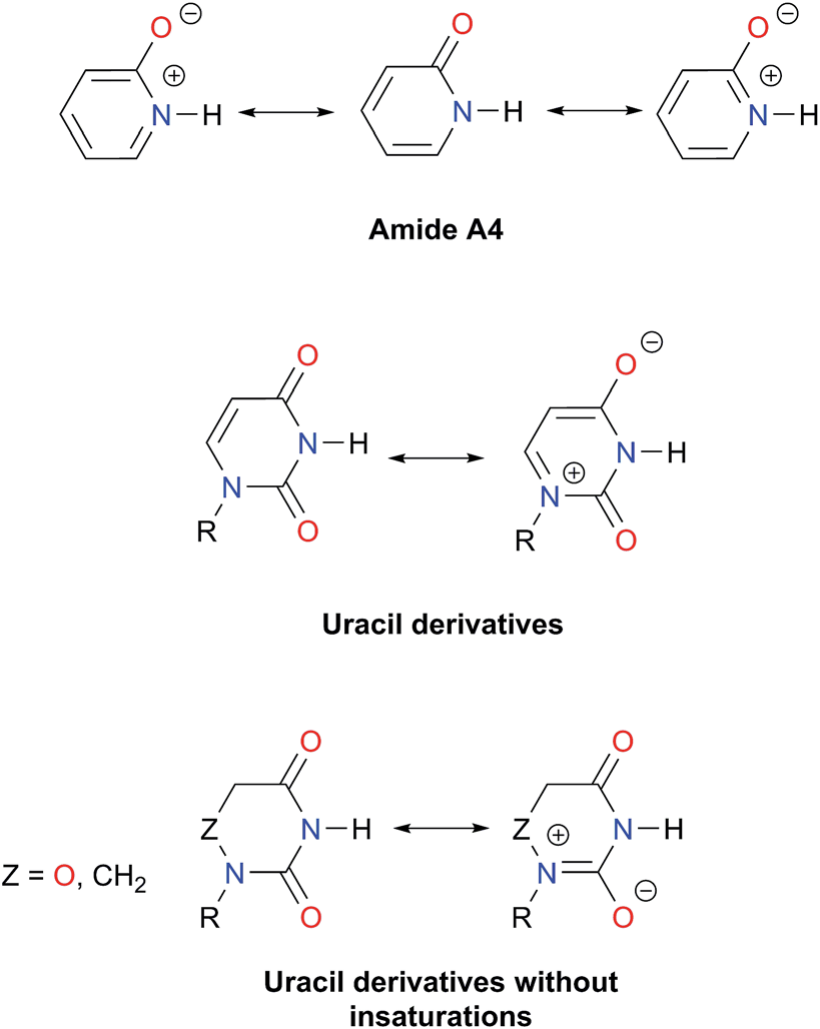
Resonance structures of amide **A4**, uracil derivatives and their saturated analogues.

As expected, imides are more acidic and less basic than amides as suggested by the resonance structures in Fig. 1-iii. The change in Brønsted–Lowry acidity or basicity can substantially modify the self-association constants of hydrogen-bonded systems.^21^,^52^ We found a good correlation of ln *K*_dimer_ as a function of |*E*(A)| and |*E*(B)| whose distribution of points adjusts to a first-degree polynomial model. The species **A2** was not contemplated in the correlation because it is a cyclic carbamate that can form bifurcated hydrogen bonds with CHCl_3_ as indicated by DFT geometry optimisations and schematised in Fig. S31 in the ESI.† We conjectured that this feature can substantially affect the self-association of **A2** in comparison with the rest of the studied compounds. Indeed, the exclusion of **A2** improved the value of *r*^2^ in Fig. 5 considerably. We note that the coefficients multiplying |*E*(B)| and |*E*(A)| (*C*_|*E*(B)|_ and *C*_|*E*(A)|_) are positive and negative respectively. These conditions support the model that self-association increases with the acidity of the N–H moiety and the basicity of the C=O group. Besides, |*C*_|*E*(B)|_| > |*C*_|*E*(A)|_|, and hence the dimerisation processes of the examined compounds are more sensitive to changes in the basicity of the carbonyl group than to modifications of the acidity of the amidic or imidic hydrogen. In general, this model points out that a high acidity or basicity by itself does not ensure a large association constant since there must be a balance between these properties to observe a substantial value of *K*_dimer_. In other words, very poor acceptor or donor features of a system can substantially hinder its self-association process as it is the case for imides and basic amides **A3** and **A5** respectively. This analysis indicates that the low basicity of the carbonyl groups in imides allows us to explain why these compounds dimerise less strongly than amides notwithstanding their larger acidic character. The model in Fig. 5 allows us to interpret a series of experimental observations in CDCl_3_. For example, the lowest value of the dimerisation constant corresponds to maleimide **I4**, a compound with high acidity but the one with the smallest basicity among the analysed systems. On the other hand, **A4** undergoes the strongest self-association among the molecules of interest. It has the highest basicity and an acidic character similar to the examined imides. These results point out that the occurrence of an aromatic sextet (top of Fig. 6) might favor zwitterionic structures that lead to a high self-association constant.^49^ The relevance of the basicity of the C=O fragments in the self-association of amides and imides is also observed for the uracil analogues **I6–I13**. These imides are more basic than other compounds with the same functional group and hence dimerise more strongly. Furthermore, the consideration of the basicity of the C=O groups is also useful to rationalise which fragments are involved in the dimerisation. For example, it is well known that uracil derivatives (**I6**, **I8**, **I9**, **I12** and **I13**) form hydrogen bonds with the oxygen at C-4 rather than the one at C-2.^29^ This finding is consistent with our results which indicate that the carbonyl at position four is more basic than its counterpart at position two. The larger basicity of the oxygen bonded to C-4 is associated with the condition that the corresponding carbonyl is conjugated with N-1 *via* a C-5, C-6 double bond. When this is no longer the case, *e.g.***I5** and **I11**, the most basic carbonyl is the one at C-2 as illustrated in the middle and bottom of Fig. 6 (uracil derivatives with and without unsaturations). These observations are supported by the model proposed herein, 2D-NOE analyses^29^ and proton affinity calculations for **I5** (Table 5).

**Fig. 7:**
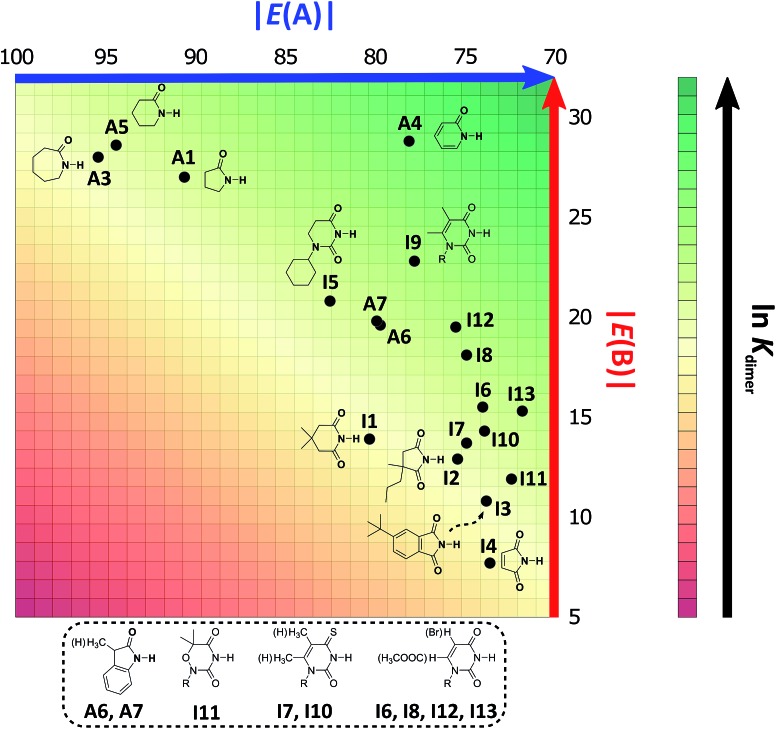
2D graphical representation of the association strength of the investigated set of compounds in CDCl_3_ as a function of acidity and basicity of the N–H and C=O groups respectively. The |*E*(A)| and |*E*(B)| values are displayed in kcal mol^–1^. We indicate the structure and location of each species in the acid/base plane.

Following the same arguments concerning the relative importance of basicity and acidity of the examined compounds, we can explain why among the studied five-membered heterocycles, amide **A1** associates more strongly than imides **I2** and **I4**. Six-membered rings also follow this trend, as observed when comparing **A4***vs.***I5–I13**. The same occurs with derivatives of benzene **A6**, **A7***vs.***I3** as shown in Fig. 7. On the other hand, when two species have a comparable acceptor capacity, the factor which establishes the stronger self-association is the N–H acidity. In this way, we can explain the differences in self-association between compounds of the same family, such as amides **A3**, **A5** and **A1** or imides **I2***vs.***I1**. Another appealing feature of this model is that it explains the unexpected result that the uracil-containing species dimerise more strongly than amides **A5** or **A3**. Given that all these uracil derivatives have similar acidities, the dimerisation strength is, in this case, based on the variation of the C=O basicities. In addition, there is a good correlation between the experimental values of ln *K*_dimer_ with those predicted in the model of Fig. 5 as shown in Fig. S32 in the ESI.† Thus, the consideration of the proton acceptor and donor capacities described in this research is a reasonable alternative approach to interpret the differences in the self-association of amides and imides.

### Self-association in CCl_4_

**Fig. 8:**
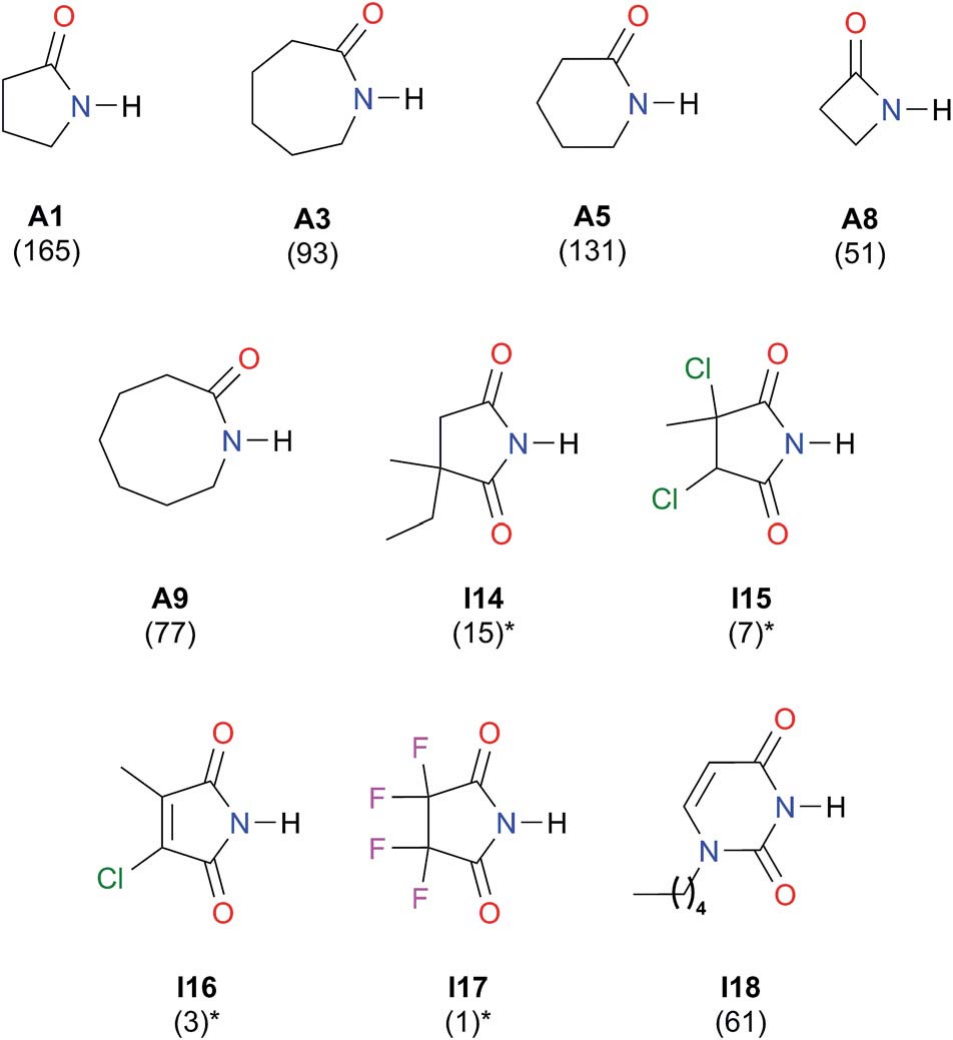
Self-association constants (M^–1^) reported in CCl_4_ by IR spectroscopy.^21^,^50^ *The values were corrected by a statistical factor of four.

**Fig. 9:**
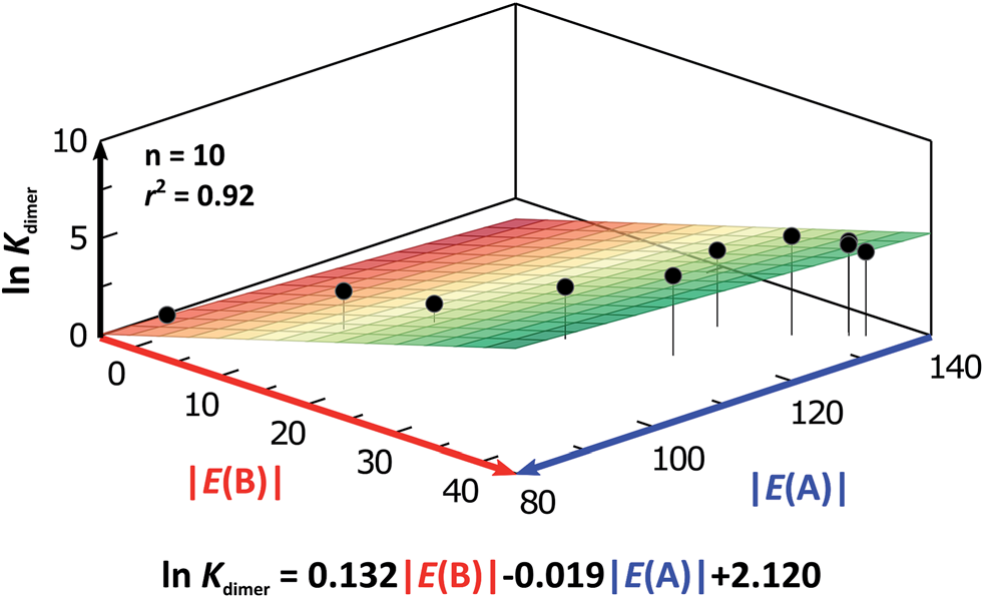
ln *K*_dimer_ as a function of |*E*(A)| and |*E*(B)| (given in kcal mol^–1^) of the molecules shown in Fig. 8 and studied in CCl_4_. The resulting first-degree model adjusted for the distribution of points is reported as well. The corresponding data are reported in Table S9 in the ESI.†

We considered amide and imide dimerisation constants reported in CCl_4_ as well.^21^,^50^ As discussed above, the apolar nature of carbon tetrachloride results in larger dimerisation constants than those obtained in CDCl_3_ (ref. 51) (Fig. 8). The set of examined compounds comprises a variety of systems with electron donor and acceptor fragments and hence it allows us to consider the effect of these functional groups on ln *K*_dimer_. Similar to the previous discussion of the results in chloroform, there is a correlation between (i) ln *K*_dimer_ and (ii) the basicity of the carbonyl group together with the acidity of the N–H moiety as shown in Fig. 9. The models of Fig. 5 and 9 are qualitatively similar. In particular, *C*_|*E*(B)|_ > 0, *C*_|*E*(A)|_ < 0 and |*C*_|*E*(B)|_| > |*C*_|*E*(A)|_|. The last-mentioned condition indicates once again that the dimerisation constant of amides and imides is more sensitive to the basicity of the carbonyl group than to the acidity of the N–H moiety, hence the larger association of amides in comparison to imides.

### Analysis of heterodimers

**Fig. 10:**
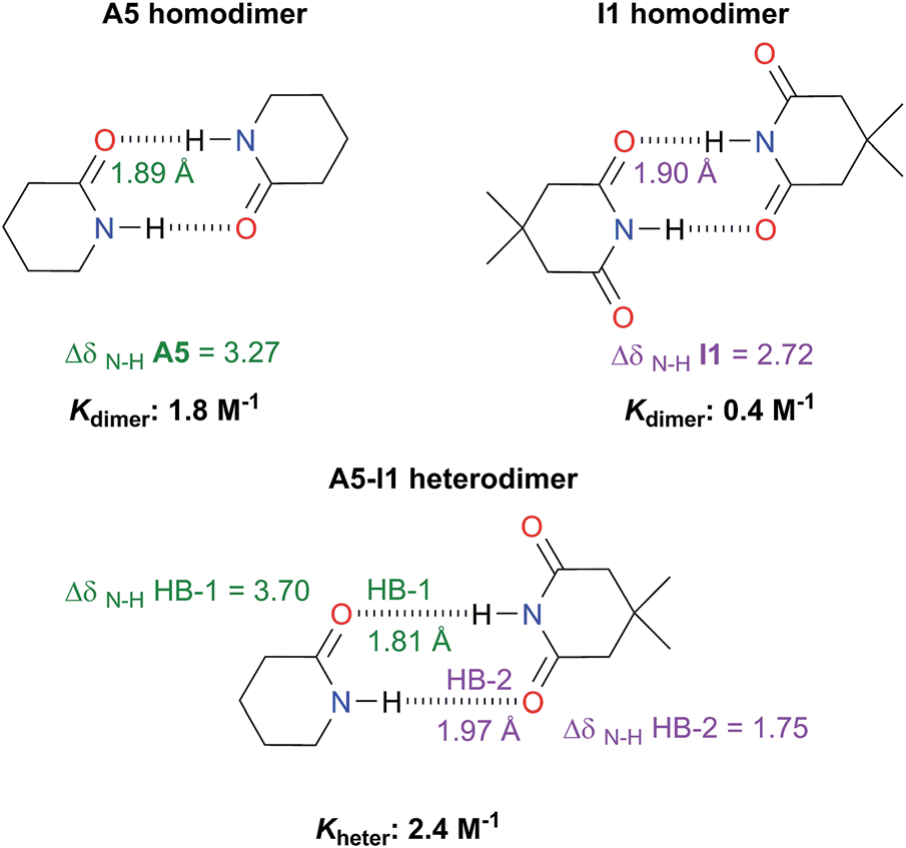
Association constants (considering the statistical factor correction) and changes in chemical shift Δ*δ*_N–H_ (in ppm) of the imidic and amidic protons within the **A5**, **I1**, hetero- and homodimers at infinite dilution (complete information in Fig. S14 in the ESI†). We also show the hydrogen bond lengths calculated at the SMD-(CHCl_3_)-M06-2x/6-311++G(2d,2p) approximation.

The examination of heterodimers gives further insights about the dimerisation of amides and imides. The determination of *K*_heter_ indicates that hetero-association between **A5** and **I1** is stronger than in each homodimer as illustrated in Fig. 10. To rationalise this observation, we consider the relative strength of the individual HBs involved in the heterodimerisation and compared them with those in the homodimers. First, we took into account ^1^H-NMR spectroscopy results. The change in the N–H chemical shift due to complexation, Δ*δ*_N–H_, is reported to increase with the strength of the association.^52^,^53^ The change in chemical shift of the imidic hydrogen within the **A5–I1** adduct (HB-1 in Fig. 10) is larger than that in the **A5** homodimer. Furthermore, the value of Δ*δ*_N–H_ in the amidic hydrogen (HB-2 in the same figure) involved in the heterodimer is smaller than the corresponding value for the HB in the imide homodimer. Fig. S15† shows similar results obtained for **A1** and **I2** heterodimers. The measurement of *K*_heter_ was performed with a constant total amount of amide **A1** while imide **I2** was added to the system. The value of *δ*_N–H_ diminished through the titration, a condition indicative of a weakening of the HB entailing the amidic hydrogen. In the opposite experiment (having a constant total amount of **I2** and titrating with **A1**) the value of *δ*_N–H_ for the imidic proton increased more than in the corresponding homodimers, evidencing the strengthening of the hydrogen bond of the heterodimer which involves this proton. The last-mentioned effect is also observed in the **A5–I1** molecular cluster.

Concerning the **A5–I1** and **A1–I2** heterodimers, the strongest HB-1 is formed between the most acidic proton (the one in the imide) and the most basic carbonyl (found within the amide). On the other hand, the weakest HB takes place in the interaction of the less acidic proton, *i.e.* N–H of amides **A5** and the less basic carbonyl (the one in the imide). The strengths of the HBs in the amide and imide homodimers are between these two extremes. These results along with those of DFT geometry optimisations of the **A5–I1** heterodimer show the following trend of descending HB strength:

HB-1 > HB in **A5** homodimer > HB in **I1** homodimer > HB-2

This order is similar to that found by Jorgensen^10^ and Leszczynski^54^ who studied computationally the hetero- and homodimers of 2-pyrrolidone and succinimide. According to the JSIH, we should expect the hydrogen bond lengths within the heterodimer to be located somewhere between those of the homodimers. Moreover, the shortest hydrogen bond HB-1 is observed next to a presumed electrostatic or dipole–dipole repulsion involving the spectator carbonyl of **A5** as opposed to HB-2 which does not present this effect. Despite the aforementioned agreement of the JSIH with structural data, this hypothesis is not completely consistent with the H-bond strength and distance patterns found in the heterodimers. In contrast, the consideration of the acidity and the basicity of the N–H and C=O fragments explains the observed association constants, hydrogen bond distances and chemical shift patterns in these systems. The QTAIM and IQA analysis of the intermolecular interactions within the heterodimers **A5–I1** (Table 6) and **A1–I2** (Tables S5 and S6 in the ESI†) is consistent with the previous discussion. The oxygen of the spectator C=O in the imide presents a strong repulsion with the oxygen of the neighbouring carbonyl involved in the HB and with the rest of the molecules of the amide. These repulsions are even stronger than in the case of the **I1** homodimer. Still, the hydrogen bond closest to the O_S_ atom in the **A5–I1** heterodimer is the strongest of the system. The relative strength of these HBs is revealed by their lengths as previously discussed and other indices used in the study of hydrogen bonds which include (i) Espinosa's empirical formula^55^ (Fig. S28 in the ESI†) and (ii) the IQA intermolecular attractions with the interacting molecule (H10 along with O32 on one hand and H31 together with O12 on the other) as reported in the middle and right of Table 6. Despite the repulsion of the oxygen in the spectator carbonyl, this C=O group has an overall attractive interaction with the neighbouring imide or amide in a similar fashion to the corresponding homodimers. These IQA homo- and heterodimer results point out that the O_HB_···O_S_ repulsions or those between the corresponding dipole carbonyls are not the decisive factor for the energetics of the amide and imide dimerisation.

**Table 6:** *E*_int_ (IQA) values with the largest magnitudes within the 
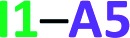
 heterodimer. The data are reported in kcal mol^–1^. The full set of IQA interaction energies can be found in Table S4 in the ESI^*a*^

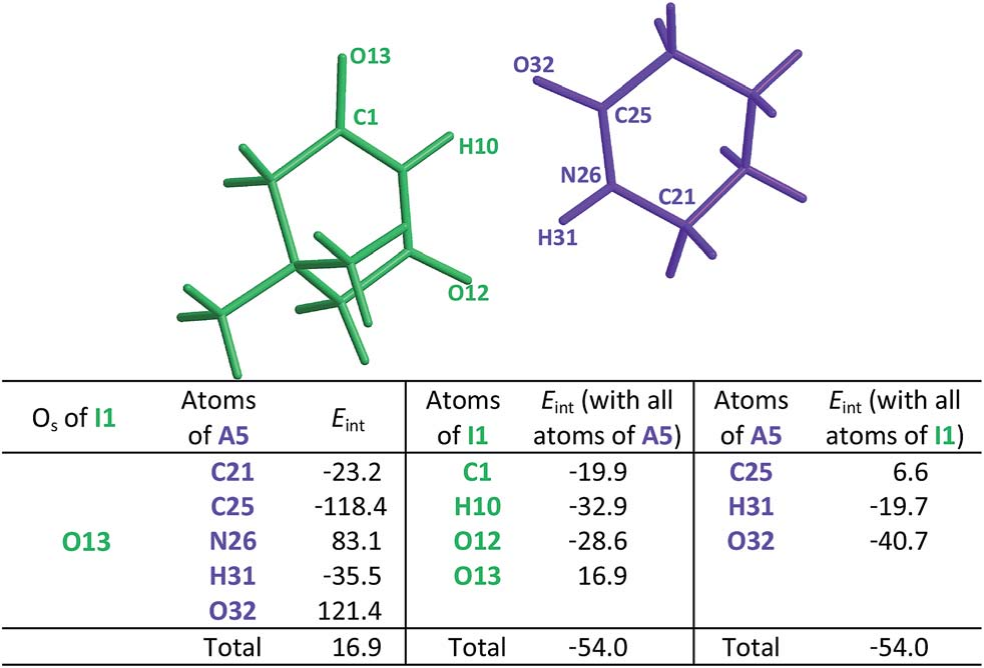

^*a*^The IQA deformation energies of the amide and imide are 20.9 and 21.7 kcal mol^–1^, so that the IQA formation energy of the molecular cluster is *E*_form_ = (20.9 + 21.7 – 54.0) = –11.4 kcal mol^–1^.

### Significance in the field of molecular recognition

Our investigation provides an alternative model to the JSIH to rationalise experimental observations concerning the homo- and heterodimerisation of amides and imides, for example, the hydrogen bond distance pattern in amide/imide heterodimers and the relative magnitude of *K*_dimer_ for these systems. The interplay of acidity and basicity allows us to explain other baffling results *e.g.* the fact that 2-ethyl-2-methylsuccinimide self-associates much more strongly (by a factor of 15) than tetrafluorosuccinimide.^21^ Based on the common consideration of the N–H acidity alone, it is expected that the last-mentioned compound would have a larger *K*_dimer_. Likewise and according to the JSIH, the fluorinated compound should dimerise to a larger extent because the charges in the carbonyl oxygens are less negative than those in the alkylated succinimide. The alternative explanation that we offer is that the fluorine atoms reduce substantially the basicity of the carbonyls, thereby impairing the self-association of this compound.

Additionally, our results can be useful in understanding why amides are much more used in many technologies such as crystal engineering, development of materials and pharmaceuticals than imides.^56^ The analysis presented herein indicates that self-association of these functional groups is more sensitive to the basicity of the C=O moiety than it is to the acidity of the N–H group. Hence, the modification of the basicity of this carbonyl represents a good opportunity in the modulation of the strength of the non-covalent interactions established by these groups. For example, the change of a carbonyl in a crucial amide for a more basic imino fragment in vancomycin enhances the association properties with the cell wall of bacteria immune to this antibiotic.^57^ This increase of association restores antimicrobial activity and represents a strategy to address vancomycin-resistant infections.

Finally, the results of our investigation suggest a different approach for the analysis of the stability of multiple hydrogen-bonded systems such as uracil–diamino purine, cytosine–guanine and ADA–DAD systems.^58^,^59^ Generally, these systems are examined by considering only the acidity and basicity of the intermediate hydrogen bond and the JSIH. It would be nevertheless desirable to examine the Brønsted–Lowry acid/base properties for every HB in the system which could lead to valuable insights about the molecular recognition of hydrogen-bonded homo- and heterodimers.

## Experimental

General experimental and computational details are given in the ESI.†

## Conclusions

We investigated the reasons underlying the stronger self-association of amides as compared to imides. Our results indicate that the spectator carbonyl presents indeed repulsions with the oxygens involved in the HB but these repulsive interactions are not the key factor in the energetics of the H-bonded systems examined herein. This statement is based on the relevance of the pairwise interactions which are not accounted for by the JSIH and the experimental and theoretical examination of amide–imide homo- and heterodimers. Our results also reveal that the spectator carbonyl groups in imides do not interfere with the resonance-assisted hydrogen bonds in the dimers of these species. Then, we consider the Brønsted–Lowry acid/base properties of the HB donors and acceptors involved in the interaction within these molecular aggregates. The examination of the basicity of the C=O group, the acidity of the N–H moiety and *K*_dimer_ resulted in a first-degree model suggesting that the proton acceptor capacity of the carbonyl group is more important than the acidity of the amidic or imidic hydrogens for the self-association of the investigated compounds. This model also indicates that there must be a balance between the respective acidity and basicity of the N–H and C=O fragments to observe a substantial self-association of these systems. Particularly, amides exhibit larger self-association constants because of their higher basicities in comparison with imides. Similar conclusions were drawn from systems investigated in CCl_4_. Our results also explain the hydrogen bond distance patterns found in amide and imide homo- and heterodimers. The acidity/basicity balance in the dimerisation of amides and imides is an alternative approach to the JSIH in order to explain this phenomenon. Overall, we expect that the application of the insights presented herein will prove valuable to understand and modulate hydrogen bonds between the investigated functional groups which are present in a wide variety of supramolecular systems throughout biology and chemistry.

## Conflicts of interest

There are no conflicts to declare.

## Supplementary Material

Supplementary information
