# Supplementary material for: Acidity and basicity interplay in amide and imide self-association
Source: Chem Sci. 2018 Apr 5;9(19):4402–13. doi: 10.1039/c8sc01020j (PMC5956980; doi:10.1039/c8sc01020j)
Supplement: Supplementary file 1 [file SC-009-C8SC01020J-s001.pdf]

Electronic Supplementary Material (ESI) for Chemical Science.
This journal is © The Royal Society of Chemistry 2018

Supporting Information

Acidity and basicity interplay in amide and imide self-association

Wilmer E. Vallejo Narváez,[a] Eddy I. Jiménez,[a] Eduardo Romero-Montalvo,[ab] Arturo Sauza-de la Vega,[a] Beatriz Quiroz-García,[a] Marcos Hernández-Rodríguez*[a] and Tomás Rocha-Rinza*[a]

[a]Institute of Chemistry, National Autonomous University of Mexico, Circuito Exterior, Ciudad Universitaria, Delegación Coyoacán C.P. 04510, Mexico City, Mexico.

[b]Present address: Department of Chemistry, University of British Columbia, Okanagan, 3247 University Way, Kelowna, British Columbia, Canada V1V 1V7.

*Email: marcoshr@unam.mx, tomasrocharinza@gmail.com

Table of Contents

1. Experimental section S3
  1.1 General procedure for ^{1}H-NMR titrations S3
  1.2 Self-association constants of 2-pyrrolidone in different solvents S4
  1.3 Self-association studies in $CDCl_3$ S5
  1.4 Heterodimerisation of **A5** and **I1** in $CDCl_3$ S17
  1.5 Heterodimerisation of **A1** and **I2** in $CDCl_3$ S18
  1.6 ^{1}H-DOSY experiments S19
2. Computational details S32
  2.1 QTAIM molecular graphs for the examined amide and imide dimers and heterodimers S33
    2.1.1 Homodimers S33
    2.1.2 Heterodimers S36
  2.2 Hydrogen bond formation energies by Espinosa's empirical formula S37
  2.3 Use of the interacting quantum atoms approach for the study of bimolecular clusters S38
    2.3.1 IQA analysis of **I1-A5** and **I2-A1** heterodimers as well as **I1** and **I2** homodimers S40
  2.4 Data for the correlation between $|E(\mathrm{A})|$ vs pK_a and $|E(\mathrm{B})|$ vs pK_{BH+} S44
  2.5 Hydrogen bonds between **A2** and a chloroform molecule S47
  2.6 Correlation of experimental and that computed with the first-degree model herein S48
  2.7 Acidity and basicity values of the investigated systems in CCl_4 S49
  2.8 Delocalisation indices S50
  2.9 Bond lengths S51
  2.10 XYZ coordinates and electronic energies S52
    2.10.1 Compounds studied in $CDCl_3$ S53
    2.10.2 Heterodimers of **I1-A5** and **I2-A1** S167
    2.10.3 **A2···$CHCl_3$** complexes S171
    2.10.4 Compounds studied in CCl_4 S173
3. References S214

1. Experimental section

Compounds **A1**, **A2**, **A4-A7**, **I1**, **I2**, **I4**, **I5** and **I8** are commercially available and only **I3** was synthesised according to the procedure reported by Fun et al.[1] 2-pyrrolidone (**A1**) was further purified by distillation to remove water and other impurities. The dimerisation constants for the rest of systems in Table 3 and all of the molecules in Figure 7 were obtained from the literature.

1.1 General procedure for ^{1}H-NMR titrations

NMR spectra were recorded in a range of 0.002 to 1.0 M at 25 °C in $CDCl_3$. Only compounds **A6** and **A7** were studied in a range of $8.0 \cdot 10^{-4}$ M to 0.08 M because of their low solubility in $CDCl_3$. All experiments were performed on a 300 MHz spectrometer and N-H chemical shifts are reported in ppm downfield from TMS. ^{1}H-NMR spectra were processed using the MestReNova NMR software.[2] The association constants were calculated from the downfield shifting of the N-H proton using the online tool supramolecular.org.[3] The self-association constants of the investigated compounds are shown in Table 3. Heterodimerisation constants were calculated with the HypNMR 2008 program.[4]

1.2 Self-association constants of 2-pyrrolidone in different solvents

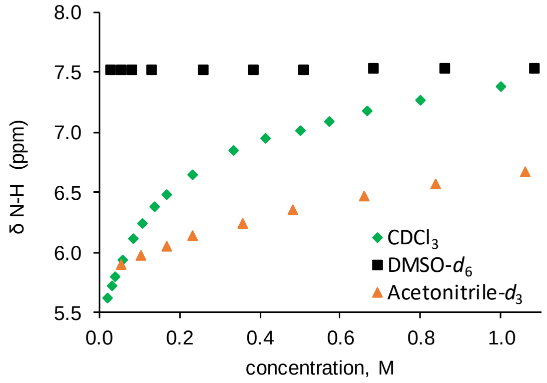

Figure S1. Profile of chemical shift as a function of concentration for 2-pyrrolidone, **A1**, in different solvents. The concentration range of **A1** from 0.002 M to 1.0 M at 25 ^{o}C. The dimerisation constants are reported in Table 1 in the main body of the paper. The percentage errors are: ($CDCl_3$) ± 0.4% and (Acetonitrile-d_3) ± 0.6%. The values of K_{dimer} in DMSO-d_6 could not be determined with good accuracy in virtue of its small value.

1.3 Self-association studies in $CDCl_3$

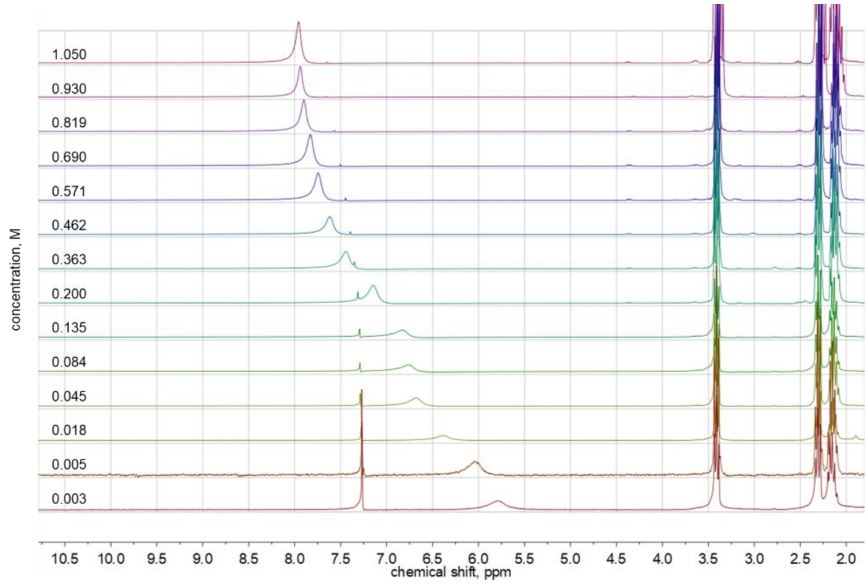

(a)

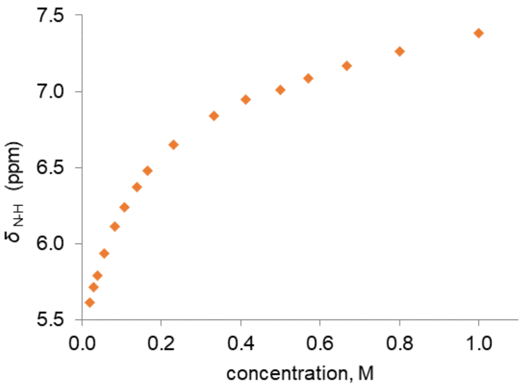

K_{dimer}	error
2.7 M^{-1}	± 0.4 %
$\delta_{N\text{-}H}$ (ppm)	
monomer	dimer
5.34	8.46

$\Delta\delta_{N\text{-}H}$ = 3.12

(b)

Figure S2. (a) Stacked plot of ^{1}H-NMR (300 MHz) spectra for the self-association study of **A1** at different concentrations and (b) its profile of chemical shift as a function of concentration as well as its dimerisation constant, the corresponding percentage error and values of $\delta_{N\text{-}H}$ (dimer and monomer) in $CDCl_3$ at 25 ^{o}C.

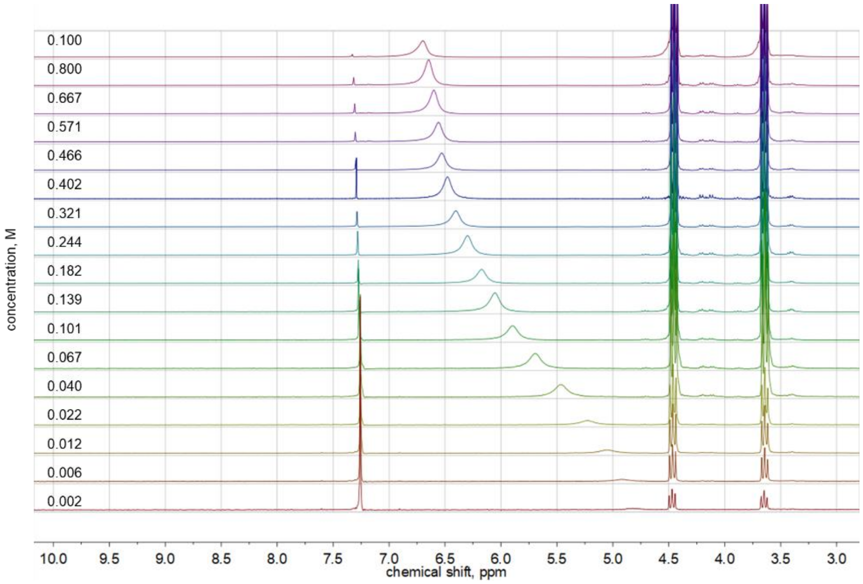

(a)

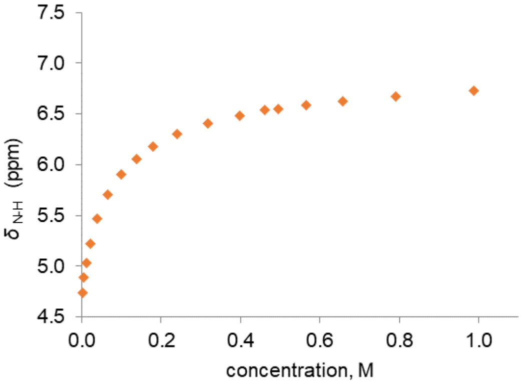

K_{dimer}	error
8.3 M^{-1}	± 1.0 %
$\delta_{\text{N-H}}$ (ppm)	
monomer	dimer
4.65	7.34

$\Delta\delta_{\text{N-H}}$ = 2.68

(b)

Figure S3. (a) Stacked plot of ^{1}H-NMR (300 MHz) spectra for the self-association study of **A2** at different concentrations and (b) its profile of chemical shift as a function of concentration as well as its dimerisation constant, the corresponding percentage error and values of $\delta_{\text{N-H}}$ (dimer and monomer) in $CDCl_3$ at 25 ^{o}C.

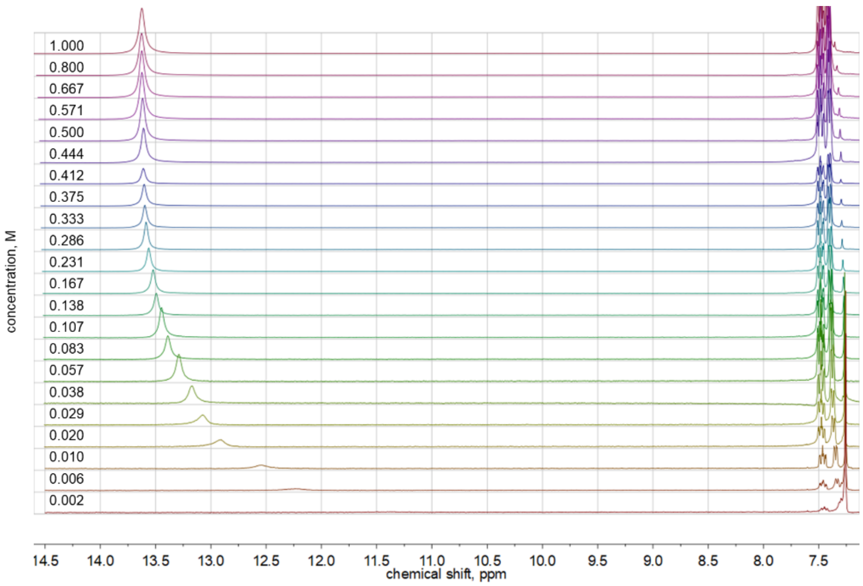

(a)

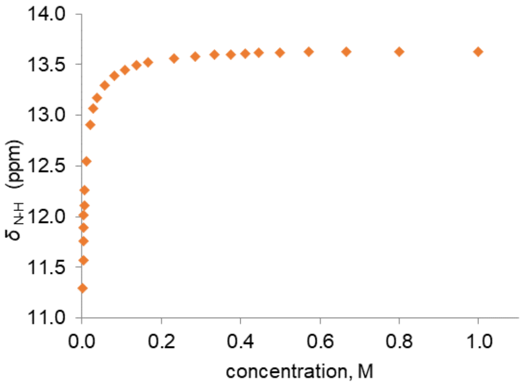

K_{dimer}	error
740.0 M^{-1}	± 1.2 %
δ_{N-H} (ppm)	
monomer	dimer
8.15	13.84

$\Delta\delta_{N-H}$ = 5.69

(b)

Figure S4. (a) Stacked plot of ^{1}H-NMR (300 MHz) spectra for the self-association study of **A4** at different concentrations and (b) its profile of chemical shift as a function of concentration as well as its dimerisation constant, the corresponding percentage error and values of δ_{N-H} (dimer and monomer) in $CDCl_3$ at 25 °C.

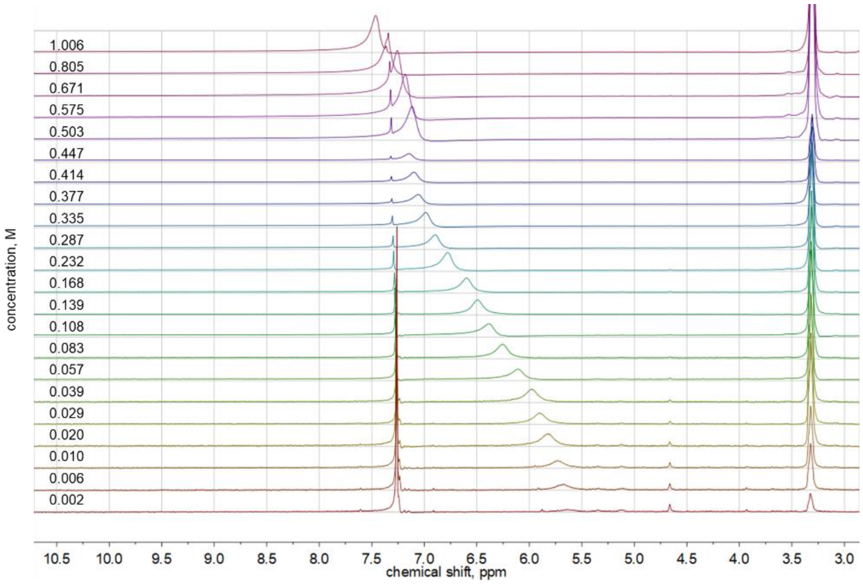

(a)

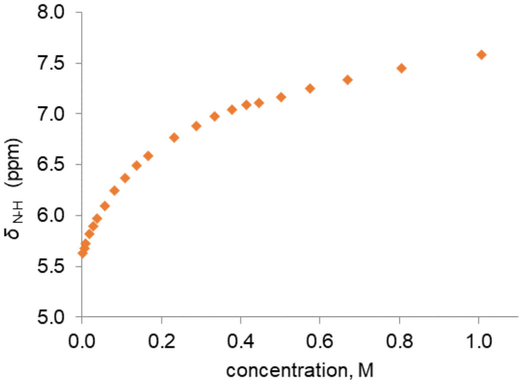

K_{dimer}	error
1.8 M^{-1}	± 0.5 %

$\delta_{\text{N-H}}$ (ppm)

monomer	dimer
5.61	8.88

$\Delta\delta_{\text{N-H}} = 3.27$

(b)

Figure S5. (a) Stacked plot of ^{1}H-NMR (300 MHz) spectra for the self-association study of **A5** at different concentrations and (b) its profile of chemical shift as a function of concentration as well as its dimerisation constant, the corresponding percentage error and values of $\delta_{\text{N-H}}$ (dimer and monomer) in $CDCl_3$ at 25 °C.

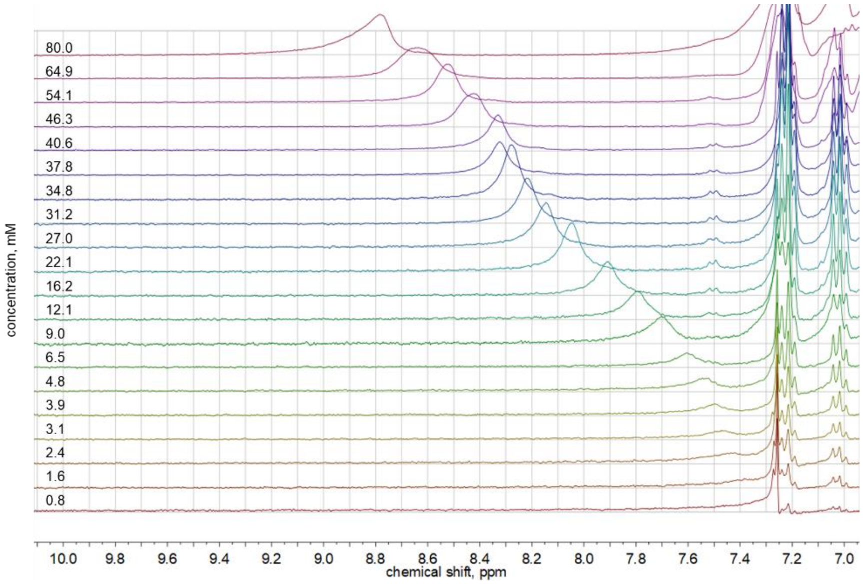

(a)

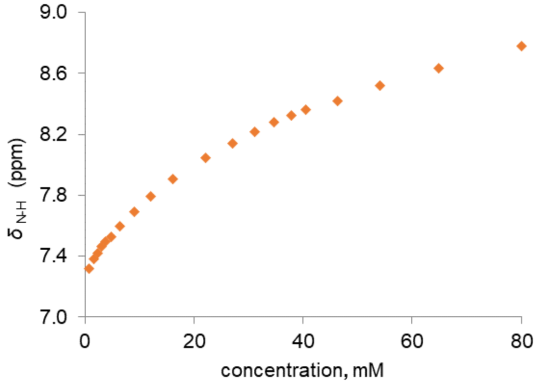

K_{dimer}	error
8.0 M^{-1}	± 0.7 %

$\delta_{\text{N-H}}$ (ppm)	
monomer	dimer
7.30	10.74

$\Delta\delta_{\text{N-H}}$ = 3.44

(b)

Figure S6. (a) Stacked plot of ^{1}H-NMR (300 MHz) spectra for the self-association study of **A6** at different concentrations and (b) its profile of chemical shift as a function of concentration as well as its dimerisation constant, the corresponding percentage error and values of $\delta_{\text{N-H}}$ (dimer and monomer) in $CDCl_3$ at 25 ^oC.

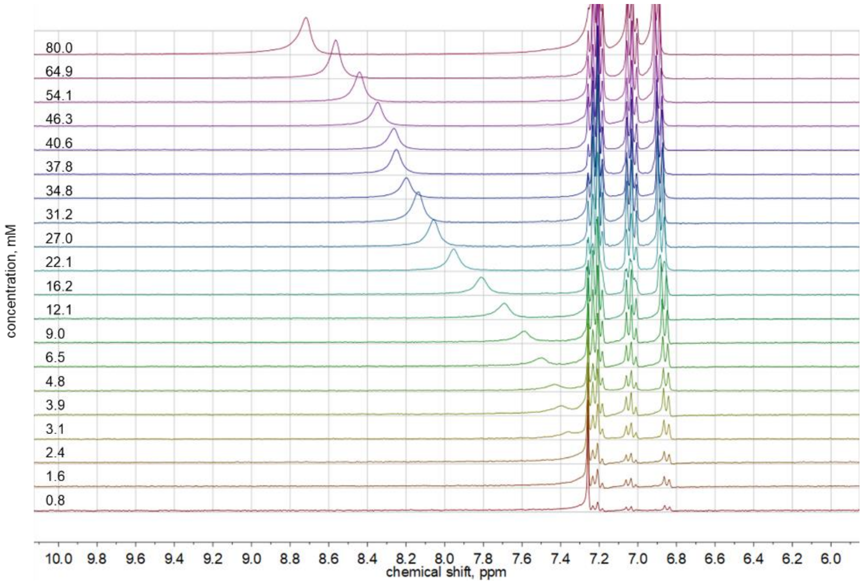

(a)

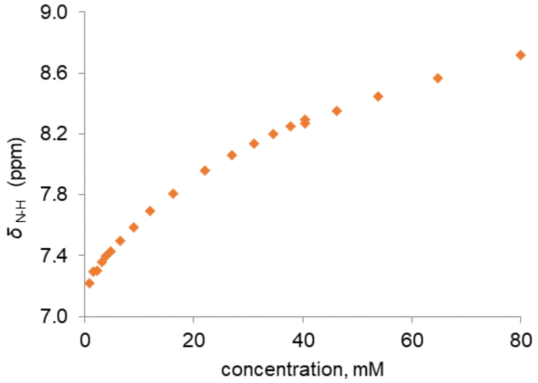

K_{dimer}	error
7.6 M^{-1}	± 0.6 %
δ_{N-H} (ppm)	
monomer	dimer
7.19	10.82

$\Delta\delta_{N-H}$ = 3.63

(b)

Figure S7. (a) Stacked plot of ^{1}H-NMR (300 MHz) spectra for the self-association study of **A7** at different concentrations and (b) its profile of chemical shift as a function of concentration as well as its dimerisation constant, the corresponding percentage error and values of δ_{N-H} (dimer and monomer) in $CDCl_3$ at 25 °C.

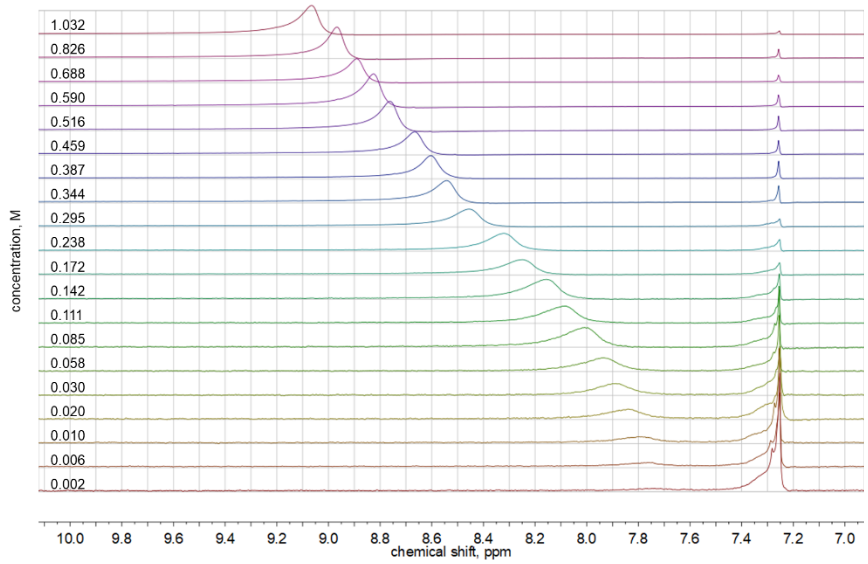

(a)

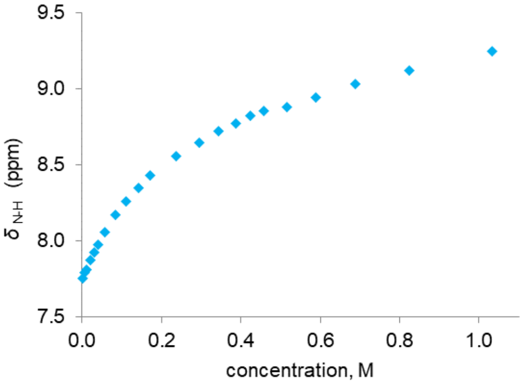

K_{dimer}	error
1.4 M^{-1}	± 0.7 %
δ_{N-H} (ppm)	
monomer	dimer
7.73	10.45

$\Delta\delta_{N-H}$ = 2.72

(b)

Figure S8. (a) Stacked plot of ^{1}H-NMR (300 MHz) spectra for the self-association study of **I1** at different concentrations and (b) its profile of chemical shift as a function of concentration as well as its dimerisation constant, the corresponding percentage error and values of δ_{N-H} (dimer and monomer) in $CDCl_3$ at 25 ^oC.

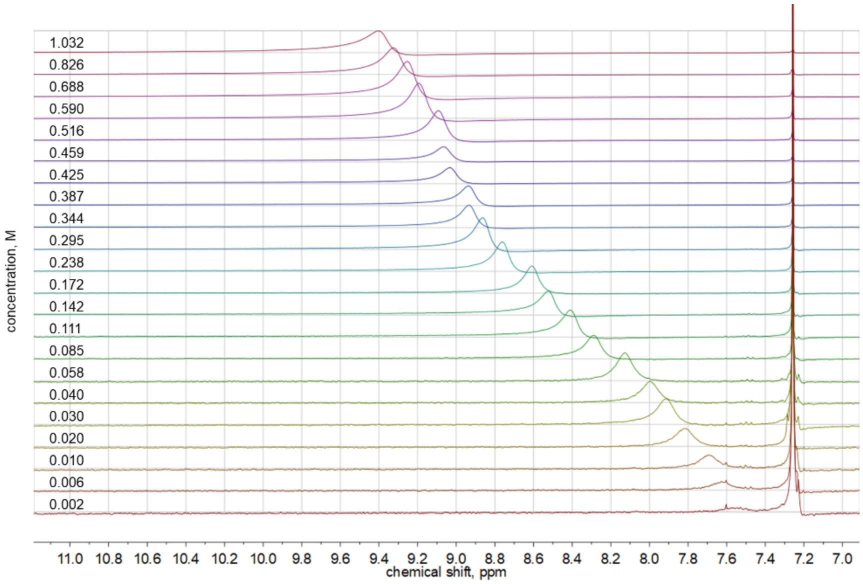

(a)

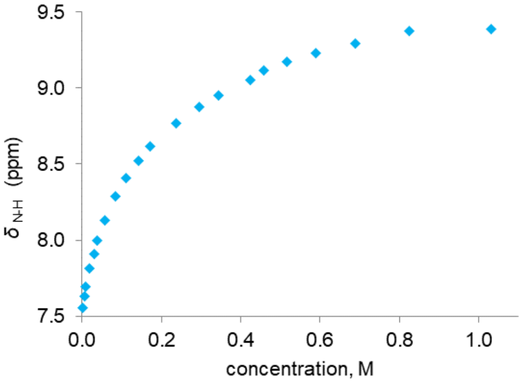

K_{dimer}	error
3.3 M^{-1}	± 0.5 %
$\delta_{N\text{-}H}$ (ppm)	
monomer	dimer
7.49	10.30

$\Delta\delta_{N\text{-}H}$ = 2.81

(b)

Figure S9. (a) Stacked plot of ^{1}H-NMR (300 MHz) spectra for the self-association study of **I2** at different concentrations and (b) its profile of chemical shift as a function of concentration as well as its dimerisation constant, the corresponding percentage error and values of $\delta_{N\text{-}H}$ (dimer and monomer) in $CDCl_3$ at 25 °C.

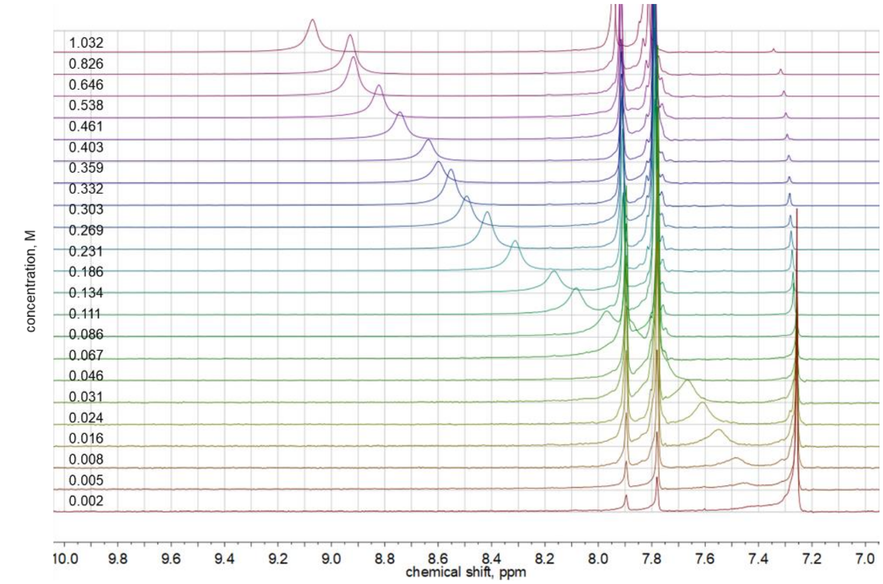

(a)

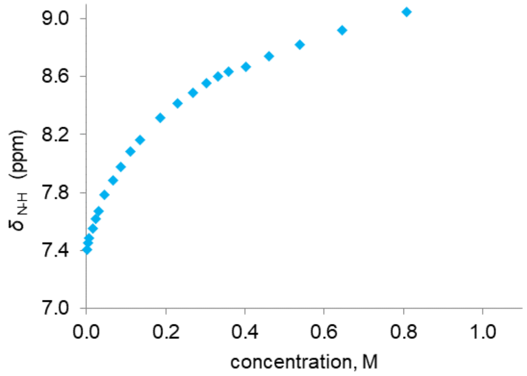

K_{dimer}	error
2.1 M^{-1}	± 1.3 %
$\delta_{N\text{-}H}$ (ppm)	
monomer	dimer
7.40	10.12

$\Delta\delta_{N\text{-}H}$ = 2.71

(b)

Figure S10. (a) Stacked plot of ^{1}H-NMR (300 MHz) spectra for the self-association study of **I3** at different concentrations and (b) its profile of chemical shift as a function of concentration as well as its dimerisation constant, the corresponding percentage error and values of $\delta_{N\text{-}H}$ (dimer and monomer) in $CDCl_3$ at 25 °C.

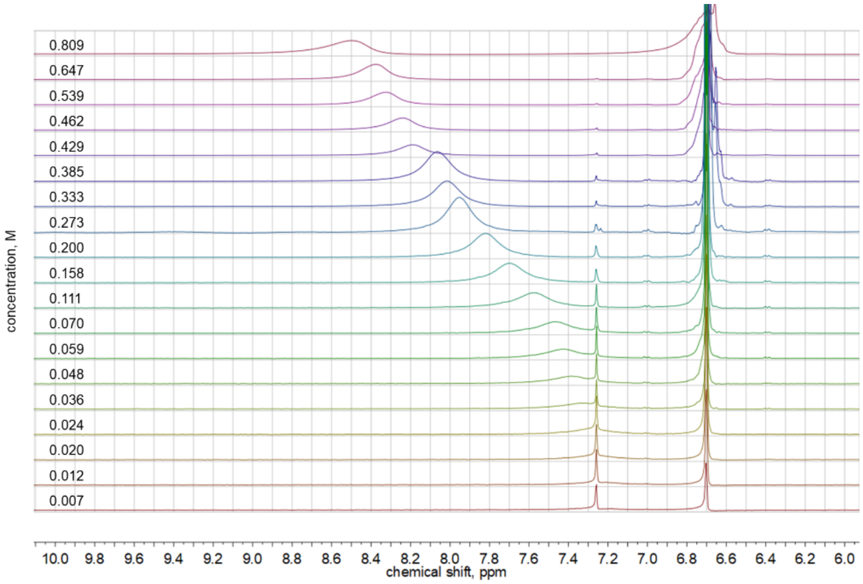

(a)

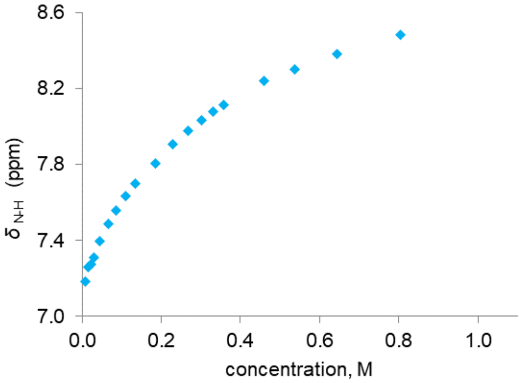

K_{dimer}	error
1.2 M^{-1}	± 0.7 %
$\delta_{N\text{-}H}$ (ppm)	
monomer	dimer
7.15	9.87

$\Delta\delta_{N\text{-}H}$ = 2.72

(b)

Figure S11. (a) Stacked plot of ^{1}H-NMR (300 MHz) spectra for the self-association study of **I4** at different concentrations and (b) its profile of chemical shift as a function of concentration as well as its dimerisation constant, the corresponding percentage error and values of $\delta_{N\text{-}H}$ (dimer and monomer) in $CDCl_3$ at 25 ^{o}C.

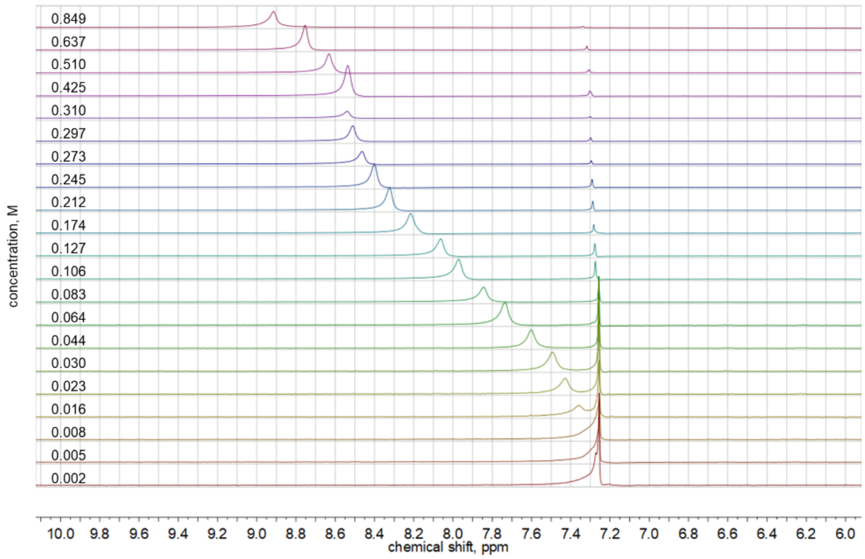

(a)

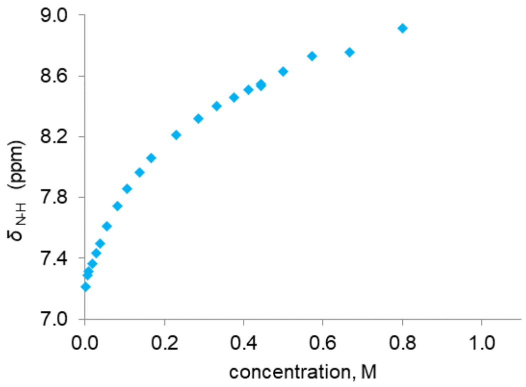

K_{dimer}	error
2.6 M^{-1}	± 1.7 %

$\delta_{N\text{-}H}$ (ppm)	
monomer	dimer
7.18	9.99

$\Delta\delta_{N\text{-}H}$ = 2.81

(b)

Figure S12. (a) Stacked plot of ^{1}H-NMR (300 MHz) spectra for the self-association study of **I5** at different concentrations and (b) its profile of chemical shift as a function of concentration as well as its dimerisation constant, the corresponding percentage error and values of $\delta_{N\text{-}H}$ (dimer and monomer) in $CDCl_3$ at 25 °C.

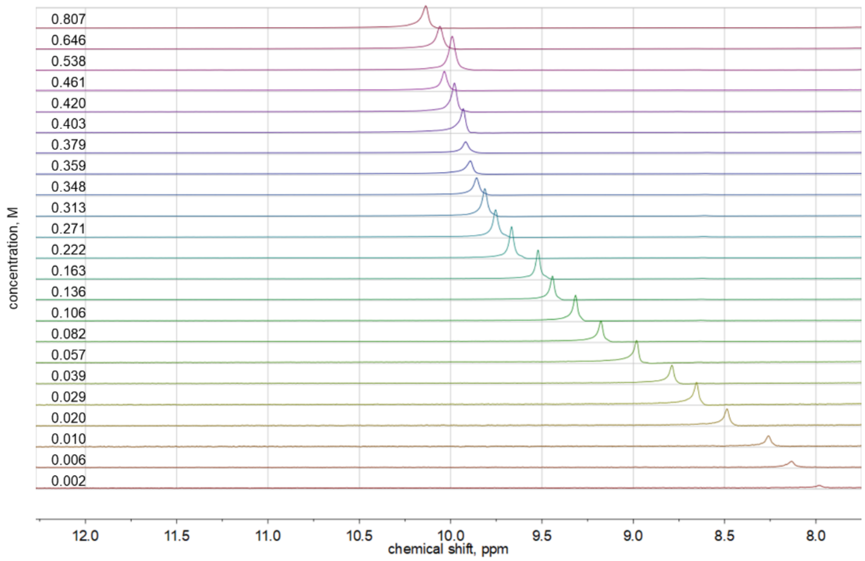

(a)

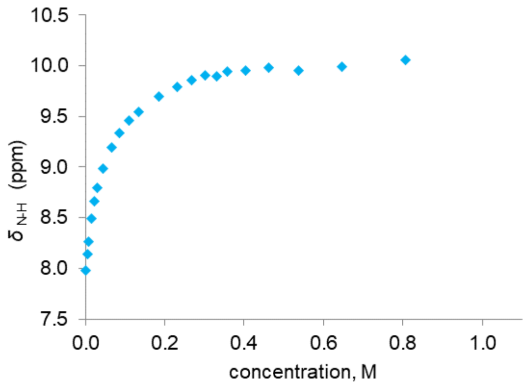

K_{dimer}	error
8.6 M^{-1}	± 1.6 %
δ_{N-H} (ppm)	
monomer	dimer
7.87	10.88

$\Delta\delta_{N-H}$ = 3.01

(b)

Figure S13. (a) Stacked plot of ^{1}H-NMR (300 MHz) spectra for the self-association study of **I8** at different concentrations and (b) its profile of chemical shift as a function of concentration as well as its dimerisation constant, the corresponding percentage error and values of δ_{N-H} (dimer and monomer) in $CDCl_3$ at 25 ^oC.

1.4 Heterodimerisation of A5 and I1 in $CDCl_3$

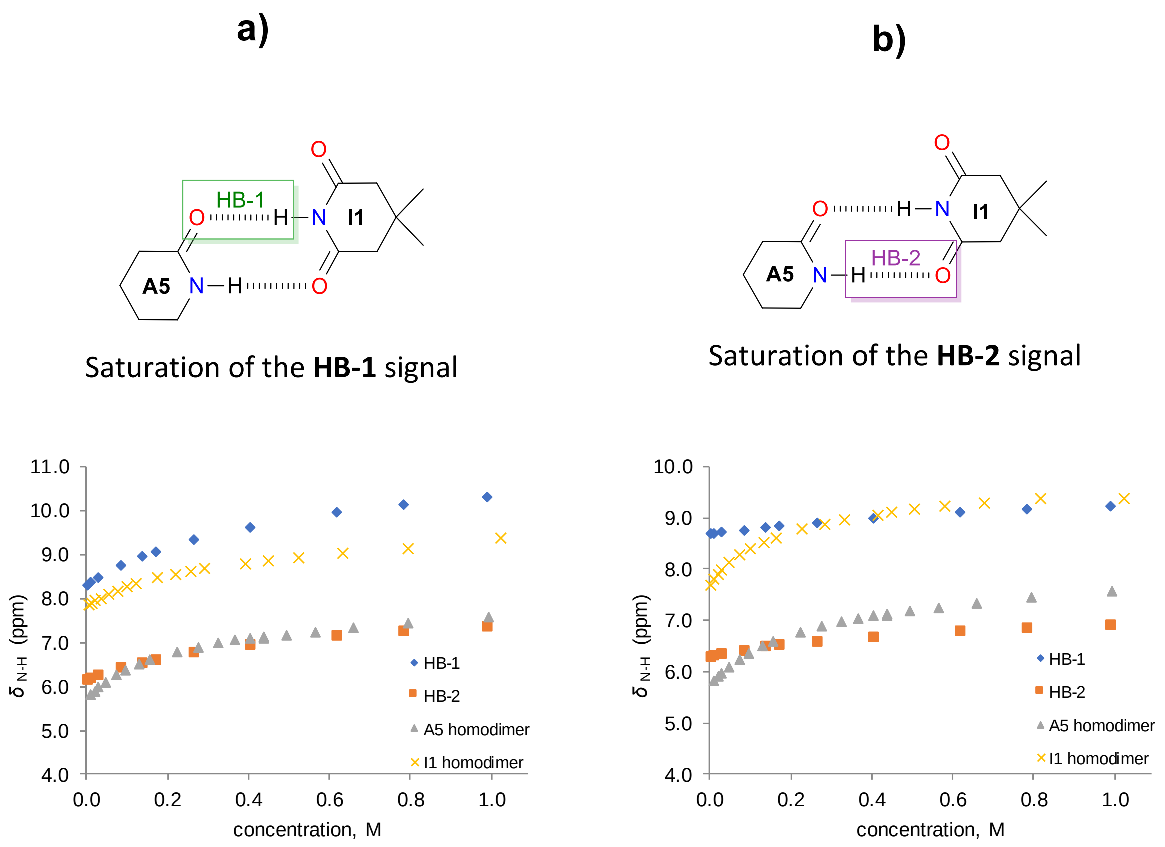

K_{heter1} = 4.4 ± 1.2 M^{-1}

K_{heter2} = 5.0 ± 1.1 M^{-1}

K_{heter} = 4.7 ± 1.1 M^{-1} (**K_{heter} corr = 2.4 M^{-1}**)

$\delta_{N\text{-}H}$ HB-1 = 11.43 ppm $\delta_{N\text{-}H}$ HB-2 = 7.36 ppm

Figure S14. Profile of chemical shift as a function of concentration for the heterodimerisation **I1-A5** in $CDCl_3$ at 25 °C: a) imide **I1** (0.1 M) upon titration with amide **A5** (0-20 eq.) and b) vice versa. The heterodimerisation constants with and without statistical factor corrections are also shown.

1.5 Heterodimerisation of A1 and I2 in $CDCl_3$

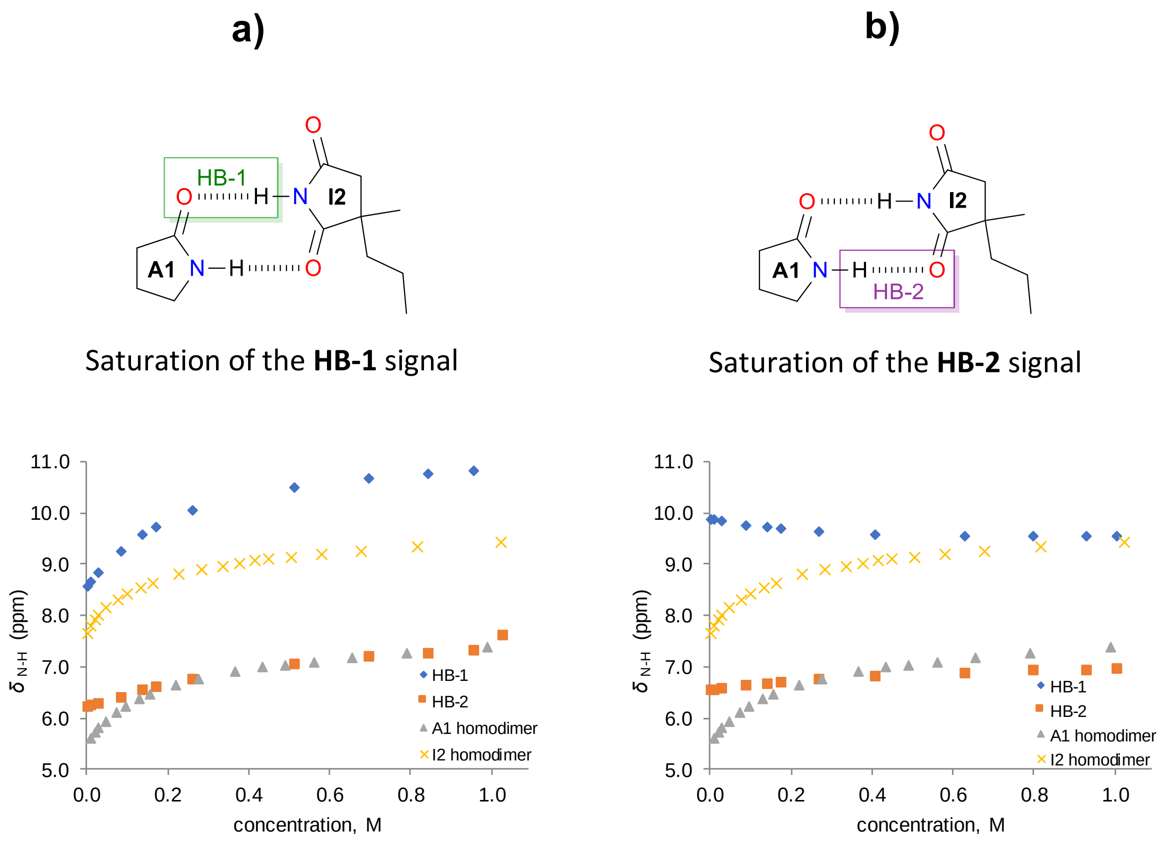

K_{heter1} = 9.7 ± 1.1 M^{-1} K_{heter2} = 8.5 ± 1.1 M^{-1}

$\boldsymbol{K_{heter}}$ = 9.1 ± 1.1 M^{-1} (**$\boldsymbol{K_{heter}}$ corr = 4.5 $\mathbf{M^{-1}}$)**

δ_{N-H} HB-1 = 12.19 ppm δ_{N-H} HB-2 = 7.45 ppm

Figure S15. Profile of chemical shift as a function of concentration for the heterodimerisation **I2-A1** in $CDCl_3$ at 25 °C: a) imide **I2** (0.1 M) upon titration with amide **A1** (0-20 eq.) and b) vice versa. The heterodimerisation constants with and without statistical factor corrections are also shown.

1.6 ^{1}H-DOSY experiments

Diffusion Ordered Spectroscopy (^{1}H-DOSY) experiments were recorded at 25 °C, using a 2D pulse sequence (dstebpgp3s) for diffusion measurements with double stimulated echo for convection compensation. The experiment involves bipolar gradient pulses, a Longitudinal Eddy Current Delay (LED) and three spoil gradients. The standard pulse sequence dstebpgp3s, was taken from the Bruker software library. The obtained diffusion coefficients allow to estimate the size of complexes or adducts through the calculation of hydrodynamic radius by considering the tetramethylsilane (TMS) as an internal standard reference, r_{ref}, from the following equation:

$$r_H = \frac{D_{ref}}{D} r_{ref} ,$$

where D_{ref} and D are the diffusion coefficients of TMS and the sample, respectively.

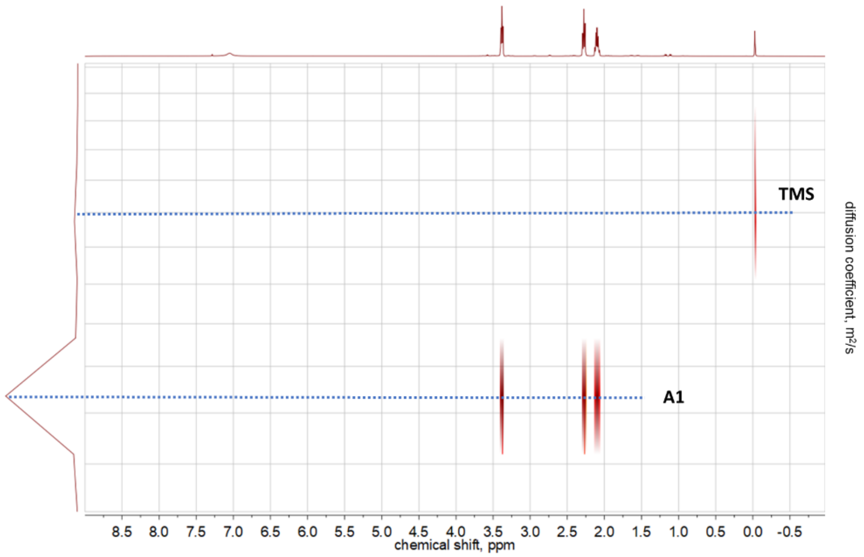

Concentration: 0.5 M

Diffusion coefficients

(10^{-2} m^2/s)

D	D_{ref},
**A1**	**TMS**
12.05	16.88

(a)

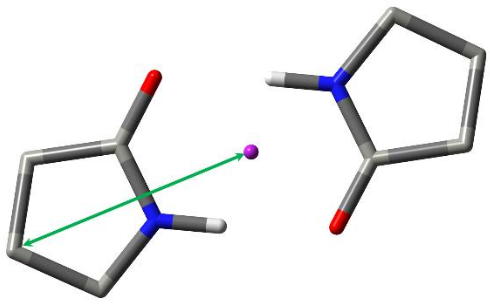

(b)

Figure S16. (a) ^{1}H-DOSY (500 MHz) spectrum in $CDCl_3$ at 25 ^{o}C and (b) dimer structure determined via M06-2x/6-311++G(2d,2p) calculations of amide **A1**. Experimental and calculated radii are shown in Table 2 in the body of the paper.

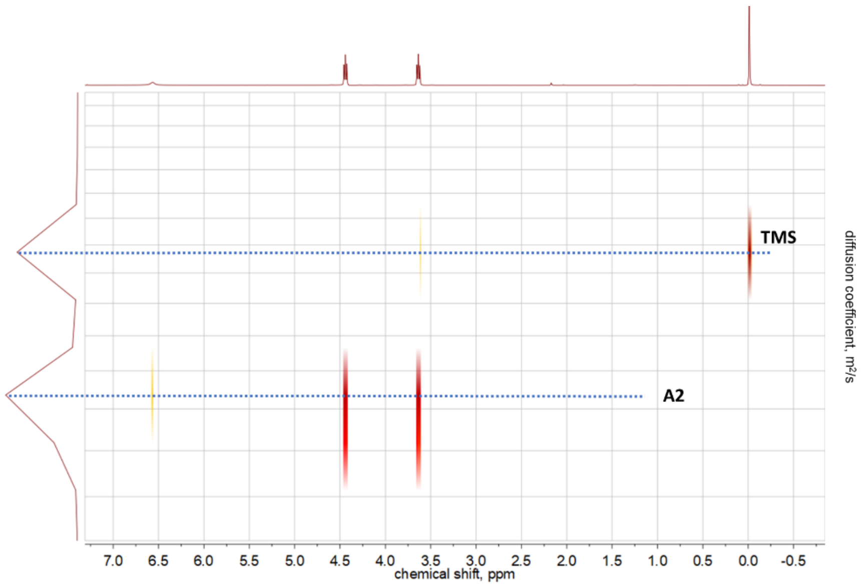

Concentration: 0.5 M

Diffusion coefficients

(10^{-2} m^2/s)

D	D_{ref},
**A2**	**TMS**
11.67	16.72

(a)

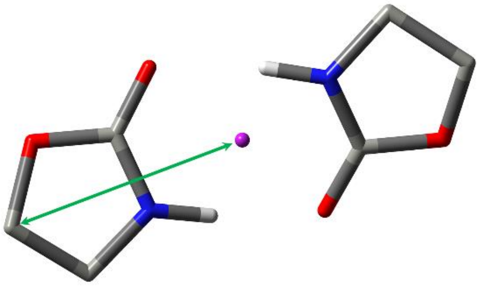

(b)

Figure S17. (a) ^{1}H-DOSY (500 MHz) spectrum in $CDCl_3$ at 25 ^oC and (b) dimer structure determined via M06-2x/6-311++G(2d,2p) calculations of amide **A2**. Experimental and calculated radii are shown in Table 2 in the body of the paper.

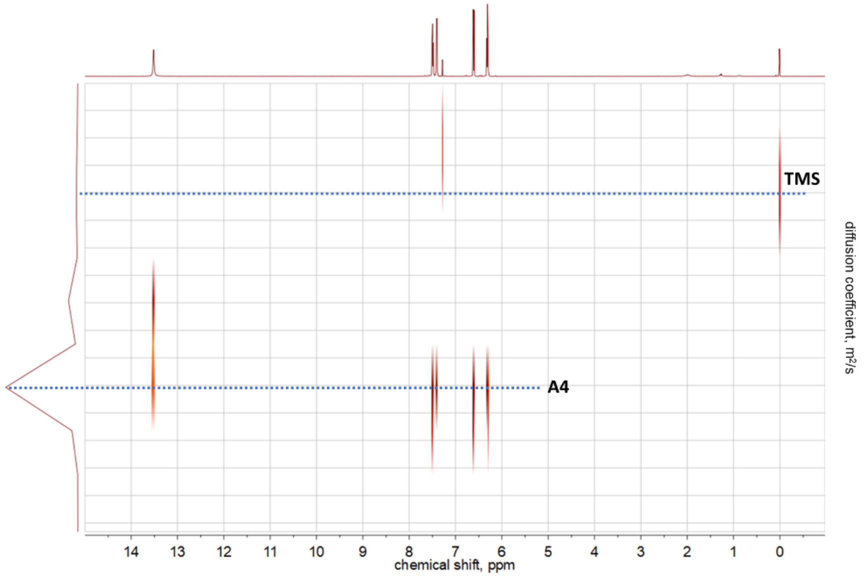

Concentration: 0.6 M

Diffusion coefficients

(10^{-2} m^2/s)

D	D_{ref},
**A4**	**TMS**
10.32	17.39

(a)

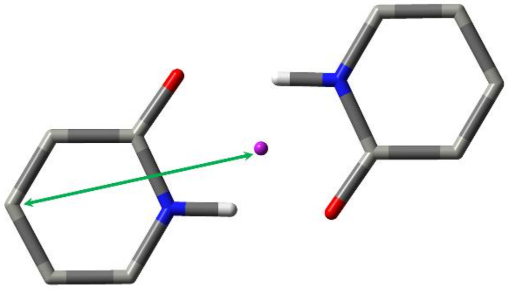

(b)

Figure S18. (a) ^{1}H-DOSY (500 MHz) spectrum in $CDCl_3$ at 25 °C and (b) dimer structure determined via M06-2x/6-311++G(2d,2p) calculations of amide **A4**. Experimental and calculated radii are shown in Table 2 in the body of the paper.

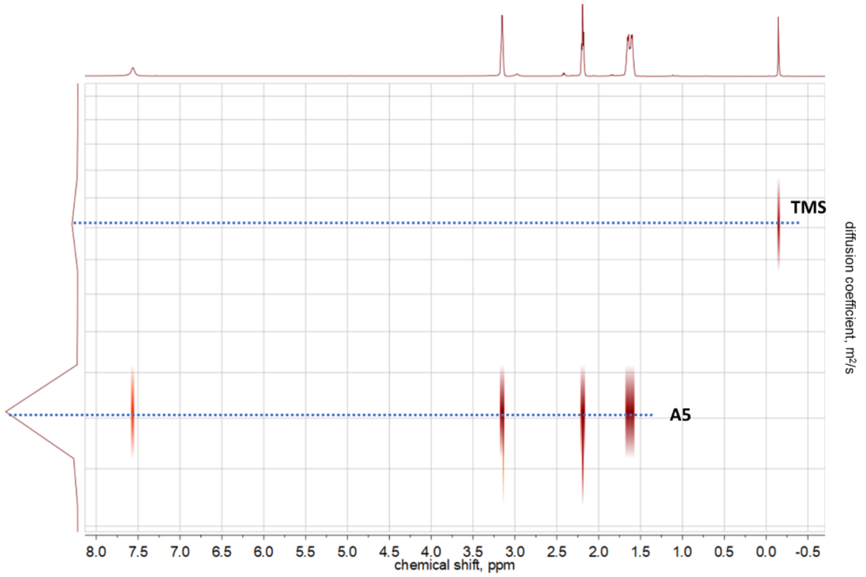

Concentration: 0.6 M

Diffusion coefficients

(10^{-2} m^2/s)

D	D_{ref},
**A5**	**TMS**
9.48	15.46

(a)

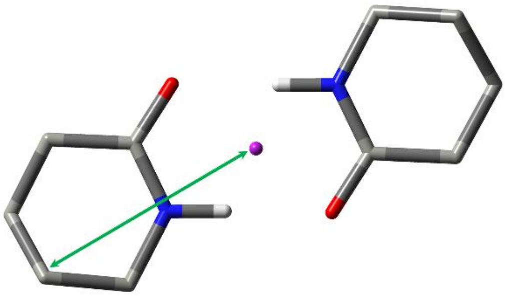

(b)

Figure S19. (a) ^{1}H-DOSY (500 MHz) spectrum in $CDCl_3$ at 25 °C and (b) dimer structure determined via M06-2x/6-311++G(2d,2p) calculations of amide **A5**. Experimental and calculated radii are shown in Table 2 in the body of the paper.

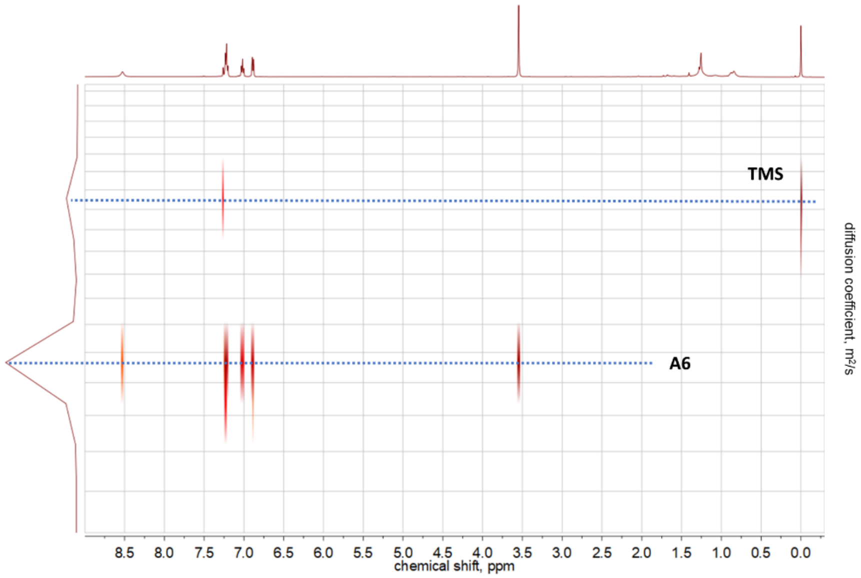

Concentration: 0.08 M

Diffusion coefficients

(10^{-2} m^2/s)

D	D_{ref},
**A6**	**TMS**
13.19	19.93

(a)

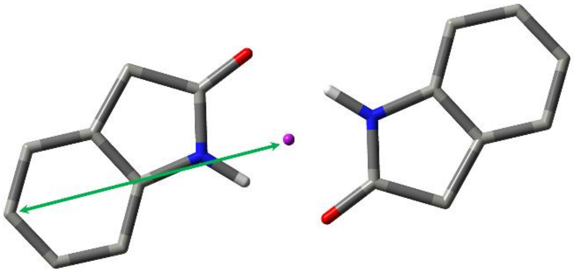

(b)

Figure S20. (a) ^{1}H-DOSY (500 MHz) spectrum in $CDCl_3$ at 25 °C and (b) dimer structure determined via M06-2x/6-311++G(2d,2p) calculations of amide **A6**. Experimental and calculated radii are shown in Table 2 in the body of the paper.

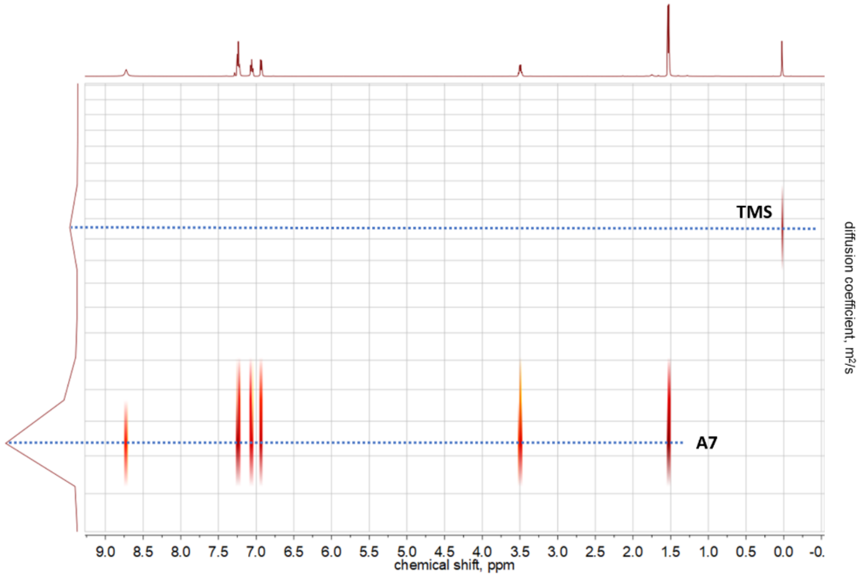

Concentration: 0.08 M

Diffusion coefficients

(10^{-2} m^2/s)

D	D_{ref},
**A7**	**TMS**
12.37	20.42

(a)

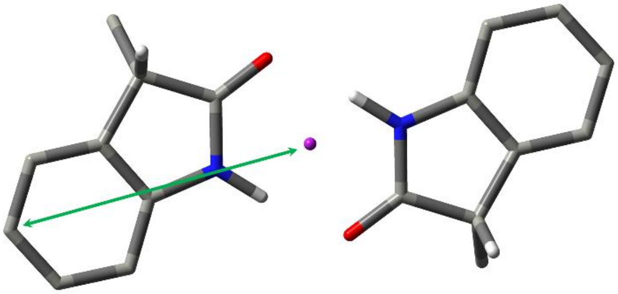

(b)

Figure S21. (a) ^{1}H-DOSY (500 MHz) spectrum in $CDCl_3$ at 25 °C and (b) dimer structure determined via M06-2x/6-311++G(2d,2p) calculations of amide **A7**. Experimental and calculated radii are shown in Table 2 in the body of the paper.

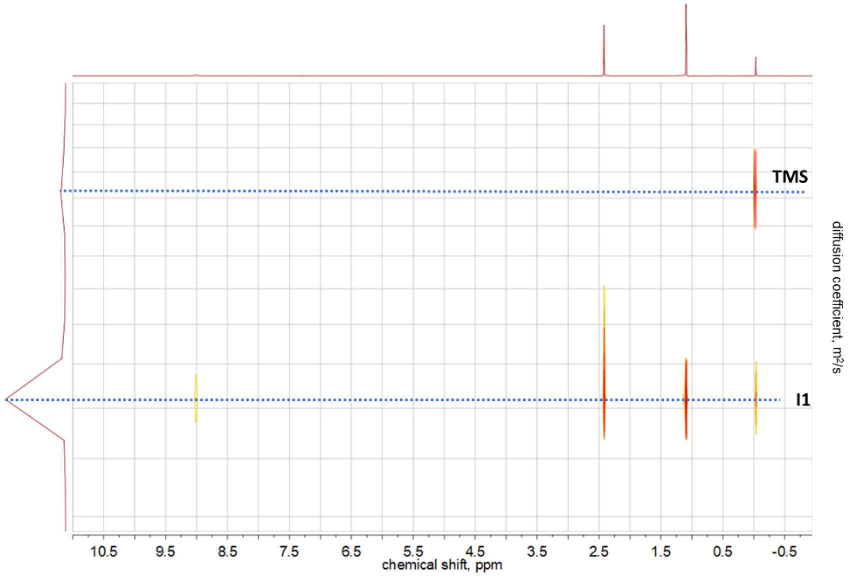

Concentration: 0.6 M

Diffusion coefficients

(10^{-2} m^2/s)

D	D_{ref},
**I1**	**TMS**
8.72	15.19

(a)

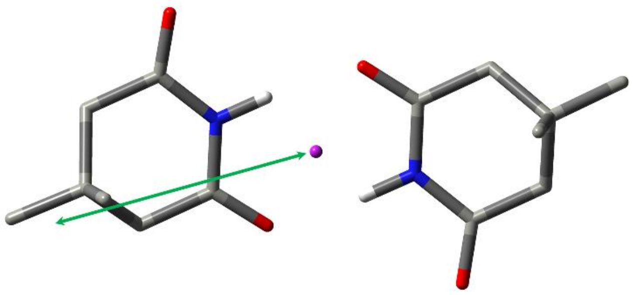

(b)

Figure S22. (a) ^{1}H-DOSY (500 MHz) spectrum in $CDCl_3$ at 25 °C and (b) dimer structure determined via M06-2x/6-311++G(2d,2p) calculations of imide **I1**. Experimental and calculated radii are shown in Table 2 in the body of the paper.

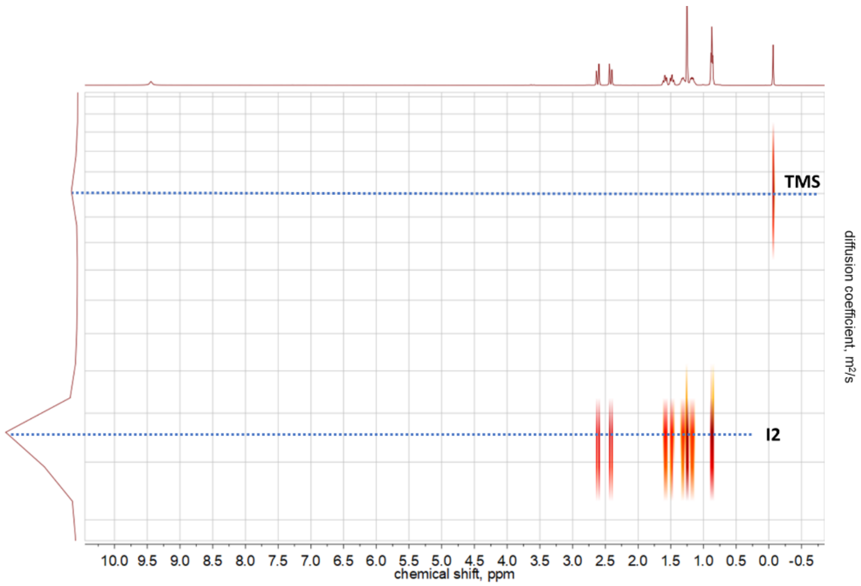

Concentration: 0.5 M

Diffusion coefficients

(10^{-2} m^2/s)

D	D_{ref},
**I2**	**TMS**
6.75	15.04

(a)

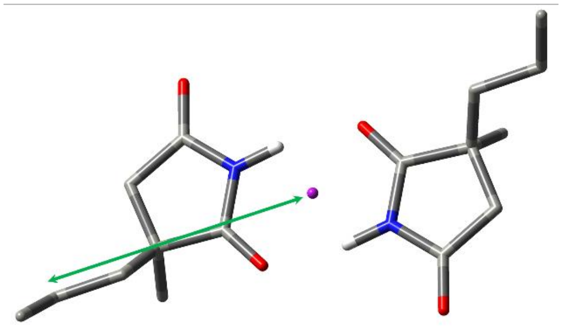

(b)

Figure S23. (a) ^{1}H-DOSY (500 MHz) spectrum in $CDCl_3$ at 25 °C and (b) dimer structure determined via M06-2x/6-311++G(2d,2p) calculations of imide **I2**. Experimental and calculated radii are shown in Table 2 in the body of the paper.

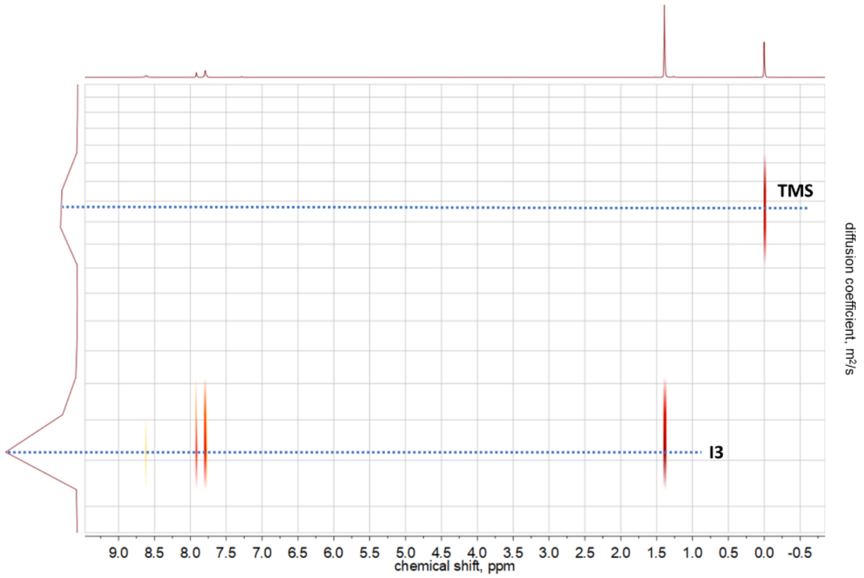

Concentration: 0.4 M

Diffusion coefficients

(10^{-2} m^2/s)

D	D_{ref},
**I3**	**TMS**
6.75	15.04

(a)

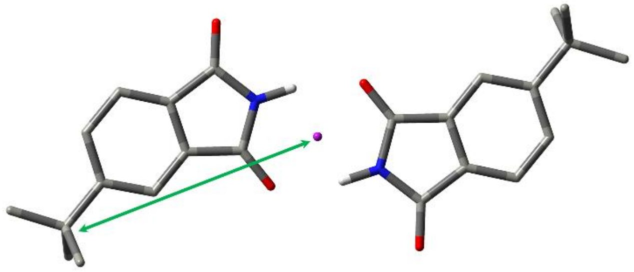

(b)

Figure S24. (a) ^{1}H-DOSY (500 MHz) spectrum in $CDCl_3$ at 25 °C and (b) dimer structure determined via M06-2x/6-311++G(2d,2p) calculations of imide **I3**. Experimental and calculated radii are shown in Table 2 in the body of the paper.

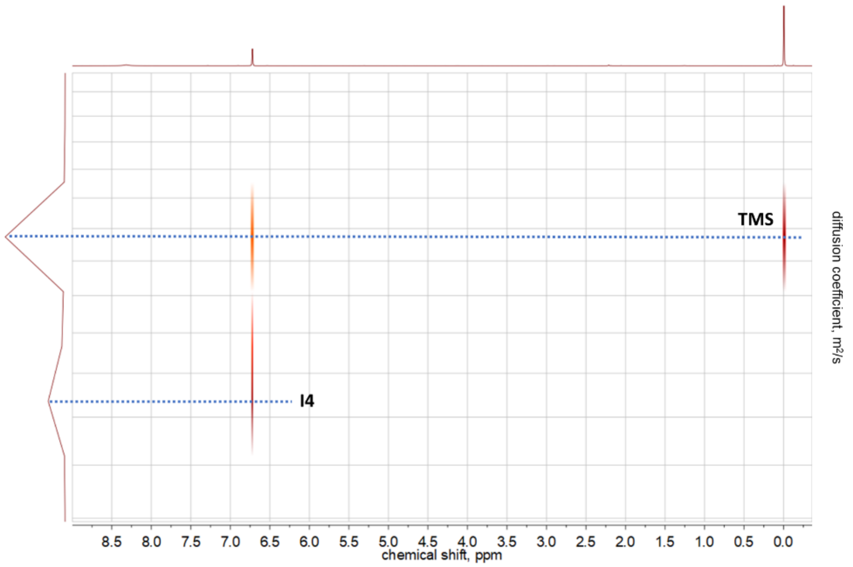

Concentration: 0.6 M

Diffusion coefficients

(10^{-2} m^2/s)

D	D_{ref},
**I4**	**TMS**
12.24	16.67

(a)

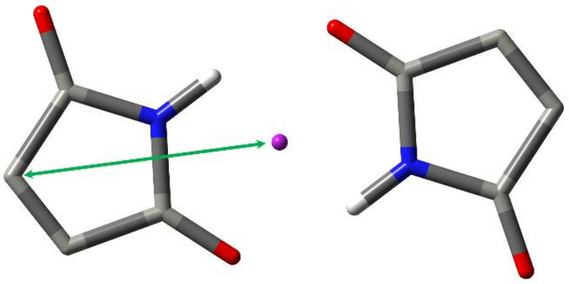

(b)

Figure S25. (a) ^{1}H-DOSY (500 MHz) spectrum in $CDCl_3$ at 25 ^{o}C and (b) dimer structure determined via M06-2x/6-311++G(2d,2p) calculations of imide **I4**. Experimental and calculated radii are shown in Table 2 in the body of the paper.

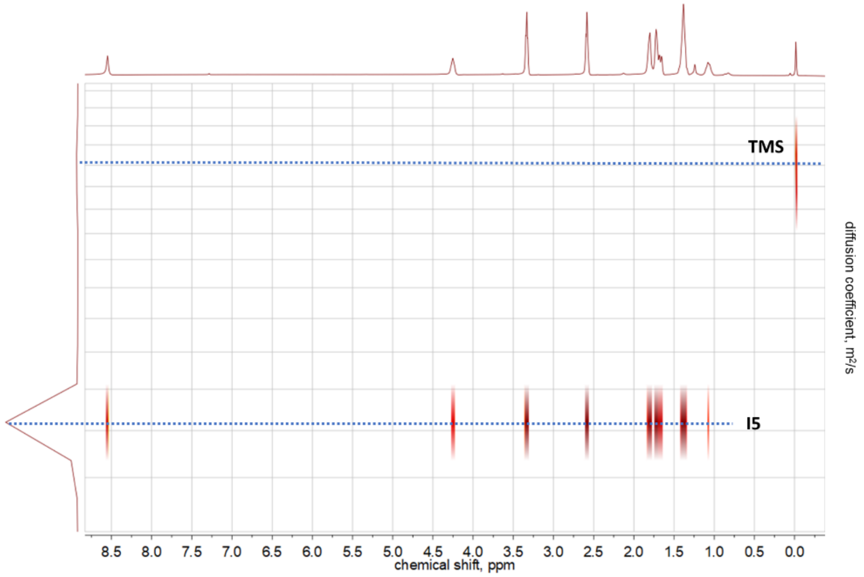

Concentration: 0.4 M

Diffusion coefficients

(10^{-2} m^2/s)

D	D_{ref},
**I5**	**TMS**
8.46	17.68

(a)

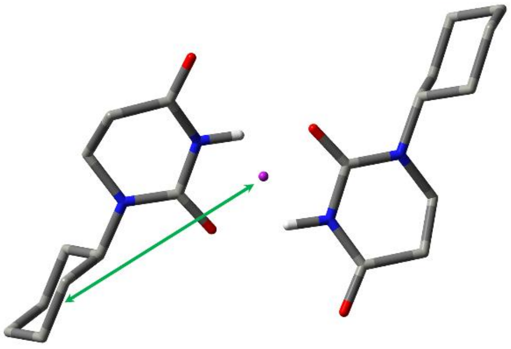

(b)

Figure S26. (a) ^{1}H-DOSY (500 MHz) spectrum in $CDCl_3$ at 25 °C and (b) dimer structure determined via M06-2x/6-311++G(2d,2p) calculations of imide **I5**. Experimental and calculated radii are shown in Table 2 in the body of the paper.

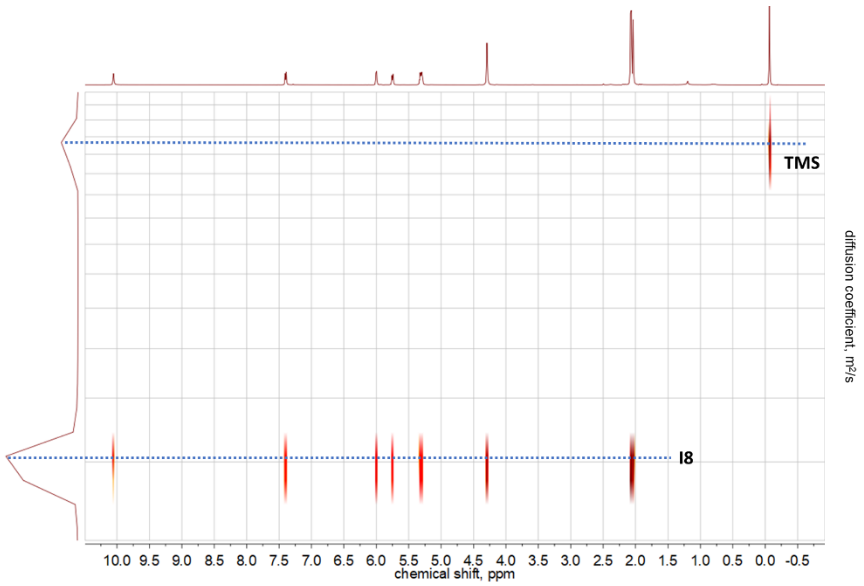

Concentration: 0.6 M

Diffusion coefficients

(10^{-2} m^2/s)

D	D_{ref},
**I8**	**TMS**
2.98	12.68

(a)

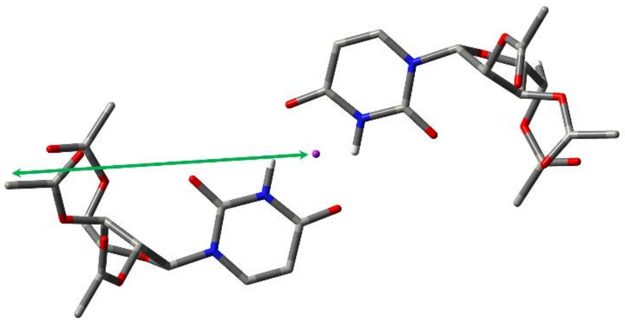

(b)

Figure S27. (a) ^{1}H-DOSY (500 MHz) spectrum in $CDCl_3$ at 25 °C and (b) dimer structure determined via M06-2x/6-311++G(2d,2p) calculations of imide **I8**. Experimental and calculated radii are shown in Table 2 in the body of the paper.

2. Computational details

All geometries of monomers, dimers and the species involved in the calculation of proton affinities were optimized using the M06-2x[5] functional with the 6-311++G(2d,2p) basis set as implemented in the Gaussian 09 package.[6] We chose this approximation because it yields a good description of the energetics of protonation and deprotonation processes and most importantly of intermolecular interactions such as hydrogen bonds.[5,7] Each stationary structure was characterised as a minimum via the calculation of the corresponding harmonic frequencies. The inclusion of nonspecific solvent effects in the calculations was made by using the SMD method.[8] Some earlier experimental and theoretical studies about the dimerisation of 2-pyridone,[9] 2-pyrrolidone,[10] δ-valerolactam[11] and maleimide[12,13] were taken as a starting point for this work. These studies conclude that the keto form is the most stable arrangement for the dimer formation. We studied the topology of the electron distribution under the formalism of the Quantum Theory of Atoms in Molecules (QTAIM) to get further insights about the chemical bonding scenario of the investigated dimers. This analysis was complemented with DFT electronic energy partitions in accordance with the Interacting Quantum Atoms (IQA) approach.[14] In particular, we examined the steric repulsion of the carbonyl groups (spectators and those involved in the HB) with both methods with the aid of the AIMAll[15] program. We also calculated QTAIM electron Delocalisation Indices DIs (Ω, Ω'), which are chemical bonding indicators that have been successfully used for the characterisation of non-covalent interactions like hydrogen bonds.[16]

2.1 QTAIM molecular graphs for the examined amide and imide dimers and heterodimers

We determined the molecular graphs for the considered amide and imide homo- and heterodimers. The bond and ring critical points are displayed respectively with green and red colour. The examined electron densities were computed with the (SMD-$CHCl_3$)-M06-2x//6-311++G(2d,2p) approximation.

2.1.1 Homodimers

Table S1. QTAIM molecular graphs of the amide and imide homodimers studied in this work. The bond and ring critical points are indicated with red and green colours respectively. **I11-I13** homodimers are not included because of the high computational cost of their corresponding calculations.

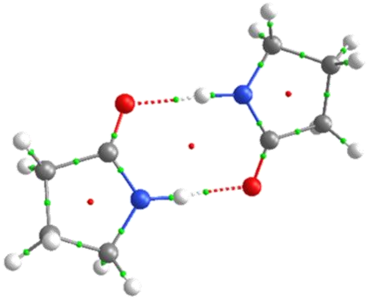<br>Amide **A1** homodimer	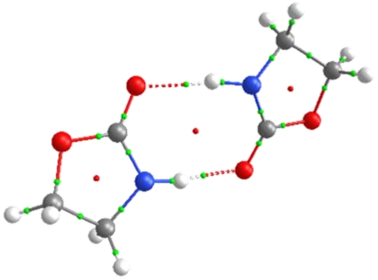<br>Amide **A2** homodimer
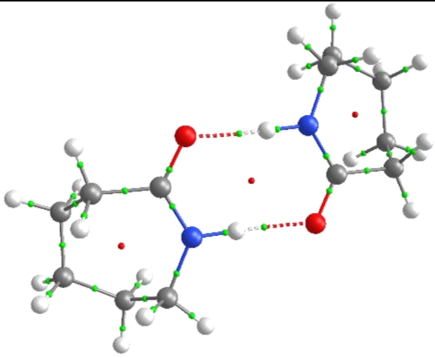<br>Amide **A3** homodimer	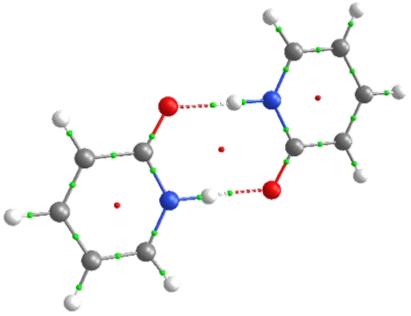<br>Amide **A4** homodimer

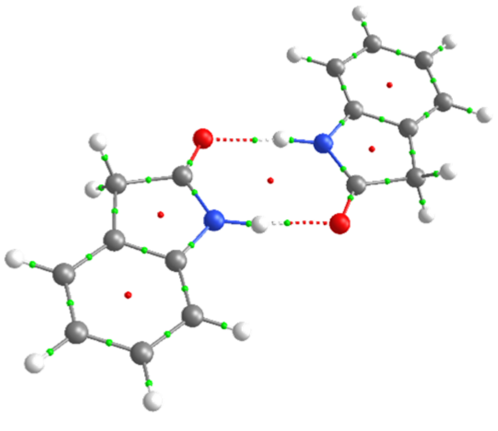

Amide **A6** homodimer

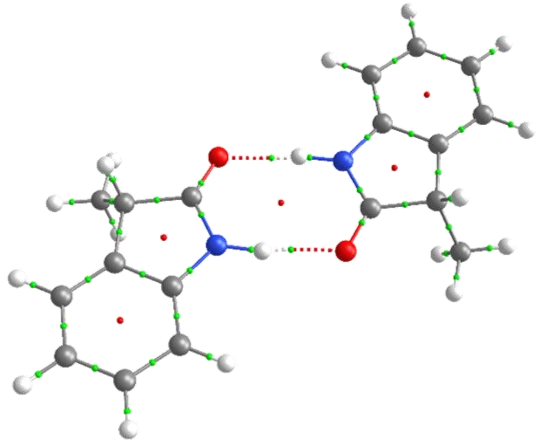

Amide **A7** homodimer

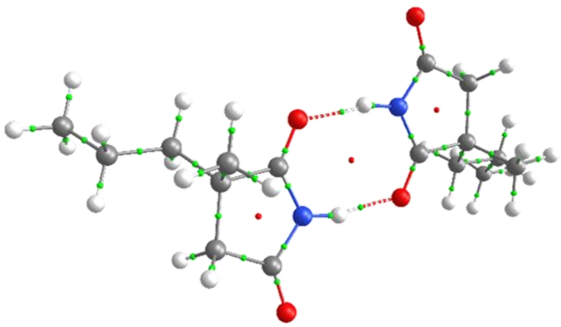

Imide **I2** homodimer

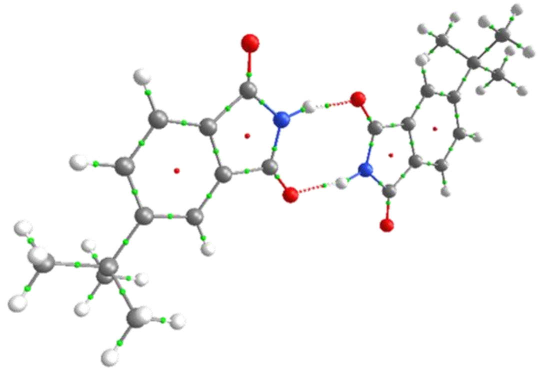

Imide **I3** homodimer

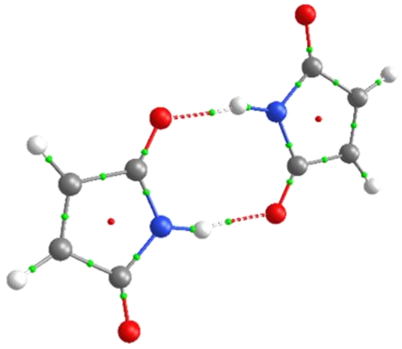

Imide **I4** homodimer

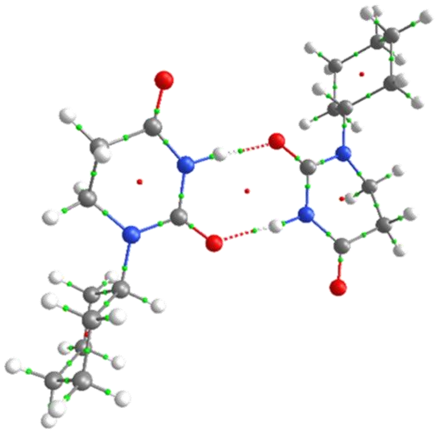

Imide **I5** homodimer

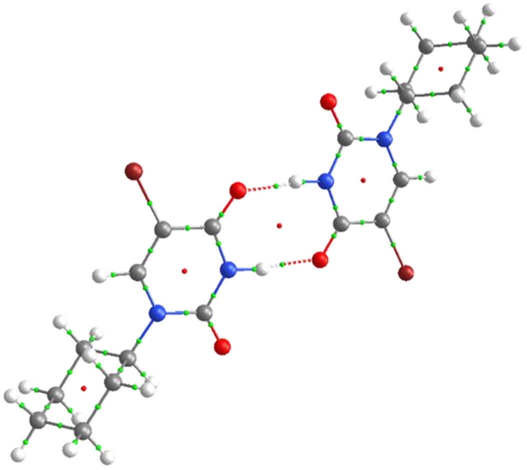

Imide **I6** homodimer

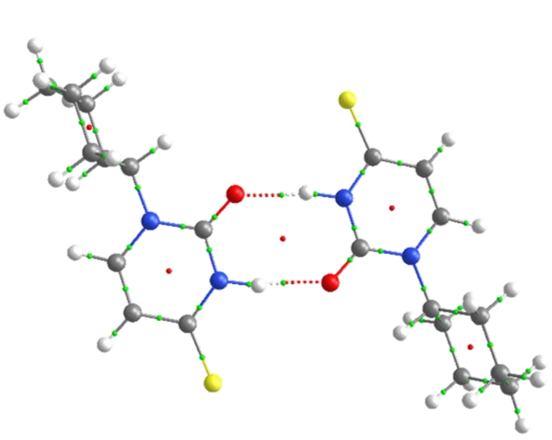

Imide **I7** homodimer

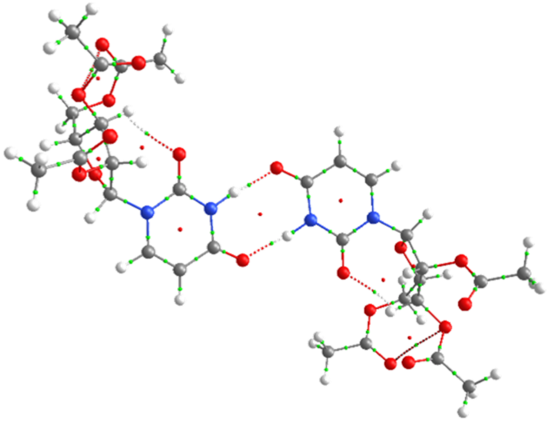

Imide **I8** homodimer

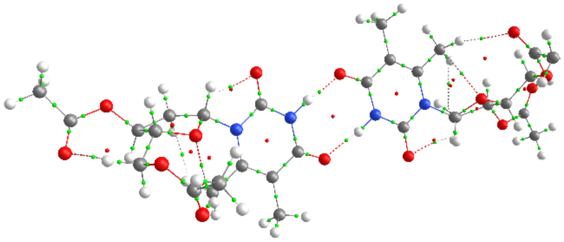

Imide **I9** homodimer

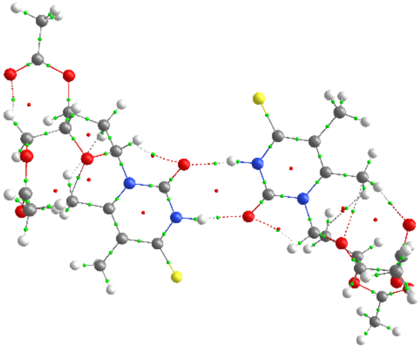

Imide **I10** homodimer

2.1.2 Heterodimers

Table S2. QTAIM molecular graphs of the amide and imide heterodimers studied in this work.

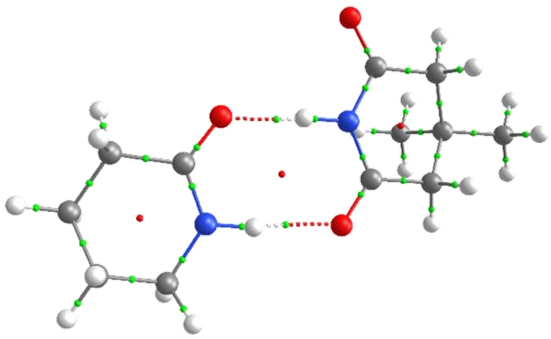	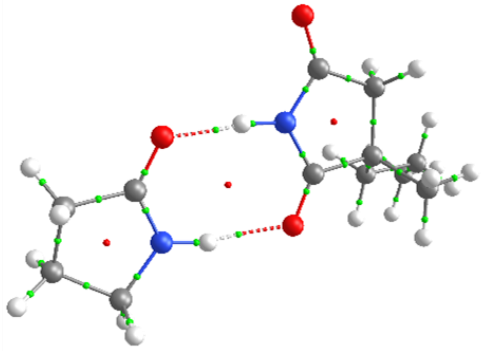
**A5-I1** heterodimer	**A1-I2** heterodimer

2.2 Hydrogen bond formation energies by Espinosa's empirical formula

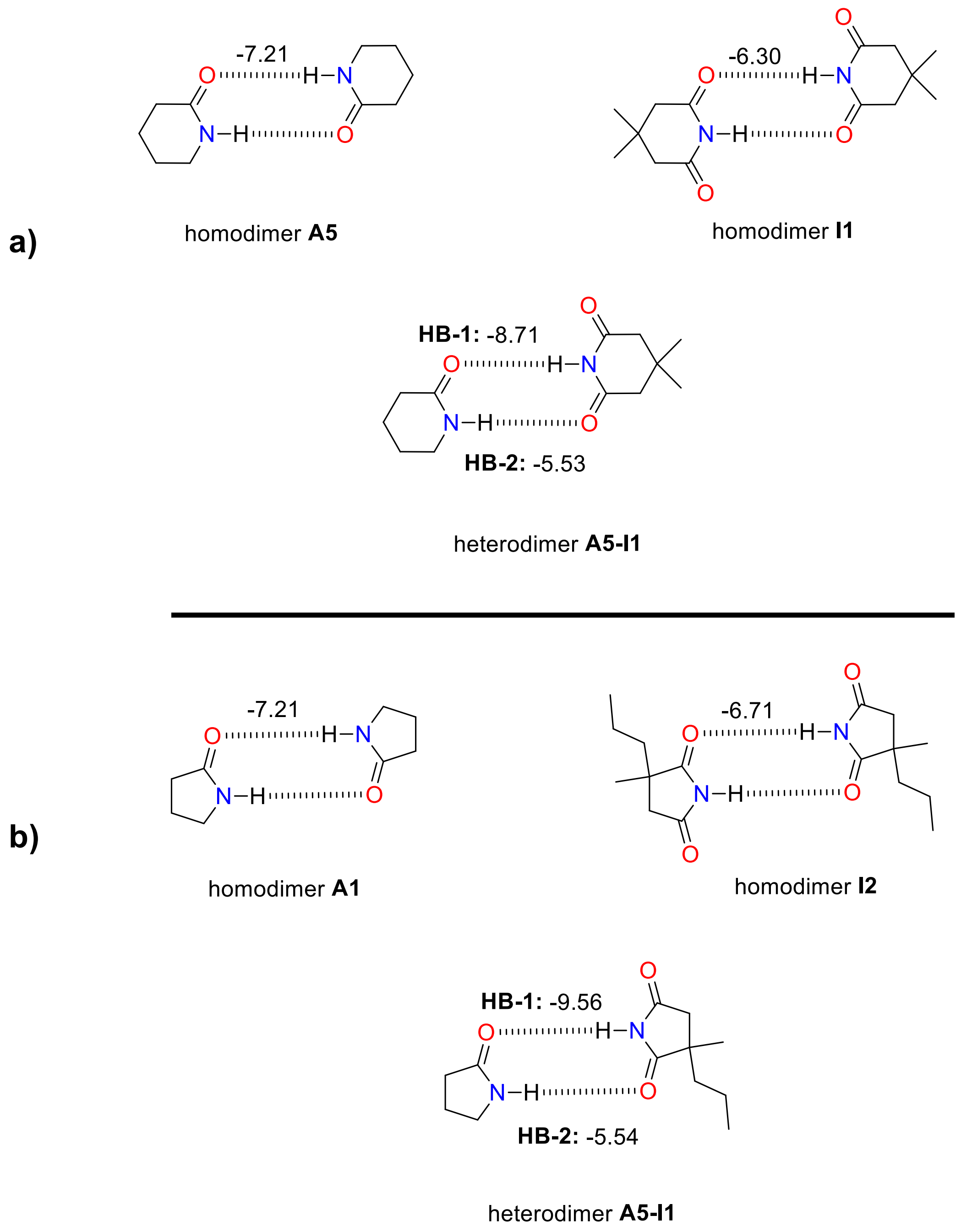

Figure S28. Hydrogen bond formation energies calculated with Espinosa's empirical formula[17] for HBs involved in homo- and heterodimers studied in this work a) **A5** and **I1** and b) **A1** and **I2**. The Espinosa's equation is $E_{HB} = 0.5 \cdot V(\mathbf{r})$ where $V(\mathbf{r})$ is the potential energy density at the bond critical point of the examined HB. All values are given in kcal/mol.

2.3 Use of the interacting quantum atoms approach for the study of bimolecular clusters

The Interacting Quantum Atoms (IQA) method is an electronic energy partition, E, in one (E_{net}) and two-atoms (E_{int}) terms[18]

$$E = \sum_{A} E_{net}^{A} + \sum_{A} \sum_{B>A} E_{int}^{AB}, \qquad (1)$$

wherein the sums run over atomic regions which divide exhaustively the three-dimensional space. The terms E_{net}^{A} and E_{int}^{AB} are referred as (i) the IQA net energy of atom A and (ii) the IQA interaction energy of the pair of atoms A and B respectively.[19] The IQA interaction energy, E_{int}^{AB} can be further split in classical (coulombic) and exchange-correlation contributions,

$$E_{int}^{AB} = E_{cl}^{AB} + V_{XC}^{AB}. \qquad (2)$$

The components E_{cl}^{AB} and V_{XC}^{AB} are related to the covalency and ionicity of the interaction between atoms A and B respectively.

The IQA method is entirely based on the first order reduced density matrix $\rho_1(\boldsymbol{r_1}, \boldsymbol{r_1'})$ and the pair density $\rho_2(\boldsymbol{r_1}, \boldsymbol{r_2})$. Neither of these scalar fields are defined in conventional density functional theory. It is possible, nevertheless, to scale one and two atom terms of the Kohn-Sham exchange-correlation energy in a similar fashion to QTAIM.[20] This procedure allows us to obtain the total DFT electronic energy in accordance with equation (1).

The IQA approach has been successfully used to study molecular clusters.[21] Because the formalism of IQA is invariant with respect to the grouping of several QTAIM basins in functional groups or molecules, the electronic energy of a bimolecular cluster $G \cdots H$ can be written as

$$E^{G\cdots H} = E_{net}^{G} + E_{net}^{H} + E_{int}^{G,H}, \qquad (3)$$

in which E_{net}^{G} comprises the net energies of the atoms within G, along with their corresponding interactions

$$E_{net}^{G} = \sum_{A \in G} E_{net}^{A} + \sum_{A \in G} \sum_{\substack{B \in G \\ B > A}} E_{int}^{AB}. \qquad (4)$$

A similar definition holds for E_{net}^{H} while the quantity $E_{int}^{G,H}$ is defined as

$$E_{int}^{G,H} = \sum_{A \in G} \sum_{B \in H} E_{int}^{AB}. \qquad (5)$$

The change in energy associated with the formation of the molecular cluster $G \cdots H$, ΔE, can be written as the sum of the IQA deformation energies of the monomers and its corresponding interaction defined in equation (5),

$$\begin{aligned} \Delta E &= E^{G \cdots H} - \left(E_{iso}^{G} + E_{iso}^{H}\right) \\ &= E_{def}^{G} + E_{def}^{H} + E_{int}^{G,H} \qquad (6) \end{aligned}$$

The deformation energy of monomer I is defined as

$$E_{def}^{I} = E_{net}^{I} - E_{iso}^{I} \qquad (7)$$

E_{def}^{I} is associated with the changes of the electron density and the nuclear geometry associated with the interaction of monomer I with other species. Finally, we indicate that the analysis presented in this work was performed at the DFT electron density computed with gas-phase single-point calculations at M06-2x/6-311++G(2d,2p) level of theory.

2.3.1 IQA analysis of I1-A5 and I2-A1 heterodimers as well as I1 and I2 homodimers

Table S3. Complete set of intermolecular E_{int}(IQA) values within the I1–I1 homodimer.[‡] The data are reported in kcal/mol.

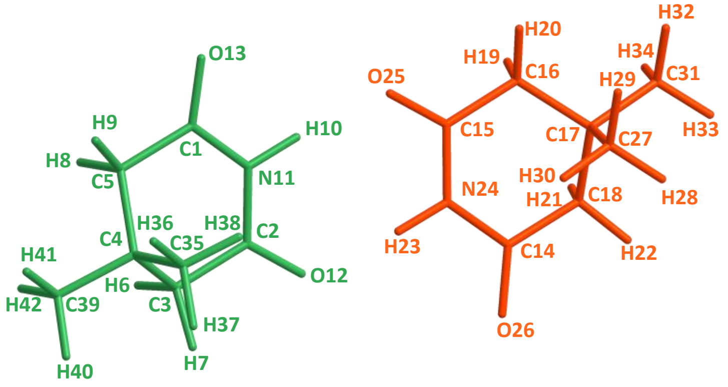

O_s of I1	Atoms of I1	E_{int}	Atoms of I1	E_{int} (with all atoms of I1)
	C14	-79.2	C1	-12.5
	C15	-113.9	C2	4.0
	C16	-2.2	C3	-0.1
	C17	-4.0	C4	-0.2
	C18	-1.4	C5	-0.3
	H19	-1.2	H6	0.0
	H20	-2.0	H7	0.0
	H21	-0.8	H8	-0.1
	H22	-1.6	H9	-0.1
	H23	-37.6	H10	-22.3
O13	N24	81.7	N11	5.1
	O25	114.0	O12	-30.9
	O26	60.6	O13	10.7
	C27	-2.3	C35	-0.1
	H28	0.1	H36	0.0
	H29	0.6	H37	0.0
	H30	0.9	H38	0.0
	C31	-2.6	C39	-0.1
	H32	0.6	H40	0.0
	H33	0.2	H41	0.0
	H34	0.7	H42	0.0
	**Total**	**10.7**	**Total**	**−46.8**

[‡]The IQA deformation energies of the interacting monomers are 16.4 and 20.0 kcal/mol, so that the IQA formation energy of the molecular cluster is E_{form} = (16.4 + 20.0 – 46.8) kcal/mol = −10.4 kcal/mol.

Table S4. Complete set of intermolecular E_{int}(IQA) values within the **I1**–**A5** heterodimer.‡ The data are reported in kcal/mol.

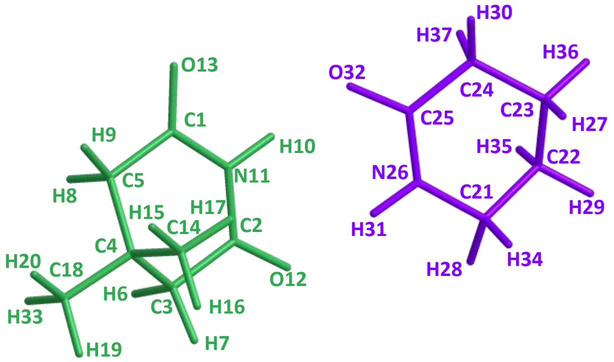

O_s of **I1**	Atoms of **A5**	E_{int}	Atoms of **I1**	E_{int} (with all atoms of **A5**)	Atoms of **A5**	E_{int} (with all atoms of **I1**)
	**C21**	-23.2	**C1**	-19.9	**C21**	-3.2
	**C22**	-3.7	**C2**	0.7	**C22**	-0.4
	**C23**	-3.8	**C3**	-0.1	**C23**	-0.2
	**C24**	-2.8	**C4**	-0.2	**C24**	-0.1
	**C25**	-118.4	**C5**	-0.4	**C25**	6.6
	**N26**	83.1	**H6**	0.0	**N26**	3.5
	**H27**	1.1	**H7**	-0.1	**H27**	0.1
**O13**	**H28**	-0.4	**H8**	-0.1	**H28**	0.0
	**H29**	0.2	**H9**	-0.2	**H29**	0.0
	**H30**	-1.7	**H10**	-32.9	**H30**	0.0
	**H31**	-35.5	**N11**	11.1	**H31**	-19.7
	**O32**	121.4	**O12**	-28.6	**O32**	-40.7
	**H34**	0.4	**O13**	16.9	**H34**	0.0
	**H35**	0.8	**C14**	-0.2	**H35**	0.0
	**H36**	0.4	**H15**	0.0	**H36**	0.0
	**H37**	-1.0	**H16**	-0.1	**H37**	0.0
			**H17**	0.1		
			**C18**	-0.1		
			**H19**	-0.1		
			**H20**	0.0		
			**H33**	0.0		
	**Total**	**16.9**	**Total**	**–54.0**	**Total**	**–54.0**

‡The IQA deformation energies of the amide and imide are 20.9 and 21.7 kcal/mol, so that the IQA formation energy of the molecular cluster is E_{form} = (20.9 + 21.7 – 54.0) kcal/mol = –11.4 kcal/mol.

Table S5. Complete E_{int}(IQA) values within the I2–I2 homodimer.‡ The data are reported in kcal/mol.

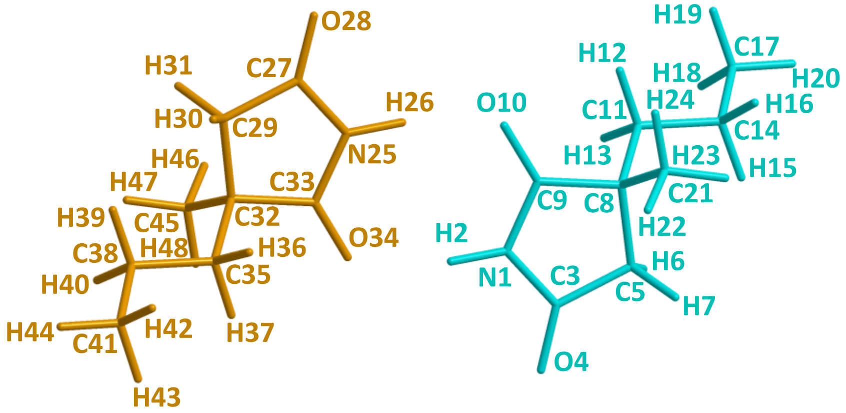

Os of I2	Atoms of I2	E_{int}	Atoms of I2	E_{int} (with all atoms of I2)
	N1	80.7	N25	2.2
	H2	-37.3	H26	-22.8
	C3	-78.8	C27	-9.4
	O4	58.9	O28	8.0
	C5	-0.6	C29	-0.2
	H6	-1.6	H30	-0.1
	H7	-1.7	H31	-0.1
	C8	-2.3	C32	-0.1
	C9	-110.1	C33	9.5
	O10	108.2	O34	-37.6
	C11	-3.7	C35	0.0
	H12	1.4	H36	0.0
O28	H13	0.0	H37	0.0
	C14	-3.7	C38	0.0
	H15	1.2	H39	0.0
	H16	1.1	H40	0.0
	C17	-2.8	C41	0.0
	H18	0.9	H42	0.0
	H19	0.9	H43	0.0
	H20	0.3	H44	0.0
	C21	-3.1	C45	0.0
	H22	80.7	H46	0.1
	H23	-37.3	H47	0.0
	H24	-78.8	H48	0.0
	**Total**	**8.0**	**Total**	**−50.4**

‡The IQA deformation energies of the interacting monomers are 20.5 and 19.0 kcal/mol, so that the IQA formation energy of the molecular cluster is E_{form} = (20.5 + 19.0 – 50.4) kcal/mol = −10.9 kcal/mol.

Table S6. Complete set of intermolecular E_{int}(IQA) values within the **I2**–**A1** heterodimer.[‡] The data are reported in kcal/mol.

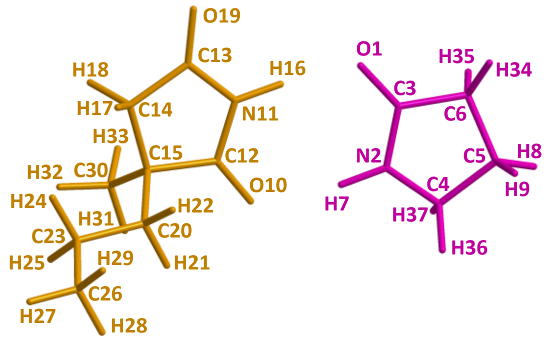

O_s of **I2**	Atoms of **A1**	E_{int}	Atoms of **I2**	E_{int} (with all atoms of **A5**)	Atoms of **A5**	E_{int} (with all atoms of **I2**)
	**O1**	114.2	**O10**	-31.8	**O1**	-48.7
	**N2**	81.4	**N11**	8.9	**N2**	0.3
	**C3**	-114.9	**C12**	4.0	**C3**	13.2
	**C4**	-22.7	**C13**	-17.0	**C4**	-2.3
	**C5**	-3.6	**C14**	-0.2	**C5**	-0.2
	**C6**	-2.2	**C15**	-0.2	**C6**	0.0
**O19**	**H7**	-35.5	**H16**	-34.1	**H7**	-19.3
	**H8**	-0.1	**H17**	-0.1	**H8**	0.0
	**H9**	0.3	**H18**	-0.2	**H9**	0.0
	**H34**	-1.6	**O19**	13.9	**H34**	0.0
	**H35**	-1.1	**C20**	0.0	**H35**	0.1
	**H36**	-0.5	**H21**	-0.1	**H36**	0.0
	**H37**	0.2	**H22**	0.1	**H37**	0.0
			**C23**	0.0		
			**H24**	0.1		
			**H25**	0.0		
			**C26**	0.0		
			**H27**	0.0		
			**H28**	-0.1		
			**H29**	0.0		
			**C30**	0.0		
			**H31**	-0.1		
			**H32**	0.0		
			**H33**	0.1		
	**Total**	**13.9**	**Total**	**–56.9**	**Total**	**–56.9**

[‡]The IQA deformation energies of the interacting monomers are 21.9 and 22.9 kcal/mol, so that the IQA formation energy of the molecular cluster is E_{form} = (21.9 + 22.9 – 56.9) kcal/mol = –12.1 kcal/mol.

2.4 Data for the correlation between |*E*(A)| vs pK_a and |*E*(B)| vs pK_{BH+}

Table S7. Values of |*E*(A)| computed at the (SMD-DMSO)-M06-2x//6-311++G(2d,2p) level of theory and reported experimental pK_a in DMSO for the complete set of studied compounds.

Compound	|*E*(A)| kcal/mol	pK_a in DMSO	Ref.
2-Pyrrolidone (**A1**)	75.0	24.2	22
2-Oxazolidinone (**A2**)	69.8	20.8	22
2-Piperidone (**A5**)	78.8	26.6	22
1,3-Dihydroindol-2-one (**A6**)	64.7	18.5	22
Formamide	72.1	23.5	23
Succinimide	60.9	14.7	24
2-Oxazolone	59.6	15.0	24
Urea	74.7	26.9	25
Thiourea	65.7	21.1	25
N,*N*'-Diphenylurea	64.5	18.7	25
N,*N*'-Diphenylthiourea	58.2	13.4	26
N'-Phenyl-*N*-[3-(trifluoromethyl)phenyl]thiourea	56.3	12.1	26
N-[3,5-Bis(trifluoromethyl)phenyl]-*N*'-phenylurea	59.8	16.1	26
N-[3,5-Bis(trifluoromethyl)phenyl]-*N*'-phenylthiourea	54.6	10.7	26
N,*N*'-Bis[3-(trifluoromethyl)phenyl]thiourea	54.9	10.9	26
N,*N*'-Bis[3,5-bis(trifluoromethyl)phenyl]urea	57.9	13.8	26
N,*N*'-Bis[3,5-bis(trifluoromethyl)phenyl]thiourea	52.2	8.5	26
Acetic acid	58.9	12.6	27
3,4-Bis(phenylamino)-3-cyclobutene-1,2-dione (*N*,*N*'-Diphenylsquaramide)	55.8	12.5	28
N'-Phenyl-*N*-[3-(trifluoromethyl)phenyl]squaramide	52.5	10.6	28

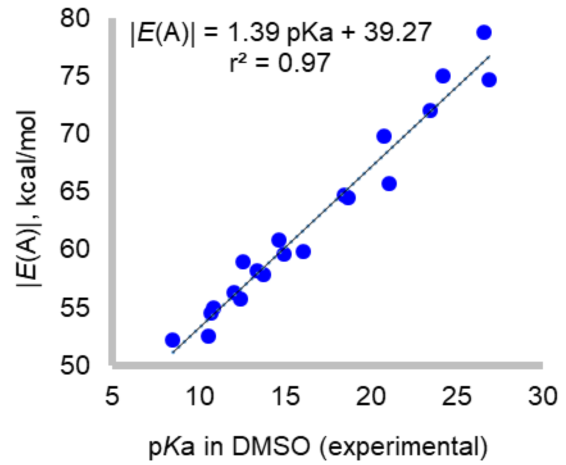

Figure S29. Correlation of experimental pK_a with |*E*(A)| for the compounds indicated in Table S7.

Table S8. Computed values of |*E*(B)| at the (SMD-water)-M06-2x//6-311++G(2d,2p) level of theory and reported experimental pK_{BH+} in H_2O for the set of compounds considered in this work.

Compound	|*E*(B)| kcal/mol	pK_{BH+} in water	Ref.
2-Pyrrolidone (**A1**)	13.4	-0.65	29
2-Piperidone (**A5**)	14.9	-0.18	29
ε-Caprolactam (**A3**)	14.5	-0.46	29
2-Azacyclooctanone	14.6	-0.38	29
N-Methyl-2-pyrrolidone	12.8	-0.75	29
Acetamide	12.5	-0.73	29
Propanamide	12.2	-0.86	30
2-Methylpropanamide	12.0	-1.11	30
t-Butylformamide	9.6	-1.49	30
N-Methylformamide	10.4	-1.10	30
N-Methylacetamide	14.5	-0.56	30
N-Methylpropanamide	12.4	-0.70	30
2-Cloroacetamide	6.7	-2.80	31
4-Methylbenzamide	11.0	-1.44	32
4-Nitrobenzamide	7.8	-2.28	32
4-Chlorobenzamide	9.7	-1.66	32
N-(2,2,2-Trifluoroethyl)benzamide	5.6	-3.33	33
2-Fluorobenzamide	8.7	-1.98	34

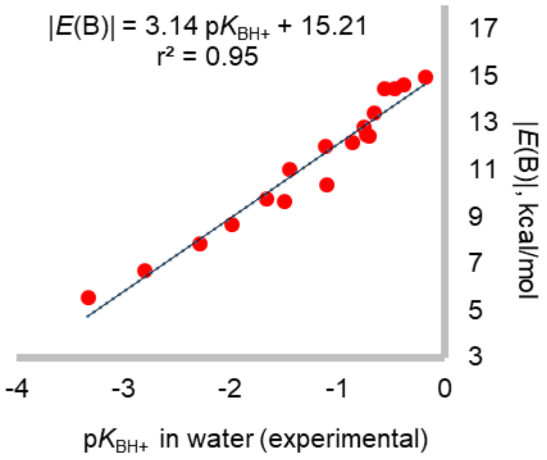

Figure S30. Correlation of experimental pK_{BH+} with |E(B)| for the species indicated in Table S8.

2.5 Hydrogen bonds between A2 and a chloroform molecule

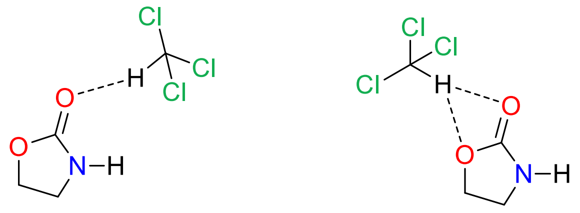

Cx-1 E = -3.67 kcal/mol

Cx-2 E = -5.02 kcal/mol

ΔE = 1.35 kcal/mol

Figure S31. Hydrogen bonds between 2-oxazolidonone (**A2**) and a chloroform molecule. **Left:** H-bond formed with the carbonyl oxygen. **Right:** Bifurcated HBs which involve the two oxygen atoms of **A2**.

2.6 Correlation of experimental and that computed with the first-degree model herein

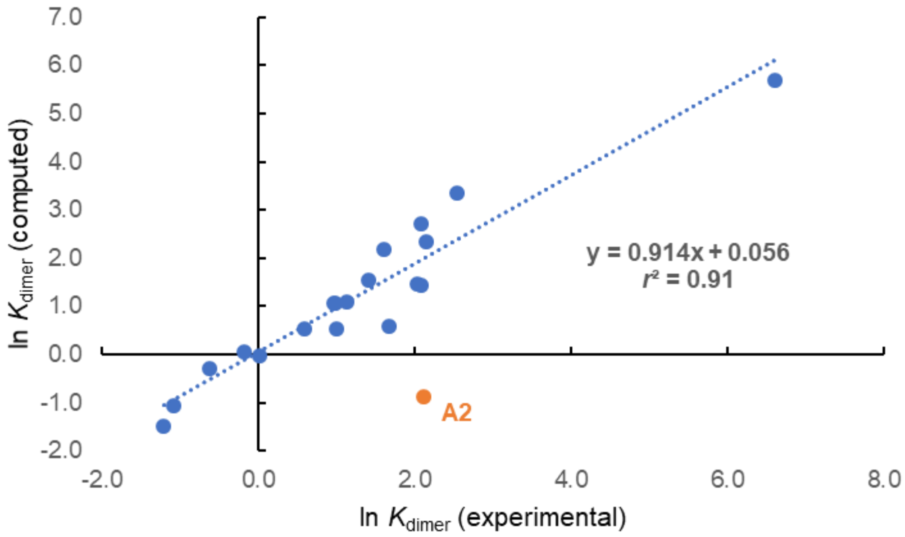

Figure S32. Correlation of experimental ln K_{dimer} with its computed counterpart estimated with the equation indicated in Figure 5 of the paper for all species studied in this work.

2.7 Acidity and basicity values of the investigated systems in CCl_4

Table S9. ln K_{dimer} in CCl_4 determined by infrared spectroscopy.

Comp.	ln K_{dimer}	$\|E(A)\|$	$\|E(B)\|$
		kcal/mol	
**A1**	5.11[35]	130.0	35.9
**A3**	4.53[35]	134.7	38.7
**A5**	4.88[35]	134.0	39.3
**A8**	3.93[35]	126.5	30.1
**A9**	4.34[35]	135.2	40.3
**I14**	2.69[b,36]	112.8	23.5
**I15**	1.98[b,36]	98.9	9.0
**I16**	0.96[b,36]	107.0	13.0
**I17**	0.10[b,36]	88.9	-3.4
**I18**	4.11[36]	117.0	32.6

[a]The $|E(B)|$ and $|E(A)|$ values were calculated with the SMD-(CCl_4)-M06-2x/6-311++G(2d,2p) method.
[b]Statistical factor applied.

2.8 Delocalisation indices

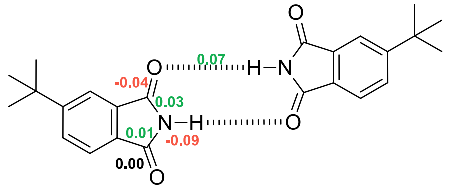

I3 homodimer

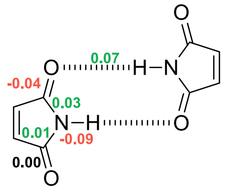

I4 homodimer

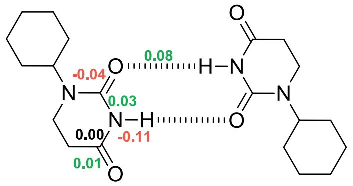

I5 homodimer

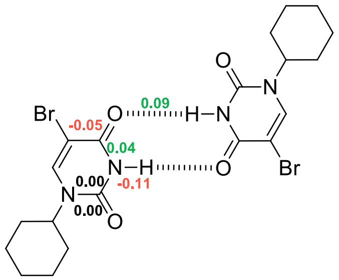

I6 homodimer

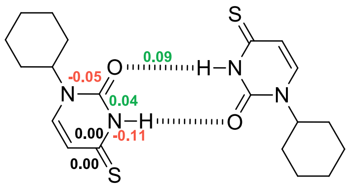

I7 homodimer

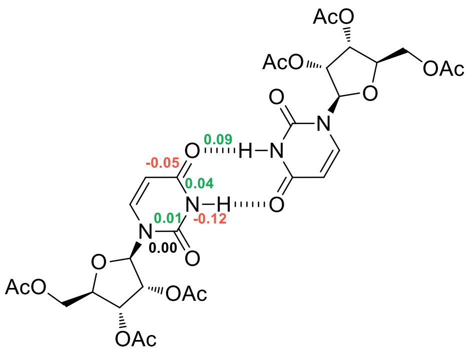

I8 homodimer

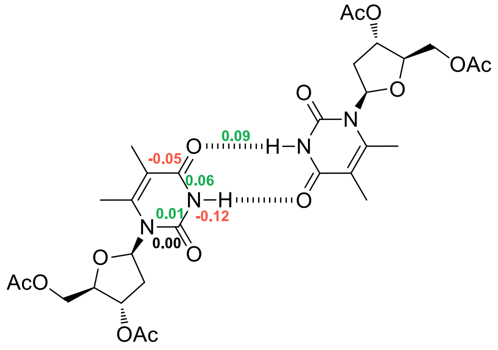

I9 homodimer

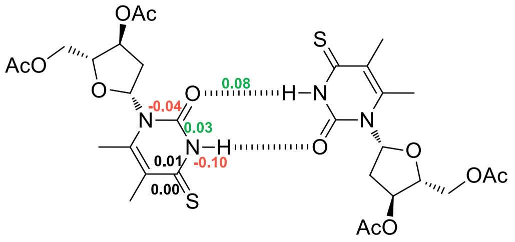

I10 homodimer

Figure S33. Changes in the electron delocalisation indices as a consequence of the dimerisation of the imides studied in this work. The corresponding data for **I1**and **I2** are reported in Figure 3 in the body of the paper. The DIs whose change is negative (in red) indicate interactions with a decreased covalent character due to the formation of the molecular cluster. A positive value for a change in a DI (in green) evidences an increased covalent bond character as a result of the formation of the HB.

2.9 Bond lengths

Table S10. Changes in bond lengths ($l_{dimer} - l_{monomer}$) involved in the resonance-assisted hydrogen bond within the imide homodimers examined in this study, i. e. N-H, $(C=O)_{HB}$ and $(N\text{-}C)_{HB}$, and the spectator chemical bonds $(N\text{-}C)_S$ and $(C=O,S)_S$. The data are reported in angstroms.

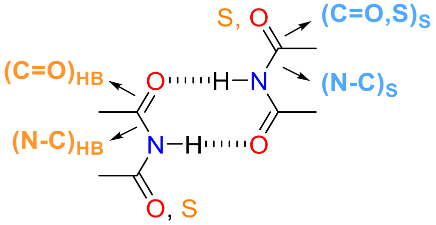

Compound	$(C=O)_{HB}$	$(N\text{-}C)_{HB}$	N-H	$(N\text{-}C)_S$	$(C=O,S)_S$
**I1**	0.008	-0.011	0.010	0.003	-0.001
**I2**	0.009	-0.012	0.011	0.004	-0.001
**I3**	0.008	-0.011	0.010	0.003	-0.001
**I4**	0.008	-0.010	0.010	0.003	-0.001
**I5**	0.009	-0.011	0.013	0.000	0.000
**I6**	0.010	-0.012	0.014	0.000	-0.001
**I7***	0.009	-0.008	0.011	0.000	0.000
**I8**	0.011	-0.013	0.015	0.000	0.000
**I9**	0.010	-0.013	0.014	0.001	-0.003
**I10***	0.009	-0.009	0.011	0.000	0.000

*Compounds with **S**.

2.10 XYZ coordinates and electronic energies

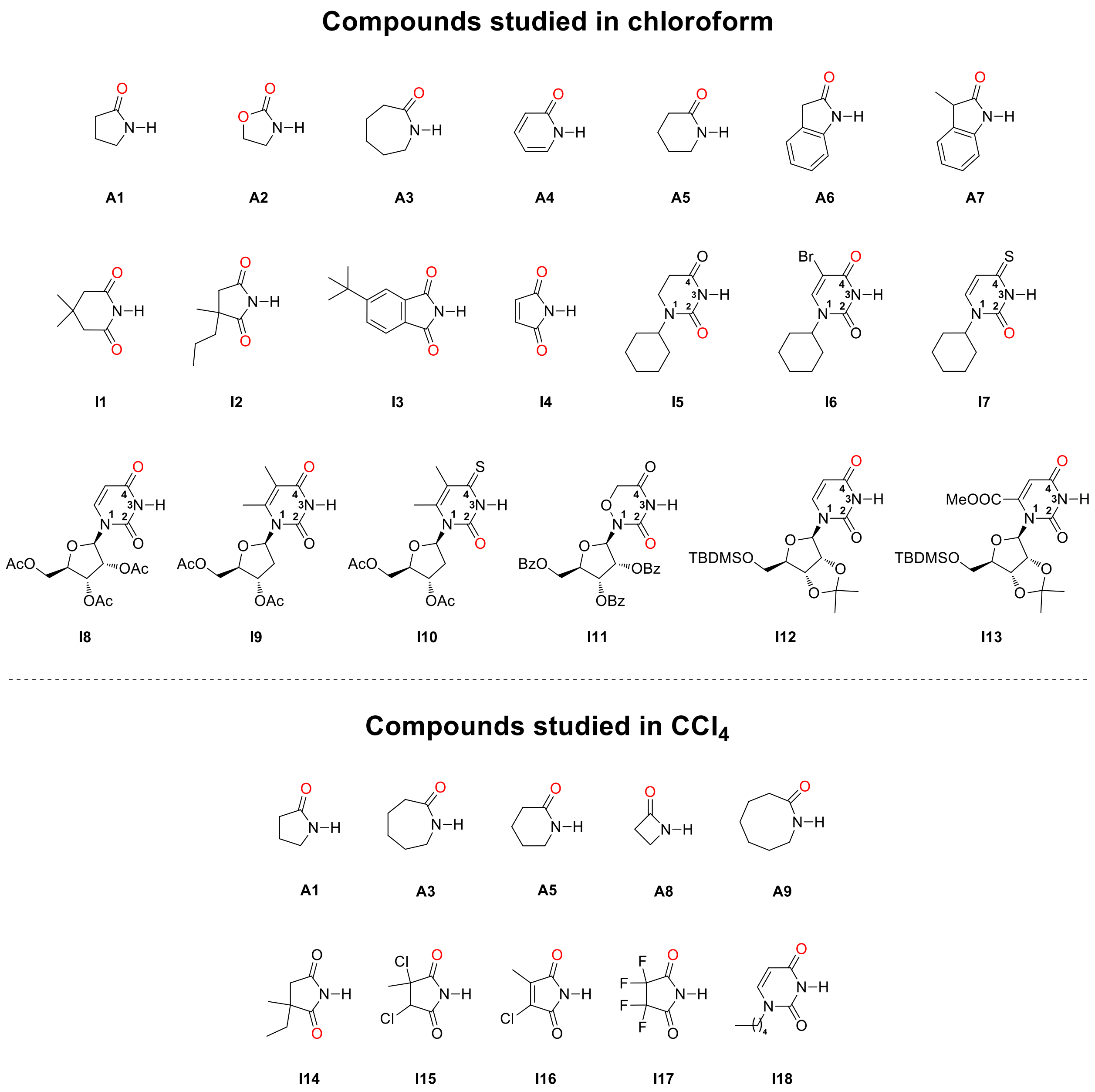

Figure S34. Structure of the compounds considered in this work. The oxygen atoms displayed in red are the most basic within the molecule and therefore, a proton is added to this atom to obtain the corresponding protonated species.

2.10.1 Compounds studied in $CDCl_3$

A1

Electronic energy (E_e)	E_e + ZPV
Hartree	
-286.613588	-286.501756

XYZ coordinates

C	-1.32088800	-0.80804200	0.13764800
C	0.89035900	-0.00002600	-0.00773100
C	-0.00870500	1.21439600	0.14718200
C	-1.40379000	0.68867500	-0.19928200
H	0.06160000	1.53332300	1.18958300
H	0.33803500	2.03122100	-0.47994000
H	-2.20300000	1.18636700	0.34303200
H	-1.58648300	0.80660300	-1.26667200
H	0.48498900	-2.01724300	-0.02466100
N	0.09127600	-1.09058400	-0.07646300
O	2.10882900	-0.00692100	-0.04464700
H	-1.60234900	-1.00175000	1.17536200
H	-1.94421600	-1.41908600	-0.51119600

A1 (protonated)

Electronic energy (E_e)	E_e + ZPV
Hartree	
-287.0332448	-286.908137

XYZ coordinates

C	-1.36178500	-0.84342300	0.10484700
C	0.77592500	0.02179400	0.00196900
C	-0.08710400	1.22397400	0.13514300
C	-1.48182700	0.66113200	-0.17934500
H	0.01132800	1.57548200	1.16547100
H	0.24776800	2.01948100	-0.52558600
H	-2.26105600	1.12208100	0.41785300
H	-1.70986700	0.81759700	-1.23098600
H	0.50381000	-1.99811200	-0.05687600
N	0.08989600	-1.07206200	-0.02946500
O	2.06788500	0.11397000	-0.04454100
H	-1.65827800	-1.11148300	1.11713500
H	-1.89359500	-1.46571900	-0.60745800
H	2.51627900	-0.74751500	-0.09265900

A1 (deprotonated)

Electronic energy (E_e)	E_e + ZPV
Hartree	
-286.091891	-285.994036

XYZ coordinates

C	-1.28583600	-0.82130100	0.11541600

C	0.85932600	-0.10646800	-0.00618700
C	0.00759300	1.16936500	0.16030900
C	-1.39079600	0.68521600	-0.20016000
H	0.07620400	1.49180500	1.20356800
H	0.38892400	1.97675900	-0.46308500
H	-2.19711700	1.18224400	0.33998100
H	-1.56556300	0.82168300	-1.26910300
N	0.11447700	-1.19159200	-0.04938700
O	2.11341100	-0.03251200	-0.06165600
H	-1.62084000	-1.01408400	1.14525700
H	-1.93195900	-1.41804800	-0.53392800

A1 homodimer

Electronic energy (E_e)	E_e + ZPV
Hartree	
-573.241549	-573.016034

XYZ coordinates

O	1.00376500	1.60281000	-0.14655100
N	1.71330700	-0.57212500	-0.04824700
C	1.89132000	0.75659700	-0.04088700
C	2.93146300	-1.32947700	0.20652900
C	4.03243400	-0.31219300	-0.13065300
C	3.36904100	1.03904300	0.14678200
H	0.77896400	-0.98549300	-0.05474400
H	2.97246200	-2.21854600	-0.41827800
H	2.97726200	-1.63934400	1.25296800
H	4.28157900	-0.38881300	-1.18843600

H	4.93666300	-0.47991000	0.44809300
H	3.69184500	1.84740000	-0.50404000
H	3.50736500	1.36127000	1.18134700
O	-1.00398100	-1.60930300	-0.08425200
N	-1.70759900	0.56727000	0.01388600
C	-1.89314000	-0.75856200	-0.05770000
C	-2.93946900	1.34029000	-0.06785400
C	-4.01108000	0.30117100	0.29757500
C	-3.38324300	-1.02866100	-0.12701400
H	-0.77530600	0.97945200	-0.05402700
H	-2.91548000	2.18005700	0.62279100
H	-3.08160800	1.72789700	-1.07894100
H	-4.16677500	0.30692500	1.37563500
H	-4.96152300	0.50564800	-0.18747800
H	-3.65083100	-1.87848400	0.49535700
H	-3.61679500	-1.28136800	-1.16395300

A2

Electronic energy (E_e)	E_e + ZPV
Hartree	
-322.531985	-322.44332

XYZ coordinates

C	-1.34147900	0.73468200	-0.14961900
C	0.84874600	-0.00205000	0.01259500
C	-1.29150100	-0.76968100	0.11244600
H	0.42038300	1.98474500	-0.02959500
N	0.03299300	1.07820300	0.17885000

O	2.05208500	-0.02477500	-0.03182400
H	-1.89677400	-1.35354200	-0.57310600
H	-2.05263200	1.23701400	0.50007800
H	-1.57386200	0.95961000	-1.19158900
H	-1.55561800	-1.00674500	1.14229000
O	0.08953400	-1.11850100	-0.08724600

A2 (protonated)

Electronic energy (E_e)	E_e + ZPV
Hartree	
-322.9364176	-322.834595

XYZ coordinates

C	-1.40752600	0.76169600	-0.05415000
C	0.73348200	-0.01780200	-0.00043900
C	-1.37087400	-0.77138200	0.05682700
H	0.41556900	2.00360400	0.05122900
N	0.02338200	1.07125000	0.04456100
O	2.01743600	-0.13320500	-0.00046500
H	-1.88273800	-1.27997800	-0.75090800
H	-1.95179200	1.22344100	0.76277000
H	-1.79414100	1.10862800	-1.00800600
H	-1.70515600	-1.13775200	1.02143900
O	0.05144000	-1.11391700	-0.05078500
H	2.47308200	0.72520700	0.00811400

A2 (deprotonated)

Electronic energy (E_e)	E_e + ZPV
Hartree	
-322.01994	-321.945403

XYZ coordinates

C	1.31295900	-0.75741300	0.09653000
C	-0.82930100	-0.11247500	-0.00456700
C	1.27157700	0.75951300	-0.12152700
N	-0.06783800	-1.17768300	-0.08427300
O	-2.05898700	0.00893000	-0.01534200
H	1.91322500	1.32469500	0.55418200
O	-0.08508200	1.09183100	0.12440600
H	1.67171700	-1.00077500	1.10530100
H	1.98704000	-1.24546900	-0.61041600
H	1.52402300	1.02149400	-1.15427600

A2 homodimer

Electronic energy (E_e)	E_e + ZPV
Hartree	
-645.078177	-644.899207

XYZ coordinates

C	-2.95896600	-1.29990600	0.06281400
C	-1.86199100	0.72380100	-0.10434500
C	-3.92783000	-0.13555900	0.26882200
H	-4.19643300	-0.01542700	1.31739600
H	-4.82209000	-0.19380300	-0.34228300
H	-0.78913800	-1.01691400	-0.01675300

N	-1.69581000	-0.58791700	0.16032700
O	-1.00536200	1.56672200	-0.28910400
C	2.95898700	1.29991100	0.06294400
C	1.86199400	-0.72382200	-0.10401100
C	3.92780800	0.13556000	0.26917700
H	4.19630900	0.01553400	1.31778700
H	4.82213000	0.19372900	-0.34184600
H	0.78917000	1.01692100	-0.01694000
N	1.69580800	0.58795400	0.16038300
O	1.00536200	-1.56672500	-0.28883100
H	-3.07810200	-1.76485900	-0.91669600
H	-3.06063200	-2.05514100	0.83718800
H	3.06057900	2.05520900	0.83726900
H	3.07825500	1.76478100	-0.91658800
O	-3.17719400	1.02641700	-0.13222600
O	3.17718800	-1.02643800	-0.13182700

A3

Electronic energy (E_e)	E_e + ZPV
Hartree	
-365.2280745	-365.058037

XYZ coordinates

C	-1.37783700	0.03330000	0.02834700
C	-0.61799300	-1.14156700	0.60276500
C	0.58463400	-1.55759800	-0.25892200
C	1.84149600	-0.71977800	-0.03231300
C	0.63196500	1.48837800	0.41885400

C	1.70518400	0.75872000	-0.38494300
H	-0.28013400	-0.90176500	1.61487900
H	0.29882400	-1.52908000	-1.31400300
H	2.12896800	-0.80224000	1.02074200
H	0.73453800	1.24783500	1.48029200
H	-1.33531800	-1.95527200	0.67203300
H	0.81840500	-2.59748500	-0.02821400
H	2.66000500	-1.14905400	-0.61302400
H	0.77049100	2.56322900	0.32374700
H	2.66047000	1.25305000	-0.19655500
H	1.48628300	0.87738400	-1.44924600
N	-0.73097400	1.21864700	-0.02027900
O	-2.52244000	-0.08469700	-0.39585100
H	-1.25088300	1.97171700	-0.44461900

A3 (protonated)

Electronic energy (E_e)	E_e + ZPV
Hartree	
-365.649532	-365.465876

XYZ coordinates

C	1.26424100	-0.03231300	0.07659900
C	0.55333600	1.15641200	0.61218700
C	-0.64356100	1.55599800	-0.27195000
C	-1.88750000	0.70163900	-0.04900800
C	-0.67013800	-1.49987100	0.42699600
C	-1.72859200	-0.77649600	-0.39462200
H	0.20825200	0.92337500	1.62304900

H	-0.34361300	1.53411400	-1.32193800
H	-2.18768500	0.78816500	0.99905500
H	-0.74920800	-1.24980400	1.48539300
H	1.28098100	1.96099600	0.67401100
H	-0.87747100	2.59238800	-0.03386200
H	-2.70267900	1.11426000	-0.64367600
H	-0.77223700	-2.57620700	0.32735600
H	-2.67522500	-1.28474300	-0.20819600
H	-1.50204700	-0.90413800	-1.45565300
N	0.70774500	-1.19677000	-0.01020600
O	2.48533500	0.17771000	-0.33309500
H	1.23563600	-1.96153500	-0.41892700
H	2.92168500	-0.61338300	-0.69162300

A3 (deprotonated)

Electronic energy (E_e)	E_e + ZPV
Hartree	
-364.698904	-364.542681

XYZ coordinates

C	1.35488200	-0.15332800	0.00837500
C	0.62736900	1.04805500	0.63676800
C	-0.54067600	1.54997300	-0.22177600
C	-1.81884100	0.72997100	-0.05560600
C	-0.58627200	-1.47549600	0.41380200
C	-1.67466000	-0.75198800	-0.39356500
H	0.25577400	0.79860300	1.63590400
H	-0.23363600	1.55284300	-1.27216100

H	-2.14701200	0.81604400	0.98620900
H	-0.68692200	-1.17243100	1.46869400
H	1.37030000	1.83530000	0.74869500
H	-0.76184900	2.58760900	0.04105300
H	-2.61195700	1.16923900	-0.66702500
H	-0.81670100	-2.54360800	0.39430200
H	-2.63809100	-1.24070800	-0.21754300
H	-1.44395200	-0.87440000	-1.45594500
N	0.76790000	-1.32516900	-0.08870100
O	2.52099100	0.08307100	-0.42115800

A3 homodimer

Electronic energy (E_e)	E_e + ZPV
Hartree	
-730.4706377	-730.12775

XYZ coordinates

C	3.31693400	1.38223600	-0.50704000
C	3.96625300	1.10256800	0.85805300
C	4.56625000	-0.29606300	0.99249000
C	2.83637400	-1.48650800	-0.47170200
C	3.56612300	-1.44339800	0.86875500
H	3.93947600	0.96293900	-1.30216800
H	3.22942000	1.27209200	1.64781400
H	5.33601100	-0.41930100	0.22396500
H	3.55377400	-1.37151200	-1.28884700
H	3.23183000	2.45285100	-0.67466000
H	4.75982600	1.83526700	1.00851100

H	5.07364800	-0.37089700	1.95615500
H	2.36210700	-2.45751200	-0.59970000
H	4.09988700	-2.38775500	0.99225700
H	2.82286100	-1.38687700	1.66812200
N	1.76866600	-0.50435600	-0.60052200
O	0.94064300	1.58331100	-0.69231400
H	0.81013700	-0.85373100	-0.64251400
C	-1.91669100	-0.82628300	-0.61505600
C	-3.31684400	-1.38223200	-0.50716400
C	-3.96613800	-1.10271300	0.85797400
C	-4.56626200	0.29585800	0.99253000
C	-2.83654400	1.48655300	-0.47160900
C	-3.56622100	1.44327300	0.86887600
H	-3.93945900	-0.96295200	-1.30224400
H	-3.22925500	-1.27220200	1.64769400
H	-5.33604400	0.41909600	0.22402800
H	-3.55395500	1.37151900	-1.28873300
H	-3.23161600	-2.45282100	-0.67486700
H	-4.75963300	-1.83549800	1.00843000
H	-5.07364300	0.37057400	1.95621200
H	-2.36235400	2.45759400	-0.59958600
H	-4.10002600	2.38759000	0.99250400
H	-2.82292200	1.38670500	1.66820600
N	-1.76874000	0.50449000	-0.60054600
O	-0.94053000	-1.58310900	-0.69237200
H	-0.81025200	0.85395300	-0.64235800

A4

Electronic energy (E_e)	E_e + ZPV
Hartree	
-323.5031549	-323.408836

XYZ coordinates

C	1.05070800	-1.18165300	0.00021400
C	1.79522200	-0.04954300	0.00017300
C	1.11009000	1.19584600	-0.00006100
C	-0.24681200	1.25714800	-0.00017400
C	-1.04968000	0.05742500	-0.00002500
N	-0.30610600	-1.11468700	0.00009700
H	1.68403100	2.11363300	-0.00010700
H	1.47245600	-2.17567000	0.00035100
H	2.87157700	-0.10442500	0.00032200
H	-0.77879700	2.19693500	-0.00035400
H	-0.84406500	-1.97157700	0.00011600
O	-2.27745400	0.00857300	-0.00022100

A4 (protonated)

Electronic energy (E_e)	E_e + ZPV
Hartree	
-323.9258062	-323.818046

XYZ coordinates

C	-1.06956900	-1.20074300	-0.00023600
C	-1.83491100	-0.07332700	-0.00017700
C	-1.19115000	1.17187700	0.00002900

C	0.18102200	1.25873400	0.00017300
C	0.92373100	0.08201200	0.00009700
N	0.28183100	-1.09461900	-0.00009300
H	-1.77804300	2.07985200	0.00007700
H	-1.45977700	-2.20620500	-0.00039200
H	-2.90992900	-0.15506100	-0.00028300
H	0.70808900	2.20018800	0.00034100
O	2.24086000	0.12666700	0.00019500
H	0.82646600	-1.95346000	-0.00012600
H	2.65876400	-0.74764300	0.00015000

A4 (deprotonated)

Electronic energy (E_e)	E_e + ZPV
Hartree	
-323.0016407	-322.921145

XYZ coordinates

C	0.99837500	-1.18636800	0.00023400
C	1.78042800	-0.04349100	0.00016100
C	1.09857600	1.18298000	-0.00006600
C	-0.27087900	1.19854500	-0.00018700
C	-1.03007200	-0.02979500	-0.00012200
N	-0.33356900	-1.21523700	0.00011800
H	1.65079600	2.11634600	-0.00011800
H	1.48587100	-2.15919500	0.00041600
H	2.85863700	-0.10501700	0.00027700
H	-0.82431600	2.12855300	-0.00034100
O	-2.28682300	-0.02565700	-0.00014700

A4 homodimer

Electronic energy (E_e)	E_e + ZPV
Hartree	
-647.0263015	-646.836244

XYZ coordinates

C	-2.77370700	-1.41105700	-0.00021000
C	-1.86694100	0.82437400	-0.00013000
C	-3.21768000	1.30616600	0.00028700
C	-4.26627700	0.43641400	0.00038500
C	-4.05688700	-0.96384400	0.00020000
H	-0.76686900	-0.91416400	-0.00049300
N	-1.73469500	-0.54380000	-0.00033700
C	2.77379500	1.41106900	-0.00019900
C	1.86689100	-0.82425900	-0.00010400
C	3.21763200	-1.30617400	0.00027800
C	4.26627600	-0.43652400	0.00042400
C	4.05692700	0.96379600	0.00010900
H	0.76691500	0.91428800	-0.00015900
N	1.73468400	0.54384200	-0.00022300
O	0.85289300	-1.54903800	-0.00025200
H	-4.87893800	-1.66096200	0.00043600
H	-3.35966200	2.37649900	0.00052100
H	3.35941000	-2.37653500	0.00048800
H	-5.27769100	0.82167000	0.00060900
H	2.50644400	2.45799800	-0.00030500
H	4.87902500	1.66086200	0.00009500
H	5.27767600	-0.82180700	0.00078400
H	-2.50634000	-2.45798600	-0.00040900

O	-0.85290200	1.54904800	-0.00023300

A5

Electronic energy (E_e)	E_e + ZPV
Hartree	
-325.9236342	-325.78234

XYZ coordinates

C	1.04235900	-1.27720500	0.13433600
C	-1.13153500	-0.01409200	-0.01818500
C	-0.37156900	1.29250000	-0.11167000
C	1.08790500	1.20052200	0.32042200
C	1.73554100	-0.01128600	-0.33700600
H	1.30754000	-1.47582900	1.17641800
H	1.35808400	-2.13528900	-0.45756500
H	-0.43446600	1.60033700	-1.15917000
H	-0.93078200	2.02598500	0.46630300
H	1.61216200	2.11769500	0.05417400
H	1.14745500	1.09766100	1.40704600
H	1.65041900	0.07221200	-1.42336000
H	2.79488700	-0.07833100	-0.09119500
H	-0.95868300	-1.99704500	0.10586100
N	-0.40877600	-1.15498600	0.01528800
O	-2.35767400	-0.03564100	-0.01411400

A5 (protonated)

Electronic energy (E_e)	E_e + ZPV
Hartree	
-326.3460087	-326.191241

XYZ coordinates

C	1.06418200	-1.30050400	0.12405400
C	-1.01285700	0.01014300	-0.00993900
C	-0.29400900	1.30874000	-0.07382600
C	1.17605700	1.17988900	0.30766800
C	1.77138900	-0.05134300	-0.36231200
H	1.32485200	-1.52994500	1.15685900
H	1.29555500	-2.16482600	-0.49281000
H	-0.41054400	1.66067800	-1.10345700
H	-0.83919300	2.00936000	0.55814300
H	1.70320000	2.08184300	0.00656500
H	1.26767300	1.09172700	1.39150400
H	1.67058700	0.02931900	-1.44642500
H	2.83058500	-0.15049300	-0.13384100
H	-0.96021800	-1.97390100	0.13051200
N	-0.40326300	-1.12601200	0.07356700
O	-2.31602200	0.09013700	-0.05557900
H	-2.76005300	-0.77432600	-0.05126000

A5 (deprotonated)

Electronic energy (E_e)	E_e + ZPV
Hartree	
-325.3954008	-325.268562

XYZ coordinates

C	0.99887200	-1.26832200	0.14654300
C	-1.10930700	-0.12791200	-0.00419100
C	-0.39083300	1.23395400	-0.05926100
C	1.09155700	1.19769800	0.29137900
C	1.70788300	-0.02224400	-0.37496500
H	1.31629400	-1.42361000	1.18949100
H	1.36474700	-2.14401200	-0.39932700
H	-0.52365700	1.60857600	-1.07870400
H	-0.94472900	1.91315600	0.58979200
H	1.58711200	2.12275200	-0.01148600
H	1.21512900	1.10756300	1.37524200
H	1.57501100	0.05010400	-1.45929000
H	2.78016700	-0.09294900	-0.17691700
N	-0.45490700	-1.26154800	0.06979900
O	-2.37184400	-0.04872400	-0.06430400

A5 homodimer

Electronic energy (E_e)	E_e + ZPV
Hartree	
-651.8616575	-651.577472

XYZ coordinates

C	-2.87015200	-1.50642700	-0.03056400
C	-1.91946500	0.80600300	0.00899100
C	-3.31183000	1.39664500	-0.00806600
C	-4.40604600	0.40841700	-0.39604700
C	-4.20229000	-0.89465100	0.36523500
H	-3.48643000	1.77792900	1.00179900
H	-3.28136600	2.26222200	-0.66788400
H	-5.38503000	0.83847200	-0.18734600
H	-4.36223900	0.20914200	-1.46980000
H	-4.21046900	-0.69669400	1.43999000
H	-4.99666500	-1.60928500	0.15273500
H	-0.83095800	-0.87818700	0.03734600
N	-1.79069500	-0.52539700	0.04147200
O	-0.93204600	1.55286100	0.02373500
C	2.87014500	1.50643000	-0.03056900
C	1.91946400	-0.80600500	0.00883800
C	3.31183400	-1.39664100	-0.00809300
C	4.40606000	-0.40841300	-0.39604000
C	4.20228100	0.89465400	0.36523900
H	3.48638900	-1.77790400	1.00179100
H	3.28140200	-2.26223100	-0.66789300
H	5.38503700	-0.83847000	-0.18730700

H	4.36228600	-0.20914100	-1.46979400
H	4.21044200	0.69669100	1.43999300
H	4.99665800	1.60929300	0.15276000
H	0.83095200	0.87816900	0.03728800
N	1.79069100	0.52539100	0.04139600
O	0.93204900	-1.55286600	0.02372100
H	-2.61078300	-2.33636800	0.62546600
H	-2.93355900	-1.89907300	-1.04894500
H	2.61076300	2.33635100	0.62548200
H	2.93356700	1.89910500	-1.04893700

A6

Electronic energy (E_e)	E_e + ZPV
Hartree	
-439.0350113	-438.900058

XYZ coordinates

H	-1.44858600	2.47543300	-0.00001200
C	-1.40431200	1.39415200	-0.00001300
C	-2.57925800	0.63818600	-0.00004900
C	-0.18830200	0.74231600	0.00002900
H	-3.53825300	1.13698100	-0.00008700
C	-0.15096100	-0.65500600	0.00000600
C	1.22496700	1.25194100	0.00005600
C	-2.52243100	-0.74988900	-0.00007200
C	-1.30088600	-1.42247300	-0.00006200
C	2.05915600	-0.02437900	0.00017800
H	1.47653300	1.84314200	0.88134300

H	-3.43963200	-1.32312200	-0.00007200
H	-1.25500500	-2.50268900	-0.00009700
H	1.48684600	-2.03961100	-0.00011900
N	1.18171200	-1.07796100	0.00001000
O	3.26771000	-0.11707100	-0.00002500
H	1.47659200	1.84307200	-0.88126200

A6 (protonated)

Electronic energy (E_e)	E_e + ZPV
Hartree	
-439.4427198	-439.294569

XYZ coordinates

H	-1.52394500	2.47475600	0.00029100
C	-1.46708200	1.39527300	0.00019900
C	-2.62477400	0.61900800	0.00022200
C	-0.24747600	0.74810400	0.00005900
H	-3.58972300	1.10560900	0.00033700
C	-0.21276800	-0.64133300	-0.00004600
C	1.16509800	1.26041900	-0.00000700
C	-2.56055200	-0.77096200	0.00010700
C	-1.33738100	-1.43655600	-0.00002600
C	1.94231000	-0.00834600	-0.00012400
H	1.43036600	1.84805500	0.88182500
H	-3.47445300	-1.34773700	0.00012800
H	-1.27606100	-2.51518700	-0.00011000
H	1.46753700	-2.01531500	-0.00021200
N	1.15629000	-1.04840100	-0.00014300

O	3.23459000	-0.01777200	-0.00016700
H	1.43024400	1.84809000	-0.88185500
H	3.62104100	-0.91093800	-0.00037400

A6 (deprotonated)

Electronic energy (E_e)	E_e + ZPV
Hartree	
-438.5312682	-438.409747

XYZ coordinates

H	-1.42157200	2.47032000	0.00001900
C	-1.37757600	1.38727200	-0.00000200
C	-2.56203700	0.63687200	-0.00003600
C	-0.16459700	0.73479400	0.00000700
H	-3.52146800	1.13601800	-0.00004300
C	-0.09055000	-0.67855300	-0.00001900
C	1.25249400	1.21505700	0.00004500
C	-2.49524900	-0.75187500	-0.00006200
C	-1.27221600	-1.42197900	-0.00005300
C	2.03464800	-0.11666700	0.00003900
H	1.51978000	1.80079700	0.88106200
H	-3.41292700	-1.32731100	-0.00008900
H	-1.23263400	-2.50372500	-0.00007100
N	1.20115400	-1.17261500	-0.00000200
O	3.27392700	-0.14976800	0.00007000
H	1.51981800	1.80082300	-0.88094300

A6 homodimer

Electronic energy (E_e)	E_e + ZPV
Hartree	
-878.0847035	-877.813883

XYZ coordinates

H	5.83551600	-1.78767800	0.00066400
C	5.26630200	-0.86731700	0.00048400
C	5.91875900	0.36728700	0.00055200
C	3.88660300	-0.89291200	0.00016600
C	3.17104600	0.30761800	-0.00006700
C	2.89809600	-2.02446200	-0.00000500
C	5.19003300	1.55072100	0.00031700
C	3.79586900	1.54004600	0.00000300
C	1.55721800	-1.30525200	-0.00037900
H	2.96248300	-2.66394400	0.88104700
H	5.71051300	2.49872000	0.00037900
H	3.22433900	2.45782500	-0.00017600
H	1.05085900	0.72977000	-0.00059900
N	1.79812300	0.03023500	-0.00036100
O	0.44994800	-1.82694600	-0.00065000
H	2.96291300	-2.66406500	-0.88093800
H	-5.83550000	1.78770400	0.00040400
C	-5.26629600	0.86733700	0.00030200
C	-5.91876600	-0.36726000	0.00035500
C	-3.88659700	0.89291700	0.00010100
C	-3.17105300	-0.30762000	-0.00002800
C	-2.89807900	2.02445800	-0.00005800
C	-5.19005300	-1.55070200	0.00021400

C	-3.79588800	-1.54004100	0.00002000
C	-1.55720900	1.30523400	-0.00022600
H	-2.96256200	2.66405700	0.88089900
H	-5.71054200	-2.49869500	0.00025700
H	-3.22436800	-2.45782700	-0.00008600
H	-1.05086800	-0.72979000	-0.00041000
N	-1.79812600	-0.03025100	-0.00019500
O	-0.44993200	1.82691500	-0.00038400
H	-2.96278900	2.66394400	-0.88108500
H	-6.99916200	-0.40252300	0.00050700
H	6.99915400	0.40256100	0.00079100

A7

Electronic energy (E_e)	E_e + ZPV
Hartree	
-478.3427623	-478.17983

XYZ coordinates

H	1.23416700	2.42761600	-0.46474300
C	1.36721000	1.36979700	-0.27678600
C	2.64635700	0.83966100	-0.09043800
C	0.27753700	0.52540000	-0.21912900
C	0.46744600	-0.83644800	0.02844700
C	-1.19726800	0.78985500	-0.37006300
C	2.81569800	-0.51810300	0.15008600
C	1.72317300	-1.38295400	0.21483600
C	-1.80797500	-0.59662900	-0.15351200
H	-1.43966700	1.08818000	-1.39325100

H	3.81127600	-0.91619600	0.29179600
H	1.85479400	-2.43899200	0.40584000
H	-0.92454600	-2.45531500	0.23939600
N	-0.77744900	-1.47509500	0.05191400
O	-2.98686200	-0.88031300	-0.15057700
H	3.50910000	1.48950400	-0.13288100
C	-1.78077400	1.80777200	0.60766300
H	-1.35120400	2.79261100	0.42912000
H	-2.86070200	1.87043200	0.48253600
H	-1.56459400	1.52023500	1.63678400

A7 (protonated)

Electronic energy (E_e)	E_e + ZPV
Hartree	
-478.7508526	-478.57474

XYZ coordinates

H	-3.23970800	-1.67494200	-0.02540800
H	1.33682400	2.42116700	-0.47415700
C	1.44056800	1.36191400	-0.28217500
C	2.69886600	0.78980000	-0.09891500
C	0.33349300	0.53923100	-0.21580700
C	0.50671000	-0.81586300	0.03451200
C	-1.13836200	0.82819200	-0.36107800
C	2.84214700	-0.57232000	0.14665900
C	1.73354700	-1.41251000	0.22083400
C	-1.71296100	-0.53708900	-0.14999800
H	-1.38788000	1.12336500	-1.38541300

H	3.82949600	-0.98975000	0.28431000
H	1.83217400	-2.47085400	0.41538100
H	-0.94745400	-2.41361500	0.23302900
N	-0.78398900	-1.42532900	0.06421500
O	-2.98819100	-0.74728000	-0.17623100
H	3.57920100	1.41487600	-0.14747900
C	-1.71471900	1.85406900	0.62146700
H	-1.23684700	2.81505800	0.44440600
H	-2.78686200	1.96484000	0.47287900
H	-1.52122400	1.55286000	1.64980300

A7 (deprotonated)

Electronic energy (E_e)	E_e + ZPV
Hartree	
-477.8387278	-477.689254

XYZ coordinates

H	1.21876900	2.41740300	-0.47814300
C	1.35116800	1.35862900	-0.28471500
C	2.63789300	0.83525000	-0.09287000
C	0.26368200	0.51546700	-0.22574800
C	0.41685500	-0.86598700	0.03413300
C	-1.20905700	0.75281000	-0.37143100
C	2.79511600	-0.52420700	0.15420700
C	1.69939400	-1.38480000	0.22101500
C	-1.76362200	-0.67479600	-0.14002000
H	-1.46848800	1.04757000	-1.39223100
H	3.79050300	-0.92534700	0.30125700

H	1.83463600	-2.44015400	0.41981300
N	-0.77722500	-1.56246500	0.08167200
O	-2.98127300	-0.90763000	-0.16145900
H	3.50197700	1.48416800	-0.13602900
C	-1.80838400	1.75513900	0.61006100
H	-1.42747000	2.76165400	0.43080600
H	-2.89360500	1.77143600	0.51027500
H	-1.56382900	1.47654600	1.63642900

A7 homodimer

Electronic energy (E_e)	E_e + ZPV
Hartree	
-956.7002347	-956.372892

XYZ coordinates

H	5.96666700	1.29439400	-0.15584100
C	5.32792600	0.42034200	-0.15139300
C	5.88287800	-0.86161400	-0.14691400
C	3.95478700	0.55298900	-0.15148600
C	3.14627900	-0.58634000	-0.13952500
C	3.06878800	1.77005800	-0.14793900
C	5.06251900	-1.98357100	-0.14077000
C	3.67311100	-1.86399700	-0.13607700
C	1.67324100	1.15294500	-0.11789400
H	3.15313400	2.32265300	-1.08727700
H	5.50605300	-2.96991600	-0.13724200
H	3.03232900	-2.73496500	-0.12764100
H	0.99597300	-0.82950400	-0.10082000

N	1.80004900	-0.19782500	-0.12681400
O	0.61610500	1.76883900	-0.08203600
H	-5.96666400	-1.29441100	-0.15564400
C	-5.32792800	-0.42035600	-0.15122800
C	-5.88288600	0.86159800	-0.14678000
C	-3.95478800	-0.55299600	-0.15133600
C	-3.14628500	0.58633800	-0.13942300
C	-3.06878400	-1.77006200	-0.14776900
C	-5.06253300	1.98355900	-0.14068200
C	-3.67312400	1.86399200	-0.13600700
C	-1.67324000	-1.15294200	-0.11776300
H	-3.15314600	-2.32268700	-1.08708700
H	-5.50607200	2.96990100	-0.13717700
H	-3.03234600	2.73496300	-0.12760900
H	-0.99597600	0.82950700	-0.10072500
N	-1.80005300	0.19782800	-0.12673600
O	-0.61609800	-1.76882700	-0.08188900
H	-6.95695300	0.98292000	-0.14806100
H	6.95694400	-0.98294200	-0.14820700
C	-3.27848100	-2.72662900	1.02474100
H	-4.27087200	-3.17208400	0.97346200
H	-2.53689700	-3.52358800	0.99426000
H	-3.18546300	-2.19791400	1.97351300
C	3.27850800	2.72666100	1.02453600
H	4.27089900	3.17211400	0.97322600
H	2.53692400	3.52362100	0.99404100
H	3.18550500	2.19797700	1.97332700

I1

Electronic energy (E_e)	E_e + ZPV
Hartree	
-478.5844151	-478.406679

XYZ coordinates

C	0.99896900	1.23963300	-0.12011000
C	0.99803900	-1.24009500	-0.12045900
C	-0.46359800	-1.23662900	-0.48304300
C	-1.21852500	0.00034200	0.01359400
C	-0.46304400	1.23758300	-0.48151300
H	-0.51027700	-1.28433200	-1.57583400
H	-0.89625100	-2.15777800	-0.09494200
H	2.58986100	-0.00081900	0.23392200
N	1.60559300	-0.00049500	-0.00892500
O	1.64615800	-2.24805300	0.05034800
O	1.64807300	2.24702000	0.05017500
H	-0.89470600	2.15834600	-0.09135900
H	-0.51049700	1.28747800	-1.57416100
C	-2.62945900	0.00092400	-0.56775900
H	-3.18009800	-0.88244100	-0.24016700
H	-3.17993400	0.88392600	-0.23890600
H	-2.60418800	0.00169800	-1.65873300
C	-1.29552700	-0.00049100	1.54281200
H	-1.83080000	0.88236300	1.89522300
H	-1.83003700	-0.88410400	1.89439600
H	-0.30720600	-0.00020700	2.00772800

I1 (protonated)

Electronic energy (E_e)	E_e + ZPV
Hartree	
-478.9830873	-478.792182

XYZ coordinates

C	-1.11930000	-1.00201800	-0.09826000
C	-0.74584600	1.41077000	-0.14980000
C	0.66404500	1.15512500	-0.57444300
C	1.24112700	-0.13773900	0.01720900
C	0.30712900	-1.29375600	-0.36459900
H	0.64880600	1.09298500	-1.66807900
H	1.25290000	2.02704600	-0.29630200
H	-2.54643600	0.40599300	0.20834600
N	-1.56600400	0.22489600	-0.00548500
O	-1.27472200	2.45834400	0.05364200
O	-1.90623300	-2.01664300	0.02531000
H	0.55822900	-2.21862000	0.15607300
H	0.36481200	-1.50548900	-1.43935200
C	2.61790000	-0.39847800	-0.58486800
H	3.30038100	0.41035100	-0.32385100
H	3.03125200	-1.33039300	-0.19732200
H	2.56623500	-0.46894500	-1.67203800
C	1.35280000	-0.02490400	1.53934200
H	1.79075600	-0.93318000	1.95334200
H	1.99554300	0.81462900	1.80419200
H	0.38505200	0.13067600	2.02307600
H	-2.84499200	-1.78692900	0.15120700

I1 (deprotonated)

Electronic energy (E_e)	E_e + ZPV
Hartree	
-478.079397972	-477.915378

XYZ coordinates

C	-1.06669600	-1.17470000	-0.09559200
C	-1.06627500	1.17495200	-0.09576600
C	0.41566400	1.21932800	-0.46574900
C	1.20173300	-0.00017200	0.00490300
C	0.41535900	-1.21972600	-0.46508200
H	0.46679500	1.28630400	-1.55784000
H	0.83012800	2.14517300	-0.06501800
N	-1.71509500	0.00025000	0.06168200
O	-1.64976800	2.25774600	0.02888300
O	-1.65066000	-2.25727200	0.02885800
H	0.82941600	-2.14543500	-0.06361600
H	0.46674100	-1.28753100	-1.55711300
C	2.60159000	-0.00048000	-0.60369800
H	3.16282600	0.88314400	-0.29044200
H	3.16260100	-0.88413300	-0.29012200
H	2.55351800	-0.00066500	-1.69454500
C	1.31444200	0.00023000	1.53192600
H	1.85536700	-0.88347500	1.87708500
H	1.85547000	0.88405400	1.87662000
H	0.33131600	0.00041800	2.00564500

I1 homodimer

Electronic energy (E_e)	E_e + ZPV
Hartree	
-957.1793819	-956.822554

XYZ coordinates

C	2.85405400	-1.56820100	-0.27129100
C	1.97300600	0.72829200	-0.41707600
C	3.37473600	1.27412500	-0.43281100
C	4.38691300	0.38216800	0.29181600
C	4.26031300	-1.02858000	-0.29038400
H	3.65423900	1.37186600	-1.48709100
H	3.33941800	2.27631500	-0.00799100
H	4.57427400	-1.02498600	-1.33939700
H	4.89054400	-1.74244700	0.23832200
H	0.87227600	-0.99690300	-0.38592000
N	1.83075100	-0.63766400	-0.37763900
O	0.98821800	1.44513400	-0.45922200
O	2.59005000	-2.74634300	-0.19449800
C	-2.85404800	1.56818700	-0.27141200
C	-1.97299700	-0.72831300	-0.41706600
C	-3.37472700	-1.27414700	-0.43279700
C	-4.38691900	-0.38215300	0.29176300
C	-4.26030600	1.02856600	-0.29050500
H	-3.65421000	-1.37194500	-1.48707700
H	-3.33941600	-2.27631500	-0.00792300
H	-4.57424500	1.02491600	-1.33952500
H	-4.89054900	1.74246000	0.23815000
H	-0.87226800	0.99688300	-0.38598000

N	-1.83074300	0.63764400	-0.37769700
O	-0.98820900	-1.44515700	-0.45916000
O	-2.59004500	2.74633300	-0.19467300
C	-4.09790200	-0.36481300	1.79542800
H	-4.81377200	0.27635000	2.31154800
H	-4.18108600	-1.37016900	2.21064100
H	-3.09545000	0.00724000	2.01775700
C	-5.79768800	-0.91000400	0.04857000
H	-5.90309400	-1.92147100	0.44447300
H	-6.53464000	-0.27552800	0.54382000
H	-6.02978500	-0.93484300	-1.01729100
C	4.09786400	0.36490400	1.79547500
H	4.81372400	-0.27622900	2.31164500
H	4.18103400	1.37028100	2.21063900
H	3.09540800	-0.00714200	2.01780000
C	5.79768700	0.91000800	0.04862500
H	5.90308400	1.92149400	0.44448200
H	6.53463000	0.27555700	0.54392000
H	6.02980500	0.93479800	-1.01723300

I2

Electronic energy (E_e)	E_e + ZPV
Hartree	
-517.8863724	-517.680422

XYZ coordinates

O	0.81405700	2.27557000	-0.65797000
N	2.07512500	0.36606400	-0.56758300

C	0.94202200	1.12035000	-0.33724000
C	1.98533000	-0.93515100	-0.11529200
C	0.60659400	-1.12050900	0.47756900
C	-0.08142300	0.25474100	0.39406500
H	2.90166400	0.73637400	-1.01898400
H	0.70869500	-1.49438200	1.49547200
H	0.09245500	-1.88628600	-0.10424500
O	2.86829300	-1.75159500	-0.20323300
C	-1.37301500	0.23161200	-0.43762500
H	-1.68001200	1.26321200	-0.62494400
H	-1.14914100	-0.21425200	-1.41213300
C	-2.52286600	-0.53220100	0.21268200
H	-2.18429300	-1.52650700	0.51656300
H	-2.83700900	-0.01655800	1.12164300
C	-3.71338900	-0.66366000	-0.73089600
H	-4.54528300	-1.17928500	-0.25115800
H	-4.06705500	0.31826800	-1.04984800
H	-3.44239200	-1.22521400	-1.62643100
C	-0.29833900	0.87546900	1.77770200
H	-0.75766600	1.85963800	1.68533500
H	-0.94519400	0.24038600	2.38113300
H	0.65107700	0.98644600	2.30450100

I2 (protonated)

Electronic energy (E_e)	E_e + ZPV
Hartree	
-518.2826477	-518.064321

XYZ coordinates

O	0.76070200	2.25757600	-0.55139800
N	2.04518700	0.34571000	-0.55434900
C	0.95972800	1.01907900	-0.28163300
C	1.94270500	-1.02680600	-0.11332000
C	0.57243100	-1.17962700	0.48815000
C	-0.08843100	0.21273600	0.41925600
H	2.87705600	0.71321600	-1.01219500
H	0.67622400	-1.55490100	1.50510300
H	0.03238600	-1.92909900	-0.09084200
O	2.83115700	-1.79863500	-0.25580500
C	-1.37279000	0.23436200	-0.43655500
H	-1.66781800	1.27287400	-0.60062100
H	-1.14292900	-0.19867700	-1.41456700
C	-2.53027500	-0.53151000	0.19747400
H	-2.19970500	-1.53223000	0.48639800
H	-2.84830300	-0.02606000	1.11014900
C	-3.70925500	-0.63763300	-0.76306400
H	-4.54753800	-1.15374200	-0.29677600
H	-4.05305200	0.35127600	-1.06971800
H	-3.43328600	-1.18962400	-1.66259700
C	-0.30304400	0.83171400	1.80954900
H	-0.76457100	1.81471400	1.72703100
H	-0.95692900	0.18256400	2.38718800

H	0.64246100	0.92586000	2.34487100
H	1.50841700	2.69844300	-0.99449700

I2 (deprotonated)

Electronic energy (E_e)	E_e + ZPV
Hartree	
-517.390075609	-517.196955

XYZ coordinates

O	0.78907700	2.26127800	-0.68977100
N	2.14809400	0.40661100	-0.64047200
C	1.00678000	1.09094700	-0.38134200
C	2.02815100	-0.85162600	-0.15376400
C	0.65994200	-1.09839900	0.48419500
C	-0.04296200	0.25355000	0.38495200
H	0.79453000	-1.45424400	1.50622700
H	0.15390300	-1.88888300	-0.07306000
O	2.89281700	-1.72579700	-0.20396000
C	-1.33808200	0.20919500	-0.43529600
H	-1.64378100	1.23597800	-0.65327500
H	-1.11868400	-0.26265300	-1.39920600
C	-2.49291700	-0.53415900	0.23144100
H	-2.15659500	-1.52273100	0.55700700
H	-2.80242500	0.00128600	1.13119300
C	-3.69044900	-0.68373700	-0.70121400
H	-4.52190800	-1.19079800	-0.21003000
H	-4.04526000	0.29239200	-1.03749400
H	-3.42390800	-1.26075200	-1.58858400

C	-0.26236500	0.90118100	1.75411600
H	-0.71268700	1.88876400	1.64385600
H	-0.91255300	0.28968000	2.38122100
H	0.68897500	1.02013900	2.27677800

I2 homodimer

Electronic energy (E_e)	E_e + ZPV
Hartree	
-1035.784487	-1035.371707

XYZ coordinates

O	1.44298600	1.10682400	-0.27626700
N	1.45858200	-1.18118500	-0.29209900
C	2.04839800	0.05390100	-0.30483900
C	2.36690400	-2.22515000	-0.31981200
C	3.75730200	-1.62946500	-0.30839600
C	3.56230900	-0.10472500	-0.38479100
H	0.44287700	-1.30375700	-0.27445500
H	4.32571700	-2.03865800	-1.14234200
H	4.24662400	-1.94525100	0.61382200
O	-1.44334200	-1.10744300	-0.27585600
N	-1.45843400	1.18057400	-0.29216700
C	-2.04850500	-0.05439400	-0.30484700
C	-2.36652500	2.22471300	-0.32059800
C	-3.75706500	1.62934200	-0.30971200
C	-3.56237500	0.10451700	-0.38513200
H	-0.44267700	1.30293700	-0.27431900
H	-4.24698700	1.94580300	0.61195300

H	-4.32479500	2.03816000	-1.14431500
O	-2.06407700	3.39088200	-0.33919500
O	2.06470200	-3.39137000	-0.33824000
C	-4.19536400	-0.64773300	0.79452400
H	-3.84218700	-1.68119500	0.77401500
H	-3.82701500	-0.20528900	1.72565000
C	-5.72108700	-0.63156900	0.79951000
H	-6.08305800	0.39555800	0.70248500
H	-6.09640400	-1.17933400	-0.06637600
C	-6.28323300	-1.25439700	2.07237000
H	-7.37297600	-1.27170500	2.05822000
H	-5.93254800	-2.28117800	2.18931900
H	-5.96812400	-0.69383600	2.95398500
C	-4.00701800	-0.46380700	-1.73711100
H	-3.81790800	-1.53614600	-1.78108900
H	-5.07072800	-0.28692300	-1.88884500
H	-3.46687500	0.01714600	-2.55448300
C	4.19516400	0.64847700	0.79430400
H	3.84173200	1.68183700	0.77312200
H	3.82700000	0.20656800	1.72575500
C	5.72089600	0.63269400	0.79914600
H	6.08311100	-0.39438000	0.70247800
H	6.09597200	1.18019600	-0.06701500
C	6.28305100	1.25619200	2.07167100
H	7.37278400	1.27390500	2.05730900
H	5.93199500	2.28288300	2.18831900
H	5.96833200	0.69584000	2.95355600
C	4.00683100	0.46272800	-1.73719600
H	3.81751100	1.53499600	-1.78193500

H	5.07057500	0.28594100	-1.88881800
H	3.46677100	-0.01892800	-2.55421300

I3

Electronic energy (E_e)	E_e + ZPV
Hartree	
-670.2978915	-670.067806

XYZ coordinates

H	-0.68300200	1.83910200	-0.00003400
C	-0.35898100	0.80683100	-0.00004400
C	-1.28745100	-0.24738300	-0.00005700
C	0.98174500	0.50252900	-0.00001600
C	1.43877600	-0.80832300	0.00005200
C	2.16916200	1.40790900	-0.00000600
C	-0.80950400	-1.56118800	0.00002200
C	0.55431300	-1.86260200	0.00005900
H	-1.51013200	-2.38242900	0.00007300
H	0.89932800	-2.88748200	0.00011700
O	3.70405300	-1.70452700	-0.00001900
O	2.20464200	2.61116000	0.00015500
C	2.92860200	-0.78373500	0.00009700
N	3.27458900	0.56483900	-0.00019300
H	4.22874100	0.89616500	-0.00043800
C	-2.78039000	0.08230400	-0.00001800
C	-3.11281700	0.90861400	-1.25226800
H	-4.17659600	1.15202100	-1.26099700

H	-2.55443200	1.84448100	-1.27558900
H	-2.88209500	0.35075800	-2.16099300
C	-3.11252700	0.90936300	1.25181800
H	-4.17630300	1.15277000	1.26067400
H	-2.88156400	0.35207400	2.16082900
H	-2.55412900	1.84524200	1.27441100
C	-3.65207800	-1.17380100	0.00044500
H	-3.47646500	-1.78569800	-0.88544700
H	-3.47564800	-1.78560500	0.88624300
H	-4.70248200	-0.88144000	0.00091500

I3 (protonated)

Electronic energy (E_e)	E_e + ZPV
Hartree	
-670.6910814	-670.448315

XYZ coordinates

H	0.75902400	1.85580300	0.00027100
C	0.41515700	0.83085700	0.00011700
C	1.32512000	-0.24750400	-0.00008400
C	-0.92409800	0.55042900	0.00009300
C	-1.40329900	-0.76058500	-0.00010500
C	-2.09066400	1.46654100	0.00022700
C	0.82571500	-1.55251100	-0.00029200
C	-0.54179000	-1.83587800	-0.00030300
H	1.51304000	-2.38450200	-0.00044500
H	-0.90061800	-2.85523400	-0.00045500
O	-3.62183100	-1.70714600	-0.00014600

O	-2.18322800	2.64859000	0.00052300
C	-2.85290800	-0.67923300	-0.00002400
N	-3.24076200	0.58245600	0.00024600
H	-4.19829700	0.91956500	0.00040500
C	2.81854200	0.06012800	-0.00007300
C	3.15532700	0.88446200	1.25349300
H	4.22253600	1.10895100	1.26147700
H	2.61445700	1.83061600	1.27216900
H	2.91518900	0.33184800	2.16269200
C	3.15526400	0.88484600	-1.25340500
H	4.22247100	1.10934400	-1.26136900
H	2.91508900	0.33250600	-2.16276100
H	2.61438700	1.83100200	-1.27176800
C	3.67112500	-1.20795500	-0.00029000
H	3.48756900	-1.81637600	0.88622500
H	3.48753100	-1.81609800	-0.88698700
H	4.72438700	-0.92839200	-0.00026900
H	-4.57190100	-1.48935000	-0.00004200

I3 (deprotonated)

Electronic energy (E_e)	E_e + ZPV
Hartree	
-669.8039591	-669.586987

XYZ coordinates

H	0.65337800	1.83759800	0.00009100
C	0.32433300	0.80602000	0.00007200
C	1.25263800	-0.25094200	-0.00007000

C	-1.01814800	0.50924700	0.00000000
C	-1.48057600	-0.79341300	-0.00006800
C	-2.25193100	1.38822600	-0.00032300
C	0.76994100	-1.56120300	-0.00027300
C	-0.60057600	-1.85119000	-0.00028000
H	1.46634100	-2.38682700	-0.00037800
H	-0.94872300	-2.87618900	-0.00030800
O	-3.73130300	-1.67597000	-0.00033000
O	-2.20637500	2.61363700	0.00084200
C	-2.99144100	-0.69847900	0.00039800
N	-3.36989700	0.61119600	0.00025400
C	2.74822900	0.07576500	-0.00006600
C	3.08767700	0.90098200	1.25103300
H	4.15163000	1.14682100	1.25898500
H	2.52663200	1.83510200	1.27850100
H	2.85847500	0.34198200	2.15964100
C	3.08759000	0.90142000	-1.25089900
H	4.15154100	1.14726500	-1.25883900
H	2.85832800	0.34273900	-2.15968800
H	2.52654100	1.83554900	-1.27800200
C	3.62032400	-1.18081400	-0.00031500
H	3.44307000	-1.79347800	0.88484700
H	3.44301000	-1.79316600	-0.88568000
H	4.67211200	-0.89081600	-0.00030000

I3 homodimer

Electronic energy (E_e)	E_e + ZPV
Hartree	
-1340.606438	-1340.145607

XYZ coordinates

C	3.64612700	-1.73908200	0.05810200
C	2.28277900	-2.34167300	0.07385900
C	3.50780200	-0.35884100	0.01063900
C	2.05035700	-0.04784300	-0.00069700
H	0.38207500	-1.34750100	0.02450600
N	1.39833300	-1.26279100	0.03769400
O	1.51740700	1.04010800	-0.03569700
C	-3.64648800	1.73934400	0.05816800
C	-2.28330600	2.34229900	0.07397400
C	-3.50779400	0.35913300	0.01082600
C	-2.05027400	0.04850800	-0.00019300
H	-0.38237700	1.34867600	0.02480600
N	-1.39857400	1.26362700	0.03815400
O	-1.51703000	-1.03931100	-0.03492900
O	-1.96768500	3.50253500	0.10851600
C	-4.89317100	2.32087000	0.07800400
C	-6.00192100	1.47172300	0.04576200
H	-6.98389600	1.91987000	0.06142600
C	-5.88084600	0.07995400	-0.00697900
C	-4.59375100	-0.48338500	-0.02157000
H	-5.01491100	3.39466800	0.11613000
H	-4.44889500	-1.55483700	-0.05865400
O	1.96687400	-3.50184600	0.10858300

C	4.89265200	-2.32092900	0.07811300
C	6.00162200	-1.47205900	0.04591200
C	5.88090600	-0.08025600	-0.00683900
C	4.59396100	0.48341700	-0.02163400
H	4.44935900	1.55490000	-0.05876900
H	5.01410500	-3.39475600	0.11632800
H	6.98348400	-1.92044900	0.06183700
C	-7.09381300	-0.84859600	-0.05609100
C	-7.04216200	-1.66675600	-1.35553800
H	-6.13729800	-2.27179400	-1.41445900
H	-7.90001900	-2.33969200	-1.40079100
H	-7.07288700	-1.01382400	-2.22906500
C	-7.04722400	-1.80354900	1.14640600
H	-7.07002800	-1.24833000	2.08540900
H	-7.91155500	-2.46917900	1.12184700
H	-6.14932100	-2.42141000	1.13793400
C	-8.41679300	-0.08287100	-0.01761900
H	-8.52292400	0.49597300	0.90107800
H	-8.51855000	0.59317900	-0.86789300
H	-9.24165500	-0.79470600	-0.05927100
C	7.09413800	0.84795400	-0.05601800
C	8.41688800	0.08184200	-0.01722800
H	9.24196800	0.79342200	-0.05895000
H	8.52275600	-0.49683800	0.90160300
H	8.51854800	-0.59442000	-0.86734300
C	7.04287000	1.66580900	-1.35567100
H	7.07358300	1.01264800	-2.22902700
H	6.13812700	2.27100300	-1.41488200
H	7.90086900	2.33856200	-1.40096200

C	7.04775600	1.80317700	1.14627500
H	7.91238000	2.46842600	1.12171500
H	6.15012100	2.42142300	1.13757700
H	7.07020200	1.24811300	2.08538100

I4

Electronic energy (E_e)	E_e + ZPV
Hartree	
-359.4207477	-359.352346

XYZ coordinates

C	1.14192100	-0.15770600	0.00007500
C	-1.14191300	-0.15809800	-0.00006000
C	0.66330800	1.26467300	0.00013900
H	0.00014800	-1.95119500	-0.00011400
N	0.00002400	-0.94218600	-0.00003400
O	-2.27636900	-0.55777800	-0.00025500
H	1.34901900	2.09661000	0.00027100
C	-0.66315500	1.26450700	-0.00005700
H	-1.34918700	2.09621700	-0.00013700
O	2.27622900	-0.55804500	0.00021000

I4 (protonated)

Electronic energy (E_e)	E_e + ZPV
Hartree	
-359.8087917	-359.727835

XYZ coordinates

C	1.02385300	-0.13163400	0.00011200
C	-1.21182300	-0.14869800	-0.00028200
C	0.58762400	1.27836000	0.00007300
H	0.02517100	-1.95383500	0.00009800
N	-0.00746900	-0.93925500	0.00006200
O	-2.30386100	-0.60287700	-0.00024500
H	1.27911000	2.10635100	0.00010700
C	-0.74056700	1.27555800	0.00010800
H	-1.42653500	2.10730100	0.00021200
O	2.22033600	-0.57938800	0.00011700
H	2.88821400	0.13156900	0.00010000

I4 (deprotonated)

Electronic energy (E_e)	E_e + ZPV
Hartree	
-358.92754	-358.871828

XYZ coordinates

C	1.09033500	-0.22361300	0.00063800
C	-1.09037500	-0.22393400	-0.00012300
C	0.66132300	1.23829100	-0.00015000
N	-0.00006000	-1.03406900	-0.00011800

O	-2.26803100	-0.56633500	-0.00001600
H	1.35851600	2.06295900	-0.00049300
C	-0.66111500	1.23849100	0.00010300
H	-1.35826800	2.06322500	0.00013500
O	2.26792600	-0.56655400	-0.00018800

I4 homodimer

Electronic energy (E_e)	E_e + ZPV
Hartree	
-718.8519154	-718.71311

XYZ coordinates

C	3.17895700	-0.75466000	-0.00014200
C	1.77223400	1.02865000	-0.00008900
C	4.00219700	0.50154300	0.00044200
H	1.04064800	-0.95916200	-0.00051800
N	1.85239200	-0.34384500	-0.00064100
O	0.75399800	1.68369400	-0.00006600
C	-3.17898600	0.75464300	-0.00006200
C	-1.77221100	-1.02862600	-0.00006400
C	-4.00219000	-0.50158300	0.00052200
H	-1.04068700	0.95921000	-0.00044200
N	-1.85240800	0.34386700	-0.00061000
O	-0.75395700	-1.68364200	-0.00004300
H	5.07992200	0.47833500	0.00074700
H	-5.07991500	-0.47840600	0.00084600
C	-3.18026600	-1.54229100	0.00050600
C	3.18030300	1.54227500	0.00045900

H	-3.40432200	-2.59681200	0.00081200
H	3.40438900	2.59679000	0.00077900
O	3.56708900	-1.89182400	-0.00019100
O	-3.56714800	1.89179400	-0.00006300

I5

Electronic energy (E_e)	E_e + ZPV
Hartree	
-650.6657051	-650.402994

XYZ coordinates

O	4.40654100	-0.48560700	0.05197100
N	0.40004100	0.09087900	-0.02967900
N	2.59882400	0.86456300	0.04529500
C	1.23531700	1.14834100	-0.13963100
C	3.20095500	-0.37055400	0.01197000
C	0.90122700	-1.16669900	0.51970600
C	2.23946600	-1.52057800	-0.10238400
H	0.17658800	-1.94737300	0.30533900
H	2.11728900	-1.73219700	-1.16779700
H	3.20376500	1.67422800	0.00156900
O	0.90063700	2.29646100	-0.38126200
H	2.68363400	-2.39241000	0.37129300
H	0.99942700	-1.09012500	1.60641500
C	-1.05005800	0.31490100	-0.09715600
C	-1.69704500	-0.53188000	-1.19263500
C	-1.71783400	0.08005500	1.25854900
H	-1.17098600	1.36430500	-0.36138800

C	-3.19950700	-0.25485000	-1.26804000
H	-1.53979400	-1.59383200	-0.98140500
H	-1.21541500	-0.31419500	-2.14715300
C	-3.21888800	0.35809900	1.17294800
H	-1.56469100	-0.95817000	1.56832000
H	-1.24911200	0.71874400	2.00971900
C	-3.87477600	-0.48757100	0.08256500
H	-3.65298300	-0.88333100	-2.03553500
H	-3.35664400	0.78398900	-1.57305300
H	-3.68668800	0.16606100	2.13943000
H	-3.37498100	1.41743300	0.94848700
H	-4.93916200	-0.25740600	0.01608100
H	-3.79287200	-1.54622700	0.34934300

I5 (protonated in the oxygen of the carbonyl in position 2)

Electronic energy (E_e)	E_e + ZPV
Hartree	
-651.0749587	-650.799463

XYZ coordinates

O	-4.37356200	-0.56787000	0.05080600
N	-0.39204800	0.12237600	-0.14645600
N	-2.60347900	0.82127500	-0.11018500
C	-1.27766100	1.07275700	-0.04358500
C	-3.18234800	-0.44793200	0.06165000
C	-0.88361100	-1.23986000	-0.45116300
C	-2.17312100	-1.53086600	0.29194900
H	-0.11612900	-1.94261800	-0.14917700

H	-1.99265000	-1.57725300	1.36917800
H	-3.22288600	1.62488200	-0.12230300
O	-1.01428800	2.34235100	0.12765500
H	-2.59682400	-2.47929200	-0.02655100
H	-1.02173800	-1.31555900	-1.53002200
C	1.07527100	0.33778300	-0.04741500
C	1.62481600	-0.26055100	1.24604900
C	1.78803300	-0.20272900	-1.28603900
H	1.26090400	1.41260800	-0.01937800
C	3.12930500	-0.00018600	1.33828500
H	1.44302000	-1.33810300	1.25603900
H	1.10445900	0.17274800	2.10119700
C	3.28912900	0.06958700	-1.17339900
H	1.62878000	-1.28027200	-1.36371400
H	1.37194400	0.26120400	-2.18141000
C	3.86261100	-0.53125800	0.10832800
H	3.51874200	-0.45926500	2.24648600
H	3.30327100	1.07619300	1.42633600
H	3.79357800	-0.33853500	-2.04868100
H	3.46365200	1.14934000	-1.17845900
H	4.92700100	-0.30855400	0.18270600
H	3.76505000	-1.62020800	0.07091300
H	-0.07323100	2.56081000	0.06767800

I5 (protonated in the oxygen of the carbonyl in position 4)

Electronic energy (E_e)	E_e + ZPV
Hartree	
-651.0675076	-650.791884

XYZ coordinates

O	-4.35400300	-0.59627300	0.05127000
N	-0.34960900	0.13036200	-0.07286900
N	-2.59087000	0.84352800	-0.06554900
C	-1.17108200	1.17038800	0.06478200
C	-3.08577900	-0.35685200	0.02553100
C	-0.84875100	-1.15938600	-0.55207700
C	-2.16444400	-1.50810800	0.12091700
H	-0.11392600	-1.92133900	-0.31500500
H	-2.02688100	-1.70946500	1.18838600
H	-3.19017900	1.66682300	-0.07754400
O	-0.91420200	2.32558700	0.29762400
H	-2.63627900	-2.37734800	-0.33144800
H	-0.97312200	-1.13146200	-1.63593300
C	1.10900800	0.34547500	0.03444000
C	1.68879900	-0.43755500	1.21050500
C	1.81365000	0.00516900	-1.27740800
H	1.23352100	1.40924700	0.23082600
C	3.19214900	-0.18158400	1.32495200
H	1.51908100	-1.50794400	1.06116700
H	1.17589100	-0.14591100	2.12820700
C	3.31500200	0.26561600	-1.14913600
H	1.65449300	-1.05018100	-1.51711700
H	1.38470700	0.59777400	-2.08747400

C	3.91194400	-0.51615500	0.01959000
H	3.60127800	-0.76619400	2.14920600
H	3.35784000	0.87199700	1.56707700
H	3.81357000	0.00105400	-2.08180800
H	3.48133400	1.33482100	-0.98999700
H	4.97653300	-0.29904600	0.11199700
H	3.82060100	-1.58847100	-0.17955400
H	-4.91244500	0.20185800	0.02420800

I5 (deprotonated)

Electronic energy (E_e)	E_e + ZPV
Hartree	
-650.1572283	-649.908293

XYZ coordinates

O	4.40116800	-0.43579900	0.28254800
N	0.43895300	0.06008300	-0.15753800
N	2.64309700	0.97551100	0.04013500
C	1.30565600	1.14975100	-0.19550400
C	3.19557100	-0.24578500	0.06359900
C	0.93541500	-1.20429000	0.35464200
C	2.31279600	-1.45245600	-0.21842700
H	0.24669400	-1.99837200	0.06896300
H	2.24514100	-1.58522700	-1.30174000
O	0.84640400	2.27425200	-0.43775900
H	2.77370400	-2.33955000	0.21034100
H	0.98995600	-1.19171700	1.45092000
C	-1.00339900	0.28909300	-0.13583500

C	-1.73379100	-0.54344000	-1.19220400
C	-1.60964000	0.05457800	1.25188000
H	-1.13371000	1.33974000	-0.38818600
C	-3.23230300	-0.23674700	-1.18815700
H	-1.59019200	-1.60973000	-0.99117200
H	-1.29963400	-0.33667900	-2.17177400
C	-3.10739300	0.36180500	1.25408000
H	-1.46008800	-0.98973300	1.54446900
H	-1.08746100	0.67445900	1.98361300
C	-3.83989900	-0.46242700	0.19604200
H	-3.74289800	-0.85023200	-1.93257000
H	-3.38422200	0.80741200	-1.47823700
H	-3.52845600	0.17263700	2.24316200
H	-3.25458000	1.42553200	1.04374000
H	-4.90240300	-0.21134600	0.18880000
H	-3.76484200	-1.52448500	0.45249600

I5 homodimer

Electronic energy (E_e)	E_e + ZPV
Hartree	
-1301.344274	-1300.816632

XYZ coordinates

O	0.55485500	3.68775100	-0.84010800
N	3.32999700	0.84535900	-0.09385000
N	1.12171100	1.56253300	-0.33497200
C	2.02806900	0.51118300	-0.20199400
C	1.42469200	2.86408800	-0.65484500

C	3.69288500	2.24115800	0.13997100
C	2.89654600	3.14924700	-0.77733300
H	4.75608400	2.35452900	-0.05216800
H	3.17512400	2.97124900	-1.81918900
H	0.13672500	1.28202800	-0.33343300
O	-0.55458700	-3.68747400	-0.84005000
N	-3.33002500	-0.84533200	-0.09400600
N	-1.12162200	-1.56236200	-0.33463100
C	-2.02806400	-0.51108700	-0.20170300
C	-1.42449600	-2.86391400	-0.65465700
C	-3.69296600	-2.24116400	0.13950000
C	-2.89629000	-3.14909600	-0.77765500
H	-3.51045300	-2.50562000	1.18501600
H	-3.06733400	-4.19799900	-0.55007600
H	-0.13663200	-1.28179900	-0.33311700
O	-1.60415400	0.64289000	-0.18603600
O	1.60412900	-0.64279400	-0.18695900
H	-3.17453600	-2.97098600	-1.81958500
H	-4.75609200	-2.35453400	-0.05305600
H	3.06755900	4.19811500	-0.54956000
H	3.50998700	2.50545300	1.18546100
C	-4.32872600	0.20476700	0.14414200
C	-5.44190900	0.17049600	-0.90312600
C	-4.89435300	0.12564000	1.56276200
H	-3.79390400	1.14706900	0.04011400
C	-6.44455600	1.29789900	-0.65299900
H	-5.96687700	-0.78832400	-0.85848400
H	-5.00288500	0.25856500	-1.89810000
C	-5.89921300	1.25213600	1.80370800

H	-5.39560200	-0.83706000	1.70259700
H	-4.07607000	0.17731400	2.28368200
C	-7.01660700	1.22508200	0.76195800
H	-7.24519000	1.25015400	-1.39237000
H	-5.94190600	2.26010500	-0.78769700
H	-6.31248300	1.16943700	2.80968700
H	-5.37995900	2.21344700	1.74889100
H	-7.71172200	2.04848500	0.93123600
H	-7.58719400	0.29724700	0.87127600
C	4.32863200	-0.20481900	0.14416600
C	5.44186300	-0.17046400	-0.90305300
C	4.89422000	-0.12594600	1.56282100
H	3.79375600	-1.14707500	0.03997900
C	6.44447200	-1.29792500	-0.65302700
H	5.96682600	0.78835100	-0.85825500
H	5.00288200	-0.25840400	-1.89805600
C	5.89905300	-1.25248100	1.80365200
H	5.39549000	0.83671900	1.70281900
H	4.07591600	-0.17770600	2.28370900
C	7.01649300	-1.22529900	0.76195200
H	7.24511000	-1.25012700	-1.39238900
H	5.94178000	-2.26009400	-0.78783500
H	6.31227300	-1.16991900	2.80966300
H	5.37981100	-2.21378900	1.74867600
H	7.71160000	-2.04872500	0.93114400
H	7.58708600	-0.29748100	0.87140600

I6

Electronic energy (E_e)	E_e + ZPV
Hartree	
-3223.020819	-3222.791119

XYZ coordinates

O	-0.95237900	2.97875500	0.00018600
N	1.18846600	2.23784000	-0.00122800
N	-0.57028900	0.71930900	-0.00014300
C	-0.17643900	2.04584200	-0.00005100
C	0.35375300	-0.28700100	0.00037800
C	1.68037200	-0.07354900	0.00025600
C	2.19933400	1.28431900	0.00013200
H	1.48633400	3.20602600	-0.00089300
O	3.36599800	1.61089900	0.00052200
H	-0.05159400	-1.28848200	0.00049000
C	-2.01527900	0.39523300	-0.00010200
C	-2.41145100	-0.36336700	-1.26421100
C	-2.41154400	-0.36280200	1.26427200
H	-2.51488200	1.36149100	-0.00024500
C	-3.91495600	-0.64158300	-1.25911900
H	-1.87053400	-1.31376600	-1.30572200
H	-2.12634600	0.21701300	-2.14287500
C	-3.91517500	-0.64085400	1.25907100
H	-1.87083500	-1.31331900	1.30597300
H	-2.12649200	0.21772800	2.14285100
C	-4.33187100	-1.39967200	0.00017100
H	-4.18587400	-1.20459500	-2.15265900
H	-4.45431000	0.30907500	-1.30358200
H	-4.18657300	-1.20305200	2.15297000

H	-4.45422300	0.31002300	1.30257400
H	-5.40985700	-1.56480300	0.00007500
H	-3.85750900	-2.38602000	0.00044500
Br	2.90417200	-1.50011400	-0.00000700

I6 (protonated)

Electronic energy (E_e)	E_e + ZPV
Hartree	
-3223.421957	-3223.179139

XYZ coordinates

O	0.92166400	2.98019800	-0.00018800
N	-1.20895700	2.20349400	-0.00010400
N	0.58564200	0.70591900	-0.00030800
C	0.18208900	2.03695300	-0.00053900
C	-0.30295900	-0.29680000	-0.00022200
C	-1.65601000	-0.10368400	-0.00010800
C	-2.11253500	1.21776600	0.00004100
H	-1.51352300	3.17551400	0.00010000
O	-3.38726400	1.46575000	0.00041400
H	0.10967300	-1.29702400	-0.00024900
C	2.05174100	0.40764400	-0.00019000
C	2.44328200	-0.34575100	1.26655100
C	2.44330200	-0.34646700	-1.26648200
H	2.52849400	1.38474300	-0.00045100
C	3.94898600	-0.61369700	1.25895900
H	1.91024300	-1.30062800	1.30259700
H	2.15458600	0.23258100	2.14504300

C	3.94899600	-0.61449000	-1.25871600
H	1.91021000	-1.30134700	-1.30194900
H	2.15462700	0.23134700	-2.14532200
C	4.36833500	-1.37057800	0.00036500
H	4.21911900	-1.17478700	2.15324400
H	4.48239700	0.33977900	1.30467700
H	4.21908200	-1.17618700	-2.15263600
H	4.48246300	0.33892100	-1.30507300
H	5.44674900	-1.52878600	0.00042000
H	3.90048600	-2.35977900	0.00067300
Br	-2.86274100	-1.53528700	0.00005100
H	-3.61204600	2.41184500	0.00027000

I6 (deprotonated)

Electronic energy (E_e)	E_e + ZPV
Hartree	
-3222.525863	-3222.309948

XYZ coordinates

O	0.98389300	2.95589000	-0.00007300
N	-1.20141100	2.32659600	-0.00001600
N	0.55389500	0.72228100	-0.00003100
C	0.11829600	2.07229700	-0.00019800
C	-0.36205500	-0.28218600	0.00001900
C	-1.67852000	-0.02612100	0.00005200
C	-2.15756000	1.36133900	0.00020500
O	-3.36418700	1.62253500	-0.00002300
H	0.02789500	-1.29007600	0.00000600

C	1.98633500	0.39848000	-0.00003000
C	2.39577900	-0.35982600	1.26267900
C	2.39572000	-0.36001500	-1.26264400
H	2.48598000	1.36478300	-0.00010500
C	3.89984500	-0.63419100	1.25922900
H	1.85729900	-1.31184200	1.31083100
H	2.10917400	0.22126700	2.14069900
C	3.89978300	-0.63440300	-1.25922600
H	1.85721500	-1.31202700	-1.31061600
H	2.10908000	0.22094600	-2.14073900
C	4.31977000	-1.39161900	0.00005500
H	4.17695400	-1.19562700	2.15274400
H	4.43643200	0.31850500	1.30090800
H	4.17683700	-1.19599500	-2.15266000
H	4.43637900	0.31828000	-1.30109800
H	5.39854600	-1.55600400	0.00004300
H	3.84631900	-2.37883700	0.00014900
Br	-2.92135700	-1.45815600	0.00000300

I6 homodimer

Electronic energy (E_e)	E_e + ZPV
Hartree	
-6446.054275	-6445.593736

XYZ coordinates

O	3.00961800	-2.27878200	-0.04587400
O	0.67750500	1.59395100	-0.31272200
N	4.20681700	-0.32651900	0.02197600

N	1.88640800	-0.31666400	-0.17817600
C	3.03682000	-1.06679900	-0.06610400
C	1.77038100	1.05540700	-0.21857100
C	3.04161800	1.74549200	-0.13950600
C	4.17817200	1.03567500	-0.02184800
H	1.00920000	-0.84631600	-0.23903800
H	5.13887500	1.52562100	0.04598400
O	-3.00967400	2.27882700	-0.04609100
N	-1.88641000	0.31672100	-0.17816200
N	-4.20683200	0.32654200	0.02188500
C	-3.03685800	1.06684400	-0.06633200
C	-4.17815900	-1.03565200	-0.02185100
C	-3.04159600	-1.74545300	-0.13947500
C	-1.77036600	-1.05535300	-0.21848800
H	-1.00920200	0.84638400	-0.23899200
C	5.49880900	-1.03579600	0.16404100
C	6.39800700	-0.79737800	-1.04630700
C	6.19046800	-0.66354100	1.47268100
H	5.23196100	-2.08972200	0.19792700
C	7.70463300	-1.57659100	-0.88992500
H	6.62346400	0.27016600	-1.13191600
H	5.87217200	-1.09625900	-1.95418000
C	7.49609100	-1.44626500	1.61656100
H	6.41379900	0.40747800	1.47990100
H	5.51996400	-0.86646500	2.30896700
C	8.41144200	-1.21543800	0.41535100
H	8.35282400	-1.37850700	-1.74388000
H	7.48441200	-2.64804500	-0.89621000
H	7.99671000	-1.15792300	2.54113500

H	7.26673100	-2.51280400	1.69564700
H	9.32652000	-1.79947600	0.52103400
H	8.70683900	-0.16188300	0.38555200
O	-0.67748400	-1.59389900	-0.31254500
H	-5.13885400	-1.52561200	0.04597200
C	-5.49884900	1.03578400	0.16388500
C	-6.39819000	0.79682600	-1.04624400
C	-6.19030700	0.66398000	1.47276600
H	-5.23207000	2.08974200	0.19734200
C	-7.70485700	1.57599900	-0.88998800
H	-6.62356700	-0.27077400	-1.13140300
H	-5.87250600	1.09539500	-1.95430800
C	-7.49596400	1.44666300	1.61652400
H	-6.41356900	-0.40705000	1.48044900
H	-5.51970000	0.86728300	2.30887700
C	-8.41146200	1.21531400	0.41552900
H	-8.35315300	1.37752100	-1.74377100
H	-7.48472100	2.64746800	-0.89673900
H	-7.99643600	1.15862900	2.54127500
H	-7.26666800	2.51324600	1.69518400
H	-9.32656300	1.79933500	0.52111400
H	-8.70679700	0.16172900	0.38618500
Br	-3.05097400	-3.62347900	-0.19900300
Br	3.05102300	3.62351300	-0.19908700

I7

Electronic energy (E_e)	E_e + ZPV
Hartree	
-972.4112848	-972.174424

XYZ coordinates

S	-4.49211000	-0.43457600	-0.00003400
O	-0.47627100	2.36492100	0.00027000
C	-2.83266400	-0.29551500	-0.00006300
C	-0.87920500	1.22051800	0.00016200
N	-0.05670900	0.10953800	0.00004400
N	-2.23196600	0.93496400	0.00016700
H	-2.83079700	1.75179800	0.00020300
C	-0.59030300	-1.14564000	-0.00019700
H	0.12996600	-1.95093000	-0.00032500
C	-1.91784200	-1.39081200	-0.00025900
H	-2.29479000	-2.39894900	-0.00046000
C	1.41051900	0.30054100	0.00008900
C	2.04200300	-0.27536000	1.26547400
C	2.04204000	-0.27469800	-1.26556700
H	1.55218500	1.37912300	0.00037000
C	3.54931000	-0.01789200	1.25947900
H	1.85963300	-1.35344700	1.31168500
H	1.57494100	0.17493900	2.14258100
C	3.54934600	-0.01719200	-1.25941000
H	1.85974100	-1.35277300	-1.31233900
H	1.57498100	0.17600700	-2.14246900
C	4.20278300	-0.58446900	-0.00011100
H	3.99844000	-0.45214800	2.15304700

H	3.72720300	1.06051600	1.30424000
H	3.99850100	-0.45095200	-2.15320600
H	3.72722500	1.06124000	-1.30355600
H	5.27111300	-0.36480300	-0.00003100
H	4.10038500	-1.67416900	-0.00042300

I7 (protonated)

Electronic energy (E_e)	E_e + ZPV
Hartree	
-972.8092863	-972.559616

XYZ coordinates

S	4.46668200	-0.47531600	-0.00002900
O	0.42266600	2.33839700	-0.00039600
C	2.83397800	-0.34937200	0.00005500
C	0.90579500	1.12702000	-0.00017300
N	0.05637400	0.11253700	0.00003600
N	2.22122700	0.90833900	-0.00016000
H	2.84289200	1.71236500	-0.00032500
C	0.57222600	-1.17529300	0.00032500
H	-0.16986100	-1.95838700	0.00053400
C	1.89011400	-1.42735100	0.00035000
H	2.25458800	-2.44140400	0.00058100
C	-1.42958800	0.30983200	-0.00003900
C	-2.03899900	-0.27684300	-1.26995600
C	-2.03907000	-0.27629800	1.27009300
H	-1.58674000	1.38487800	-0.00027900
C	-3.55059400	-0.04096600	-1.25944500

H	-1.84101600	-1.35139600	-1.31616300
H	-1.57905800	0.18345400	-2.14519700
C	-3.55066400	-0.04042100	1.25939300
H	-1.84108000	-1.35083100	1.31677300
H	-1.57917800	0.18437500	2.14516100
C	-4.19246100	-0.61969400	0.00008100
H	-3.98905400	-0.48341300	-2.15338900
H	-3.74604400	1.03404700	-1.30555100
H	-3.98917500	-0.48247200	2.15350700
H	-3.74611300	1.03461400	1.30501300
H	-5.26350800	-0.41733500	0.00000700
H	-4.07170400	-1.70702100	0.00032200
H	1.10917600	3.02659400	-0.00059200

I7 (deprotonated)

Electronic energy (E_e)	E_e + ZPV
Hartree	
-971.9153671	-971.691789

XYZ coordinates

S	-4.56596800	-0.08407200	0.00000600
O	0.01394500	-1.92933100	-0.00021300
C	-2.85428000	0.05546800	-0.00000500
C	-0.71580900	-0.93702200	-0.00011700
N	-0.11944600	0.34054600	0.00000300
N	-2.07269900	-1.02387100	-0.00014000
C	-0.90975300	1.44899700	0.00015100
H	-0.38902300	2.39702000	0.00026700

C	-2.25393900	1.36441700	0.00015100
H	-2.86461200	2.25163900	0.00027200
C	1.34509100	0.50416200	0.00004200
C	2.00301200	-0.04440900	1.26610500
C	2.00308900	-0.04398400	-1.26616900
H	1.50025100	1.58644600	0.00021800
C	3.49943900	0.27276900	1.25909800
H	1.85005400	-1.12210800	1.30750000
H	1.52136000	0.39504400	2.14184500
C	3.49951100	0.27321600	-1.25895800
H	1.85013800	-1.12166900	-1.30793400
H	1.52149300	0.39576500	-2.14179000
C	4.17372600	-0.27138200	-0.00000800
H	3.96725400	-0.14376100	2.15234800
H	3.64256900	1.35760900	1.30131400
H	3.96739000	-0.14298400	-2.15232800
H	3.64263000	1.35807300	-1.30077200
H	5.23554500	-0.01812600	0.00006900
H	4.10336800	-1.36325500	-0.00020300

I7 homodimer

Electronic energy (E_e)	E_e + ZPV
Hartree	
-1944.835237	-1944.35918

XYZ coordinates

S	-0.17134600	4.11863700	0.17889600
O	-1.58462800	-0.56091900	-0.14897400
C	-1.37943400	2.97509200	0.09588900

C	-1.97044900	0.59657800	-0.07321700
N	-3.30289700	0.94307100	-0.04699700
N	-1.08207100	1.64307500	-0.00902200
H	-0.09856600	1.35889600	-0.03994100
C	-3.66790700	2.25484600	0.06118400
H	-4.73186300	2.43854000	0.09068800
C	-2.77754000	3.26558100	0.13081200
H	-3.10664600	4.28701000	0.21448800
S	0.17034600	-4.11756200	0.17888600
O	1.58517600	0.56154300	-0.14991500
C	1.37875800	-2.97432900	0.09582500
C	1.97055700	-0.59610100	-0.07388400
N	3.30288800	-0.94304500	-0.04722900
N	1.08183200	-1.64230700	-0.00991700
H	0.09843100	-1.35779000	-0.04152600
C	3.66747900	-2.25488600	0.06170900
H	4.73137500	-2.43885500	0.09169600
C	2.77675800	-3.26528900	0.13154100
H	3.10543000	-4.28681600	0.21571300
C	-4.32998400	-0.12275900	-0.10018600
C	-5.08617800	-0.21686700	1.22265700
C	-5.27026300	0.07303300	-1.28691100
H	-3.77108000	-1.04387000	-0.24937400
C	-6.11294200	-1.34826900	1.16108800
H	-5.60060000	0.72935000	1.41722100
H	-4.37649600	-0.37948700	2.03501400
C	-6.29093700	-1.06484600	-1.33506500
H	-5.80237700	1.02351500	-1.18896800
H	-4.68890400	0.11399500	-2.20916800

C	-7.06357900	-1.16762100	-0.02104600
H	-6.66920500	-1.38962800	2.09798100
H	-5.58755600	-2.30219300	1.05885100
H	-6.97447000	-0.90781100	-2.16967000
H	-5.76862200	-2.00750000	-1.52148300
H	-7.77067300	-1.99689600	-0.06473100
H	-7.64950900	-0.25456700	0.12434700
C	4.33036400	0.12241400	-0.10027100
C	5.08641900	0.21637600	1.22266500
C	5.27077300	-0.07384200	-1.28681700
H	3.77184100	1.04373700	-0.24955600
C	6.11344800	1.34756700	1.16106000
H	5.60066600	-0.72990800	1.41733300
H	4.37670500	0.37922700	2.03494600
C	6.29184500	1.06366000	-1.33501100
H	5.80251700	-1.02450700	-1.18858600
H	4.68957100	-0.11478100	-2.20917000
C	7.06425900	1.16643700	-0.02085400
H	6.66952900	1.38904100	2.09805400
H	5.58826800	2.30156800	1.05848200
H	6.97551500	0.90617900	-2.16941800
H	5.76991400	2.00646000	-1.52175400
H	7.77162700	1.99547700	-0.06454000
H	7.64987100	0.25322000	0.12480700

I8

Electronic energy (E_e)	E_e + ZPV
Hartree	
-1368.983715	-1368.640198

XYZ coordinates

C	1.06788000	0.74923500	1.03450800
H	1.23442000	1.57920900	1.72114100
C	-0.07840000	1.14304700	0.07975100
H	0.23164400	1.28246600	-0.94988800
C	-1.08534700	0.00233600	0.27215100
H	-0.86308400	-0.78003300	-0.44538500
C	-0.77856300	-0.46085700	1.69062000
H	-1.21692900	0.24170700	2.40906100
C	-1.23828000	-1.85115600	2.06121000
H	-0.72834500	-2.16780900	2.96848800
H	-2.31262400	-1.84846200	2.22882900
O	-0.89851800	-2.81047300	1.05972800
C	-1.83643000	-3.07591900	0.12942700
C	-1.32505300	-4.03227000	-0.89982400
H	-2.13443900	-4.32383900	-1.56066200
H	-0.88637000	-4.90424800	-0.41803200
H	-0.54077200	-3.53691100	-1.47327800
O	-2.93331200	-2.57988500	0.14070000
O	0.64361300	-0.39428400	1.74616500
O	-0.61451900	2.35943600	0.60583600
C	-1.38873500	3.09399800	-0.22163800
O	-1.56518000	2.81338900	-1.37617500
C	-1.97411600	4.26939200	0.49503500

H	-1.19239700	4.81482100	1.02093500
H	-2.68736100	3.90718200	1.23653100
H	-2.47722800	4.91614100	-0.21600800
O	-2.43081100	0.41756900	0.17119200
C	-3.02323100	0.24153400	-1.03678800
O	-2.45284600	-0.21761000	-1.98548000
C	-4.44437000	0.70310000	-0.99874700
H	-4.90814100	0.54093200	-1.96596100
H	-4.46917900	1.76342200	-0.74642100
H	-4.98279100	0.15867400	-0.22380000
N	2.35151800	0.47871500	0.41478400
C	2.40778900	-0.41607600	-0.64579700
N	3.67103300	-0.65482400	-1.12663100
H	3.72403000	-1.29872700	-1.90656200
C	4.87526900	-0.12827600	-0.66222300
C	4.72266900	0.77558900	0.46004400
H	5.60179800	1.22554600	0.88958900
C	3.49818300	1.02824600	0.94217500
H	3.33902700	1.69233900	1.77992000
O	1.41795700	-0.93403500	-1.11491100
O	5.92302600	-0.43925500	-1.19267800

I8 (protonated)

Electronic energy (E_e)	E_e + ZPV
Hartree	
-1369.388555	-1369.032396

XYZ coordinates

C	0.97513500	0.79762700	1.08730900
H	1.13892800	1.64110300	1.75780600
C	-0.15738300	1.15864100	0.10565400
H	0.16892200	1.29631300	-0.92046400
C	-1.13702700	-0.01058500	0.28533600
H	-0.88756000	-0.78765600	-0.42851000
C	-0.84108400	-0.46907400	1.70735400
H	-1.31598000	0.20991900	2.42333700
C	-1.24455200	-1.87997600	2.05761300
H	-0.76564100	-2.17147100	2.98984900
H	-2.32490100	-1.92847200	2.17239200
O	-0.80767400	-2.81792100	1.07481900
C	-1.68965800	-3.13598400	0.10393500
C	-1.09472900	-4.09931400	-0.87248200
H	-1.85915700	-4.44009700	-1.56253300
H	-0.64912400	-4.94077600	-0.34512600
H	-0.30266400	-3.58961400	-1.42259600
O	-2.79817900	-2.67243400	0.04750800
O	0.58354000	-0.34452800	1.79381900
O	-0.72799000	2.36081400	0.61246500
C	-1.50007900	3.07392700	-0.24447900
O	-1.61166500	2.78997100	-1.40484400
C	-2.16874000	4.21189500	0.45520500

H	-1.46532500	4.72620800	1.10656500
H	-2.96831300	3.80734900	1.07789800
H	-2.58711800	4.89481800	-0.27687200
O	-2.48738700	0.37031800	0.16267800
C	-3.04946800	0.18686500	-1.06309600
O	-2.43638000	-0.23540200	-2.00144000
C	-4.48592900	0.59485500	-1.05104300
H	-4.92913000	0.40193800	-2.02205100
H	-4.55153900	1.65795700	-0.81741000
H	-5.01591500	0.04524100	-0.27423600
N	2.28502500	0.53232200	0.46884100
C	2.34955400	-0.39966700	-0.56214500
N	3.63950400	-0.59753500	-1.06390000
H	3.69431600	-1.27163200	-1.82479400
C	4.74140200	0.01116400	-0.61023800
C	4.63508400	0.92613300	0.43850000
H	5.50896700	1.42534800	0.82009800
C	3.39093600	1.14698100	0.94117500
H	3.22924000	1.84211000	1.75322900
O	1.40242200	-0.98148000	-1.00790800
O	5.90647800	-0.24063600	-1.13234900
H	5.88419500	-0.87263000	-1.87074500

I8 (deprotonated)

Electronic energy (E_e)	E_e + ZPV
Hartree	
-1368.487439	-1368.157491

XYZ coordinates

C	1.09933300	0.75975200	0.99589300
H	1.24955200	1.59623600	1.68001700
C	-0.06534200	1.13664100	0.05626700
H	0.23181200	1.28565700	-0.97427000
C	-1.05658600	-0.01184300	0.25823200
H	-0.82096900	-0.79423400	-0.45391700
C	-0.74437900	-0.45380100	1.68156900
H	-1.19854000	0.25349800	2.38798900
C	-1.20291100	-1.84130400	2.06635600
H	-0.70670000	-2.14267000	2.98653400
H	-2.28039000	-1.84574000	2.21413900
O	-0.83777100	-2.81152600	1.08508600
C	-1.74871100	-3.09852300	0.13824900
C	-1.18498700	-4.02636400	-0.88972700
H	-1.97260800	-4.35349300	-1.56011200
H	-0.70794600	-4.87741800	-0.40691300
H	-0.41823000	-3.49227500	-1.45248100
O	-2.86445200	-2.64273900	0.13418300
O	0.66969700	-0.37606500	1.74189500
O	-0.61910500	2.34706800	0.59937800
C	-1.41877800	3.07427700	-0.19943500
O	-1.63431800	2.80402900	-1.35143600
C	-1.99381600	4.24385300	0.53963100

H	-1.20229800	4.79378900	1.04570700
H	-2.68317200	3.87358900	1.29949500
H	-2.52302000	4.88987900	-0.15303300
O	-2.41303000	0.38234100	0.16205200
C	-3.00756800	0.20134100	-1.03961600
O	-2.44588300	-0.26002900	-1.99279500
C	-4.43099300	0.66144000	-0.99620100
H	-4.90024300	0.49435700	-1.96004300
H	-4.45415600	1.72313800	-0.74967700
H	-4.96557700	0.12083200	-0.21588800
N	2.37008000	0.50047900	0.38185000
C	2.46822000	-0.39274100	-0.72456500
N	3.67477400	-0.66079900	-1.23733000
C	4.81915100	-0.13956200	-0.71652700
C	4.71733400	0.74133700	0.45054800
H	5.61309300	1.15500100	0.88614700
C	3.50657000	1.01842900	0.94827900
H	3.35313800	1.65952600	1.80636900
O	1.40538200	-0.85558800	-1.15035500
O	5.93111200	-0.39436800	-1.20368400

I8 homodimer

Electronic energy (E_e)	E_e + ZPV
Hartree	
-2737.980679	-2737.292

XYZ coordinates

C	-5.16912300	-1.96192600	-0.26629400

H	-5.41186000	-3.00833300	-0.08081700
C	-5.82510200	-1.11692400	0.84465300
H	-5.11999800	-0.67780100	1.54108400
C	-6.63482200	-0.08688800	0.05133000
H	-6.00251000	0.77171200	-0.14147900
C	-6.93791000	-0.83886100	-1.23776900
H	-7.75703900	-1.54741900	-1.06793500
C	-7.30114900	0.01154700	-2.43076200
H	-7.27418000	-0.59484900	-3.33334600
H	-8.30073200	0.41851700	-2.29467000
O	-6.36114900	1.06647000	-2.63238200
C	-6.64766100	2.25828000	-2.07348300
C	-5.53328800	3.23345000	-2.27882700
H	-5.84592800	4.21958700	-1.95227500
H	-5.23399400	3.24915200	-3.32524700
H	-4.67512100	2.90492400	-1.69064100
O	-7.66955500	2.47644000	-1.47475100
O	-5.72486700	-1.54099100	-1.49472600
O	-6.71208900	-2.00786200	1.52758000
C	-7.13858200	-1.62724600	2.75111200
O	-6.74113500	-0.63935700	3.30653200
C	-8.14652800	-2.59161500	3.29141100
H	-7.76408400	-3.60873000	3.22503300
H	-9.05011000	-2.53450600	2.68352300
H	-8.37796300	-2.33933000	4.32098700
O	-7.83645700	0.29994000	0.68356700
C	-7.78077000	1.43474900	1.42529400
O	-6.77462300	2.06667700	1.58018300
C	-9.12576900	1.76529900	1.98822500

H	-9.03421900	2.58178100	2.69660600
H	-9.55335200	0.88843900	2.47213000
H	-9.78563900	2.05612800	1.17027200
N	-3.72022500	-1.89892900	-0.35157100
C	-3.10105300	-0.65414100	-0.40554900
N	-1.73956800	-0.69177500	-0.57264700
H	-1.26580700	0.21930500	-0.61916000
C	-0.94607700	-1.81663500	-0.68950900
C	-1.66120000	-3.07052400	-0.64707700
H	-1.11262000	-3.99211200	-0.74458300
C	-2.99348300	-3.05650900	-0.49215800
H	-3.57545300	-3.96670600	-0.46527700
O	-3.71907300	0.38230100	-0.30134700
O	0.26784200	-1.70603500	-0.82064000
C	5.17024300	1.97494000	-0.13857500
H	5.40846100	3.00517800	0.12570300
C	5.81393100	1.05222900	0.91666800
H	5.10230000	0.56214400	1.57165600
C	6.63631000	0.08341000	0.06161100
H	6.01036400	-0.76173400	-0.19899600
C	6.95279700	0.92573200	-1.16700300
H	7.77045100	1.61899100	-0.93694300
C	7.33013200	0.16298700	-2.41386800
H	7.31589800	0.83340900	-3.27019600
H	8.32704100	-0.25541700	-2.29436100
O	6.39178500	-0.87251000	-2.70397900
C	6.66603400	-2.09985500	-2.22107200
C	5.55400400	-3.05551500	-2.51227200
H	5.85048800	-4.05968000	-2.22823000

H	5.29027400	-3.01500000	-3.56768500
H	4.67834900	-2.75178000	-1.93663200
O	7.67678700	-2.36003600	-1.62004500
O	5.74325000	1.64591800	-1.38695900
O	6.69120900	1.89294200	1.67185200
C	7.10968900	1.42283400	2.86657800
O	6.70551400	0.39849700	3.34626900
C	8.11803600	2.34136300	3.48099800
H	7.73534300	3.36041000	3.49665100
H	9.02075700	2.33320700	2.86919100
H	8.35050400	2.00774300	4.48680000
O	7.83298400	-0.34454000	0.67698700
C	7.77174200	-1.52719100	1.33990600
O	6.76315800	-2.16474600	1.44806200
C	9.11120100	-1.89238700	1.89460100
H	9.03758800	-2.82809800	2.43834400
H	9.45927300	-1.09983100	2.55675000
H	9.82572200	-1.98761200	1.07734500
N	3.72265600	1.91662600	-0.24644200
C	3.10508300	0.67831700	-0.39016400
N	1.74302200	0.72606800	-0.55055400
H	1.26933900	-0.18036700	-0.65181200
C	0.94861900	1.85526600	-0.59049600
C	1.66315600	3.10413300	-0.46630600
H	1.11325500	4.02940600	-0.50090000
C	2.99512100	3.08053800	-0.30928700
H	3.57568900	3.98755300	-0.21755500
O	3.72429400	-0.36232200	-0.36367600
O	-0.26577300	1.75229400	-0.72456500

I9

Electronic energy (E_e)	E_e + ZPV
Hartree	
-1219.723739	-1219.36625

XYZ coordinates

N	1.62407700	-0.78728600	-0.42866100
H	4.49705400	-1.55408100	-1.68614900
C	2.51903200	-1.32495200	-1.34210900
N	3.83865000	-1.16340800	-1.02432800
C	4.36667400	-0.54638500	0.09891800
C	3.37852600	-0.03405500	1.03146500
C	2.05939200	-0.18171600	0.75610000
O	2.17722000	-1.90867400	-2.35456300
C	3.89077900	0.64735900	2.26937900
H	4.96633700	0.77847200	2.18445200
H	3.43546700	1.62769800	2.40550100
H	3.69544700	0.05929800	3.16794000
C	0.21674500	-0.88840100	-0.81014900
H	0.22585700	-1.16250800	-1.86024800
C	-0.63490900	-1.89825400	-0.01763800
H	-0.87827800	-2.75182100	-0.64616000
H	-0.12137400	-2.26359000	0.86724300
C	-1.90675200	-1.12153300	0.32733600
H	-1.97429600	-0.89191800	1.38857200
C	-1.78534900	0.16677100	-0.50488900
H	-2.24347000	-0.00840000	-1.48561000
C	-2.39117100	1.38913100	0.14339700
H	-3.46745600	1.27056700	0.23425800

H	-1.95558500	1.55785800	1.12644500
O	-0.39212300	0.37784700	-0.65551900
O	-2.17394600	2.52751300	-0.69485000
C	-1.16209800	3.35691400	-0.38271400
O	-0.48080000	3.24075300	0.60229600
C	-1.02431200	4.44013200	-1.40683500
H	-0.86286100	3.99423800	-2.38754900
H	-1.94789600	5.01647900	-1.45114200
H	-0.19270200	5.08601700	-1.14590100
O	-3.05281600	-1.88595200	-0.05221000
C	-4.20937500	-1.62951300	0.58772400
O	-4.31634700	-0.78285000	1.43678500
C	-5.30703700	-2.52182200	0.10146500
H	-5.01816500	-3.56344400	0.23531400
H	-6.21861900	-2.31004500	0.65016400
H	-5.46367500	-2.35379400	-0.96373600
C	1.03282800	0.32472900	1.72499800
H	0.68690900	1.31590500	1.42990800
H	0.16575600	-0.32416500	1.78569700
H	1.47312100	0.38609200	2.71509400
O	5.57649800	-0.48419100	0.23657800

I9 (protonated)

Electronic energy (E_e)	E_e + ZPV
Hartree	
-1220.13633	-1219.765936

XYZ coordinates

N	1.56068800	-0.88053200	-0.39044400
H	4.46801200	-1.64024200	-1.59278600
C	2.47242900	-1.45551900	-1.27207300
N	3.80013200	-1.22977500	-0.94535900
C	4.21419800	-0.54454000	0.12963300
C	3.29176200	-0.01308500	1.02544500
C	1.95279800	-0.22293400	0.74322600
O	2.17241600	-2.10222800	-2.24243400
C	3.75549000	0.76242100	2.22600400
H	4.81840000	0.97242000	2.15554500
H	3.22622000	1.71136700	2.29442500
H	3.58142300	0.20757500	3.14820000
C	0.14479500	-0.96435800	-0.83113700
H	0.19950700	-1.25910600	-1.87420700
C	-0.74519200	-1.93704300	-0.04179900
H	-1.03182900	-2.77229800	-0.67567200
H	-0.24498300	-2.33350100	0.83744200
C	-1.97903200	-1.10395400	0.31900100
H	-2.02802000	-0.88440600	1.38353500
C	-1.81129600	0.18640500	-0.50186700
H	-2.31358300	0.05451800	-1.46589000
C	-2.30974400	1.43793600	0.18126500
H	-3.38215200	1.37091600	0.34030500

H	-1.80765400	1.58456800	1.13613500
O	-0.40851100	0.31829000	-0.71329000
O	-2.09379700	2.56476800	-0.67205200
C	-1.01885000	3.33846600	-0.44563800
O	-0.25147500	3.16755400	0.46698200
C	-0.91945700	4.42880900	-1.46552300
H	-0.79070700	3.98520600	-2.45269300
H	-1.84356600	5.00464600	-1.47905000
H	-0.07765700	5.07203300	-1.23237300
O	-3.15484700	-1.81782100	-0.05548600
C	-4.29797900	-1.50251700	0.59097400
O	-4.35619600	-0.63658700	1.42361800
C	-5.43492100	-2.35673600	0.13228800
H	-5.19328400	-3.40576500	0.29931100
H	-6.33355600	-2.08695300	0.67673800
H	-5.58679000	-2.21497800	-0.93731900
C	0.93185600	0.28938300	1.69929000
H	0.64193100	1.30315200	1.41188800
H	0.04052500	-0.32463900	1.72213200
H	1.35896500	0.31633700	2.69718700
O	5.49694600	-0.38993500	0.33964900
H	6.05563400	-0.77742400	-0.35266700

I9 (deprotonated)

Electronic energy (E_e)	E_e + ZPV
Hartree	
-1219.222145	-1218.878925

XYZ coordinates

N	1.64909400	-0.75436500	-0.43658500
C	2.57022900	-1.33733100	-1.35392900
N	3.88026300	-1.24623800	-1.11162500
C	4.36630300	-0.63437200	-0.00240500
C	3.42487700	-0.07996400	0.98332900
C	2.09801800	-0.17384800	0.74437300
O	2.10091700	-1.92089400	-2.34320200
C	3.98607500	0.58420200	2.21133100
H	5.05316700	0.73368000	2.06806300
H	3.52485000	1.55397600	2.40144700
H	3.85676300	-0.02469000	3.11025600
C	0.25123100	-0.84721100	-0.78622800
H	0.23435400	-1.11276700	-1.83774000
C	-0.59750300	-1.86162200	0.01095000
H	-0.81969300	-2.72813400	-0.60830200
H	-0.08462100	-2.20548700	0.90490600
C	-1.88243800	-1.10213100	0.33686600
H	-1.96199400	-0.85946000	1.39445900
C	-1.76788900	0.17775500	-0.50863200
H	-2.19337300	-0.02361600	-1.50046100
C	-2.42425400	1.39444500	0.10151600
H	-3.50378600	1.27431600	0.12769600
H	-2.04797500	1.56923700	1.10740800

O	-0.38181300	0.41967000	-0.61415900
O	-2.15376100	2.52806900	-0.72927600
C	-1.16424300	3.35789900	-0.35295300
O	-0.61233300	3.30235000	0.71453900
C	-0.85407200	4.34592000	-1.43411500
H	-0.35443200	3.81797000	-2.24730700
H	-1.77179300	4.77793200	-1.82863900
H	-0.19948900	5.11993800	-1.04737000
O	-3.02036000	-1.88573000	-0.04112600
C	-4.18370100	-1.63408000	0.57921900
O	-4.31725000	-0.77390400	1.41255200
C	-5.26442900	-2.55146200	0.09763400
H	-4.95895700	-3.58640900	0.24509900
H	-6.18339300	-2.34892700	0.63760500
H	-5.41713200	-2.39884100	-0.97047200
C	1.09753000	0.36097900	1.73313600
H	0.77423400	1.36541200	1.45814800
H	0.20810400	-0.25796100	1.80168500
H	1.55082500	0.39737300	2.71959600
O	5.59140600	-0.55136000	0.19327800

I9 homodimer

Electronic energy (E_e)	E_e + ZPV
Hartree	
-2439.460967	-2438.745335

XYZ coordinates

N	-4.20677700	-0.53982900	-0.05114500

H	-1.03912100	-0.72653300	-0.77000700
C	-3.07481700	-0.58290600	-0.85774000
N	-1.88622300	-0.68288400	-0.19090200
C	-1.71337100	-0.75762200	1.17174000
C	-2.92439100	-0.75235900	1.96331600
C	-4.12429300	-0.67023300	1.33514600
O	-3.12378900	-0.54474300	-2.07320100
O	-0.57714700	-0.83909100	1.63565200
C	-2.78420200	-0.84017600	3.45677500
H	-1.74004500	-0.70602900	3.72633400
H	-3.37036100	-0.07108000	3.95840100
H	-3.10592600	-1.81060100	3.83928500
C	-5.46477100	-0.33218000	-0.76351900
H	-5.17919900	0.00268600	-1.75584000
C	-6.39482100	-1.55449400	-0.87439100
H	-6.39550800	-1.92751700	-1.89586400
H	-6.08784100	-2.36328700	-0.21725300
C	-7.77577200	-1.00331400	-0.51468000
H	-8.13085900	-1.37122200	0.44574700
C	-7.55961000	0.51951000	-0.47109600
H	-7.73103200	0.92402400	-1.47591000
C	-8.41278500	1.25202700	0.53751100
H	-9.46040300	1.19579000	0.25529100
H	-8.27773000	0.83481800	1.53351900
O	-6.19824500	0.68571000	-0.11229200
O	-8.05854300	2.63793500	0.52762200
C	-7.23237800	3.08512500	1.48977100
O	-6.86362100	2.41629400	2.41993600
C	-6.84388200	4.50937800	1.24221400

H	-6.17014000	4.53947700	0.38504300
H	-7.72222000	5.10402100	0.99874400
H	-6.34046800	4.90932200	2.11610900
O	-8.71484200	-1.37444500	-1.52577500
C	-10.01547500	-1.41227400	-1.18122700
O	-10.41241800	-1.11855000	-0.08344500
C	-10.86508200	-1.87173800	-2.32349200
H	-10.61662100	-2.90716600	-2.55714400
H	-11.91296500	-1.79537000	-2.05346100
H	-10.65369500	-1.27201500	-3.20732500
C	-5.39038800	-0.70468000	2.13858100
H	-5.72191000	0.30815600	2.37119200
H	-6.19985200	-1.20300400	1.61679200
H	-5.20968800	-1.23821400	3.06679600
N	4.20441800	-0.53708500	0.05204300
H	1.03677900	-0.72357600	0.77091700
C	3.07242700	-0.57892000	0.85857200
N	1.88388100	-0.67998600	0.19180700
C	1.71122300	-0.75701800	-1.17074300
C	2.92235100	-0.75309200	-1.96217400
C	4.12220000	-0.67054500	-1.33396700
O	3.12140200	-0.53892400	2.07399100
O	0.57509800	-0.83955500	-1.63469800
C	2.78225100	-0.84329200	-3.45549200
H	1.73864000	-0.70585600	-3.72555400
H	3.37129300	-0.07722700	-3.95832100
H	3.10048100	-1.81559900	-3.83616200
C	5.46228400	-0.32834100	0.76441400
H	5.17653000	0.01069500	1.75525900

C	6.39035800	-1.55178900	0.88041400
H	6.39046000	-1.92056700	1.90343500
H	6.08181400	-2.36265700	0.22652200
C	7.77202200	-1.00439800	0.51806200
H	8.12611500	-1.37673700	-0.44094100
C	7.55854900	0.51834100	0.46872800
H	7.73116200	0.92647800	1.47185700
C	8.41436500	1.24362900	-0.54252000
H	9.46215600	1.18089900	-0.26168200
H	8.27539900	0.82515800	-1.53745400
O	6.19754900	0.68612900	0.10971200
O	8.06917800	2.63176700	-0.53496300
C	7.24271100	3.08161400	-1.49579800
O	6.86775300	2.41289500	-2.42348200
C	6.86238100	4.50831400	-1.24979600
H	6.18915500	4.54295100	-0.39236900
H	7.74395100	5.09850000	-1.00729200
H	6.36063800	4.91000100	-2.12384600
O	8.71198100	-1.37082400	1.52988000
C	10.01137900	-1.42266500	1.18178400
O	10.40647600	-1.15419800	0.07691400
C	10.86324100	-1.84816500	2.33548500
H	10.52463600	-2.81631500	2.70213400
H	11.90030100	-1.90439100	2.02226300
H	10.75490100	-1.12870800	3.14676800
C	5.38852900	-0.70867500	-2.13690600
H	5.72166000	0.30302400	-2.37214900
H	6.19695700	-1.20694100	-1.61336700
H	5.20739200	-1.24448200	-3.06373300

I10

Electronic energy (E_e)	E_e + ZPV
Hartree	
-1542.676711	-1542.321616

XYZ coordinates

N	-1.34404400	-0.81990200	0.40088900
H	-4.21123800	-1.62860600	1.62798600
C	-2.23857800	-1.40234500	1.28497700
N	-3.55861900	-1.18918200	0.99078700
C	-4.07582100	-0.46788600	-0.04972600
C	-3.10797400	0.11264500	-0.93529300
C	-1.77918100	-0.12337600	-0.72170500
O	-1.90226000	-2.06619000	2.24679800
C	-3.59024100	0.94025100	-2.09238200
H	-4.48623400	1.48218500	-1.79999800
H	-2.84389200	1.66437000	-2.40629300
H	-3.85632100	0.31838600	-2.95021300
C	0.06491400	-0.92634500	0.79364900
H	0.04399400	-1.21928200	1.83876500
C	0.92785400	-1.91796100	-0.00822000
H	1.18052700	-2.77465200	0.61219400
H	0.41958300	-2.28003900	-0.89748800
C	2.19087000	-1.12132100	-0.34328400
H	2.25120200	-0.87272600	-1.40065600
C	2.05853100	0.15143000	0.51014100
H	2.51892400	-0.03433200	1.48757000
C	2.64953300	1.39106700	-0.11896100
H	3.72715500	1.28629000	-0.21074100

H	2.21292100	1.56943800	-1.09989300
O	0.66224000	0.34567600	0.66626100
O	2.41885600	2.51397100	0.73619200
C	1.39984900	3.33851800	0.43432600
O	0.71805200	3.22685300	-0.55095400
C	1.25498900	4.41015200	1.46934900
H	1.09661500	3.95367700	2.44567300
H	2.17491700	4.99190600	1.51897900
H	0.41906300	5.05303900	1.21485500
O	3.34550400	-1.87983900	0.01878700
C	4.49723500	-1.59779600	-0.62053400
O	4.59046200	-0.73315500	-1.45261000
C	5.60686300	-2.48641400	-0.15603500
H	5.33098200	-3.52855600	-0.31163200
H	6.51395100	-2.25132300	-0.70266400
H	5.76435900	-2.33902900	0.91211600
C	-0.75428700	0.34445700	-1.71183000
H	-0.40001000	1.34445700	-1.45833100
H	0.10487800	-0.31473000	-1.74800400
H	-1.19606700	0.36422900	-2.70314000
S	-5.73355200	-0.36563100	-0.21459100

I10 (protonated)

Electronic energy (E_e)	E_e + ZPV
Hartree	
-1543.075065	-1542.707704

XYZ coordinates

N	1.30443000	-0.96387500	-0.30062500
H	4.19553600	-1.87385300	-1.37341900
C	2.22467600	-1.58209800	-1.02947600
N	3.51269400	-1.36188600	-0.81987500
C	4.02417800	-0.44426000	0.09992600
C	3.02540900	0.18444500	0.92281000
C	1.71165800	-0.13339900	0.76714600
O	1.81944200	-2.42517200	-1.94217100
C	3.48478900	1.14533200	1.97584200
H	4.29416900	1.75366500	1.57828300
H	2.68442000	1.80465300	2.29502200
H	3.87785000	0.61312100	2.84449400
C	-0.11740500	-1.04633600	-0.75897000
H	-0.06582500	-1.38627700	-1.78868000
C	-1.03444500	-1.95684000	0.06933600
H	-1.33334400	-2.81732100	-0.52409500
H	-0.55053700	-2.31800500	0.97221500
C	-2.25285100	-1.07750800	0.37133100
H	-2.31792000	-0.81397800	1.42449300
C	-2.03802700	0.17366200	-0.49700900
H	-2.53640500	0.02185200	-1.45953900
C	-2.50570800	1.46198800	0.13583100
H	-3.57843700	1.41816900	0.30258400

H	-1.99736400	1.63715300	1.08218300
O	-0.62767200	0.25225000	-0.70597300
O	-2.27507000	2.54998600	-0.76178500
C	-1.20105100	3.33154900	-0.54973400
O	-0.43809200	3.18097100	0.36931300
C	-1.10079400	4.40040200	-1.59164100
H	-0.98595100	3.93959100	-2.57241500
H	-2.01967300	4.98481600	-1.60498800
H	-0.25122800	5.03990400	-1.37709700
O	-3.43795100	-1.77392200	-0.00403100
C	-4.58528200	-1.41515000	0.61493600
O	-4.63425200	-0.53306100	1.43016800
C	-5.73654300	-2.24379700	0.14566000
H	-5.52266700	-3.29802200	0.31682400
H	-6.63493100	-1.95072700	0.67825500
H	-5.87206400	-2.09955600	-0.92591800
C	0.67160600	0.34107400	1.73004300
H	0.30890300	1.33074500	1.45052200
H	-0.17134500	-0.33732400	1.78038200
H	1.11316100	0.39093000	2.72193100
S	5.65185200	-0.23954900	0.18725400
H	2.54164200	-2.76922000	-2.49397400

I10 (deprotonated)

Electronic energy (E_e)	E_e + ZPV
Hartree	
-1542.181632	-1541.840559

XYZ coordinates

N	1.36966700	-0.79116200	-0.40387000
C	2.28222900	-1.41226000	-1.28792700
N	3.60160000	-1.26213100	-1.06664900
C	4.06850200	-0.54866700	-0.04092100
C	3.15312700	0.08216800	0.89409800
C	1.81771100	-0.10320200	0.71035300
O	1.82997500	-2.08384600	-2.22135400
C	3.67787300	0.90579800	2.03736200
H	4.55560500	1.45760200	1.71025900
H	2.93998100	1.61959000	2.39617900
H	3.99522700	0.28440200	2.87891400
C	-0.03184000	-0.89117200	-0.76679100
H	-0.03611800	-1.18091700	-1.81237900
C	-0.88857500	-1.88493900	0.04464800
H	-1.11746700	-2.75939600	-0.56051300
H	-0.38066600	-2.21765700	0.94557100
C	-2.16755400	-1.10678300	0.35491100
H	-2.24453800	-0.84435700	1.40796100
C	-2.04217700	0.15654400	-0.51338300
H	-2.47283900	-0.05619600	-1.50014300
C	-2.67951500	1.39224900	0.07876400
H	-3.76006000	1.28449100	0.11483000
H	-2.29396400	1.58001600	1.07879500

O	-0.65226200	0.38104000	-0.62913200
O	-2.40164400	2.50740500	-0.77378300
C	-1.41765300	3.34823100	-0.40684600
O	-0.86888000	3.31091700	0.66311000
C	-1.10994300	4.32258200	-1.50087100
H	-0.61442100	3.78532700	-2.31037600
H	-2.02890500	4.75129200	-1.89621600
H	-0.45324200	5.10024600	-1.12527800
O	-3.31070900	-1.88770100	-0.00910900
C	-4.47247700	-1.61245200	0.60621200
O	-4.59707200	-0.73211800	1.41914600
C	-5.55967300	-2.53514700	0.15136200
H	-5.28149700	-3.56365600	0.37912800
H	-6.48791100	-2.27961000	0.65144700
H	-5.67883100	-2.45457700	-0.92853000
C	0.81090900	0.39871100	1.71120100
H	0.47442800	1.40615600	1.46535400
H	-0.06676400	-0.23660900	1.75874600
H	1.25991500	0.40775600	2.69997800
S	5.76576600	-0.42164900	0.18224100

I10 homodimer

Electronic energy (E_e)	E_e + ZPV
Hartree	
-3085.366567	-3084.654225

XYZ coordinates

N	-3.30546900	-0.94027000	-0.51289700

H	-0.10554800	-1.36663300	-0.55057300
C	-1.96706700	-0.60092100	-0.52730500
N	-1.09113800	-1.64317000	-0.53405900
C	-1.40049000	-2.97541700	-0.51853000
C	-2.80236100	-3.28569000	-0.51808700
C	-3.71234700	-2.26921200	-0.56825800
O	-1.57989300	0.56081900	-0.54455800
C	-3.23481000	-4.72409900	-0.49797400
H	-2.50215600	-5.31536400	0.04447500
H	-4.19898500	-4.84501000	-0.01126800
H	-3.30021800	-5.13789500	-1.50698400
C	-4.23332600	0.18357200	-0.34574100
H	-3.61719600	1.01355300	-0.01380500
C	-5.03855100	0.59761800	-1.58988400
H	-4.66636500	1.54477600	-1.97265700
H	-4.97318400	-0.13982200	-2.38482000
C	-6.46804900	0.76490800	-1.06891300
H	-7.14357500	0.00574900	-1.45892500
C	-6.32262100	0.63852500	0.45913700
H	-6.16260600	1.63903000	0.87726500
C	-7.48033400	-0.04011000	1.15399400
H	-8.37891100	0.56409500	1.07283200
H	-7.65913900	-1.02525000	0.72763700
O	-5.16336300	-0.15564500	0.65856100
O	-7.18126400	-0.15102400	2.54870100
C	-6.75469700	-1.33869500	3.01250700
O	-6.72022200	-2.34321400	2.34973300
C	-6.33785900	-1.23474900	4.44619900
H	-5.41392900	-0.65759100	4.49698500

H	-7.09553000	-0.70522900	5.02060400
H	-6.17294200	-2.22670200	4.85349400
O	-6.95641000	2.05342100	-1.44707300
C	-8.29095600	2.22564500	-1.47575600
O	-9.06977300	1.35755500	-1.17759900
C	-8.64945000	3.60838000	-1.91871600
H	-8.22352400	3.79696200	-2.90342900
H	-9.72858700	3.71390200	-1.94831500
H	-8.22054100	4.33265300	-1.22676400
C	-5.17631700	-2.56396200	-0.70583300
H	-5.63700800	-2.69064600	0.27495400
H	-5.70511900	-1.77168500	-1.22162500
H	-5.30303800	-3.47832800	-1.27775800
S	-0.15889400	-4.09188700	-0.54282000
N	3.30559800	0.94108600	-0.51338700
H	0.10565700	1.36763400	-0.55391200
C	1.96719100	0.60168300	-0.52965000
N	1.09128400	1.64390400	-0.53585600
C	1.40061600	2.97617000	-0.51851400
C	2.80244000	3.28648500	-0.51648000
C	3.71248000	2.27006500	-0.56700200
O	1.58020600	-0.56010300	-0.54901600
C	3.23499900	4.72483600	-0.49440000
H	2.50151500	5.31569800	0.04735100
H	4.19839200	4.84517500	-0.00599700
H	3.30217600	5.13949600	-1.50294300
C	4.23333400	-0.18294700	-0.34699100
H	3.61703400	-1.01331000	-0.01634500
C	5.03921200	-0.59559000	-1.59119500

H	4.66709800	-1.54223300	-1.97530200
H	4.97442600	0.14285000	-2.38525500
C	6.46838800	-0.76371500	-1.06961000
H	7.14426300	-0.00429300	-1.45851400
C	6.32219100	-0.63892700	0.45848700
H	6.16189700	-1.63986000	0.87547900
C	7.47966400	0.03883300	1.15456700
H	8.37829800	-0.56522000	1.07276700
H	7.65849900	1.02454300	0.72953700
O	5.16285900	0.15510000	0.65820100
O	7.18035000	0.14787800	2.54936000
C	6.75400800	1.33501300	3.01480700
O	6.71926400	2.34033500	2.35329500
C	6.33816200	1.22928600	4.44866400
H	5.41584800	0.64961900	4.49992000
H	7.09773500	0.70153400	5.02222700
H	6.17104000	2.22062300	4.85654900
O	6.95668600	-2.05193700	-1.44884000
C	8.29120700	-2.22433800	-1.47737900
O	9.07010200	-1.35667300	-1.17818800
C	8.64958100	-3.60666800	-1.92170900
H	8.22399300	-3.79406200	-2.90680000
H	9.72870300	-3.71238500	-1.95101500
H	8.22024600	-4.33160800	-1.23072600
C	5.17651900	2.56514800	-0.70286900
H	5.63617100	2.69096300	0.27852100
H	5.70599900	1.77342000	-1.21876800
H	5.30364000	3.48010500	-1.27375800
S	0.15893500	4.09257800	-0.54261500

I11

Electronic energy (E_e)	E_e + ZPV
Hartree	
-1981.201943	-1980.699161

XYZ coordinates

N	1.23875900	2.66065100	0.38140600
C	0.97365800	3.94663300	0.81090900
N	1.49349300	4.92724400	-0.02639500
C	2.42336200	4.71055900	-1.02437200
C	2.61935600	3.25003400	-1.35678000
O	0.41497800	4.20842600	1.84866200
C	0.48477900	1.53934800	0.90168000
H	0.16093200	1.83388400	1.89640400
C	-0.70830500	1.16772200	0.01289700
H	-1.06247000	2.01129000	-0.56850100
C	-0.16307100	0.02044200	-0.86022100
H	0.13854600	0.41738100	-1.82843600
C	1.02326200	-0.53273200	-0.06304400
H	0.73612300	-1.47543000	0.40546100
C	2.23516700	-0.74609100	-0.93470500
H	1.98656100	-1.41364100	-1.76160800
H	2.59427800	0.20146000	-1.33627300
O	1.30549300	0.40421300	0.97664300
O	3.24707400	-1.34269400	-0.12192100
C	4.41455900	-1.58176300	-0.72977100
O	4.61170500	-1.32851700	-1.89250800
O	-1.08953500	-1.04341200	-1.03972000
C	-2.27711600	-0.71247200	-1.57836400
O	-2.50291200	0.38129200	-2.03042800

O	1.45199800	2.53020000	-0.99955000
O	3.01833000	5.61034100	-1.56083600
C	-3.26904100	-1.81550900	-1.50344800
C	-2.92201600	-3.09090400	-1.06398000
C	-4.59265500	-1.51488300	-1.81597100
C	-3.90261900	-4.06494000	-0.94225300
H	-1.89486300	-3.31664900	-0.81573100
C	-5.57062900	-2.48798700	-1.68126300
H	-4.84383500	-0.51472100	-2.14104200
C	-5.22567200	-3.76241800	-1.24419300
H	-3.63561100	-5.05739700	-0.60644100
H	-6.60082800	-2.25242900	-1.91024800
H	-5.98931900	-4.52122700	-1.13714400
C	5.42299100	-2.18996600	0.17983800
C	6.64869900	-2.56868900	-0.36188600
C	5.16521000	-2.38269700	1.53535500
C	7.61425600	-3.14360600	0.44980900
H	6.83066100	-2.40998400	-1.41569800
C	6.13594600	-2.95519600	2.34458600
H	4.21431800	-2.08252900	1.95107900
C	7.35780600	-3.33643800	1.80303400
H	8.56501200	-3.44181300	0.03011900
H	5.93909200	-3.10356100	3.39728700
H	8.11167700	-3.78376300	2.43682100
O	-1.75777400	0.64308800	0.80978400
C	-2.91460700	1.34637200	0.84630500
O	-2.99483700	2.49478800	0.50617600
C	-4.05342300	0.51801200	1.32046200
C	-3.89402200	-0.82426500	1.66168500

C	-5.31714200	1.10313500	1.34466900
C	-5.00178300	-1.57791500	2.02210600
H	-2.91313600	-1.27640800	1.63097900
C	-6.42105500	0.34573800	1.70377200
H	-5.42265300	2.14350800	1.07052100
C	-6.26347000	-0.99455500	2.04015900
H	-4.88127300	-2.62070600	2.28146800
H	-7.40324300	0.79725300	1.71841700
H	-7.12666500	-1.58576900	2.31548200
H	2.74616200	3.13438800	-2.42983300
H	3.49617500	2.86832100	-0.82680400
H	1.35787000	5.88166700	0.28612500

I11 (protonated)

Electronic energy (E_e)	E_e + ZPV
Hartree	
-1981.598212	-1981.081481

XYZ coordinates

N	1.10256700	1.72021200	0.81441900
H	2.25376900	4.22911200	2.42172500
C	1.16480900	2.67104000	1.71830500
N	2.26410300	3.42733800	1.79605300
C	3.50933500	2.99954600	1.27261700
C	3.38367200	1.74248000	0.44520700
O	0.21657500	2.91921900	2.57176900
C	0.22071300	0.54130800	0.77707000
H	-0.41181500	0.59971400	1.66507300

C	-0.61041500	0.49460700	-0.51069000
H	-0.14040600	1.07453600	-1.30358500
C	-0.57930500	-0.99931000	-0.86422900
H	-0.73106700	-1.18614300	-1.92395500
C	0.79650600	-1.40448800	-0.36183300
H	0.81158000	-2.43976300	-0.02960200
C	1.86738700	-1.16407100	-1.40470600
H	1.74960200	-1.87888500	-2.21996000
H	1.82365700	-0.15733300	-1.81711700
O	1.01452000	-0.59329700	0.81242200
O	3.12113400	-1.36837900	-0.75716800
C	4.20661800	-1.02732800	-1.47125700
O	4.13511000	-0.55382100	-2.57659600
O	-1.54291500	-1.69329500	-0.08108400
C	-2.79988000	-1.72895200	-0.58199300
O	-3.08948400	-1.22966500	-1.63706900
O	2.08292200	1.73046800	-0.15073000
O	4.51191200	3.59332700	1.51371000
C	-3.75006200	-2.44001300	0.30767600
C	-3.32531800	-3.13632100	1.43774100
C	-5.10275500	-2.39252100	-0.02475900
C	-4.25785200	-3.79087300	2.22901600
H	-2.27506900	-3.17287500	1.68876700
C	-6.03041000	-3.04144300	0.77445200
H	-5.41063900	-1.84516000	-0.90514700
C	-5.60748000	-3.74198300	1.89903900
H	-3.93396400	-4.34165300	3.10112300
H	-7.08069000	-3.00516100	0.52055000
H	-6.33171700	-4.25293300	2.51894800

C	5.47674200	-1.28850900	-0.74440400
C	6.67245300	-0.97060200	-1.38479200
C	5.48997000	-1.83827700	0.53620500
C	7.87991600	-1.19991100	-0.74358700
H	6.64256900	-0.55119800	-2.38079200
C	6.70122100	-2.06488000	1.17380000
H	4.56071400	-2.09156700	1.02590100
C	7.89409000	-1.74691600	0.53469300
H	8.80853700	-0.95677600	-1.24078900
H	6.71565900	-2.49318700	2.16629400
H	8.83687100	-1.92702900	1.03339800
O	-1.89723900	0.98685000	-0.21931000
C	-2.60156700	1.50659600	-1.26722900
O	-2.09803500	1.72071600	-2.33310600
C	-4.01274700	1.77635500	-0.90703600
C	-4.56906100	1.30449300	0.28076700
C	-4.79313000	2.48725000	-1.81573200
C	-5.90625100	1.54877600	0.55728200
H	-3.96600200	0.73797000	0.97587000
C	-6.12652500	2.73538100	-1.53058800
H	-4.34857200	2.83635900	-2.73735100
C	-6.68285800	2.26519400	-0.34574500
H	-6.34266200	1.17456100	1.47309300
H	-6.73373600	3.29016800	-2.23200400
H	-7.72515400	2.45516800	-0.12777900
H	-0.65130000	2.56518400	2.30982300
H	4.09145200	1.76791600	-0.37970000
H	3.53877500	0.85939300	1.06945600

I11 (deprotonated)

Electronic energy (E_e)	E_e + ZPV
Hartree	
-1980.710316	-1980.220563

XYZ coordinates

N	1.25646000	2.72227000	0.47831600
C	0.84114400	4.04500700	0.83134100
N	1.26773200	5.07582600	0.07198600
C	2.14349500	4.86308700	-0.92860100
C	2.49118400	3.43231600	-1.30470500
O	0.18260700	4.16911400	1.85917700
C	0.48287000	1.60891500	0.96071800
H	0.14151500	1.88144200	1.95321000
C	-0.69713000	1.23686200	0.05234200
H	-1.06955300	2.08740000	-0.50713000
C	-0.12603700	0.12150400	-0.84622400
H	0.16527800	0.54278200	-1.80642100
C	1.06875900	-0.42770100	-0.05608400
H	0.80948700	-1.41052700	0.34321300
C	2.30205300	-0.55763100	-0.91638200
H	2.08260600	-1.17313300	-1.79043500
H	2.65671800	0.41979200	-1.24057100
O	1.28543100	0.44480100	1.04485800
O	3.30759500	-1.19766500	-0.12355800
C	4.45179600	-1.47983900	-0.74941700
O	4.64220600	-1.24457800	-1.91752500
O	-1.02623800	-0.96697200	-1.04835200
C	-2.21490500	-0.66925700	-1.59362400

O	-2.47512700	0.41584800	-2.04747500
O	1.44980700	2.55792200	-0.91359900
O	2.69619400	5.76650400	-1.55628700
C	-3.17588000	-1.80361200	-1.52565600
C	-2.79272600	-3.07014700	-1.09118100
C	-4.50811300	-1.54094300	-1.83470400
C	-3.74451600	-4.07291300	-0.97197000
H	-1.75932100	-3.26593500	-0.84329700
C	-5.45789600	-2.54197600	-1.70139700
H	-4.78869600	-0.54701700	-2.15484100
C	-5.07640400	-3.80786300	-1.26995400
H	-3.44817700	-5.05809400	-0.63912500
H	-6.49487200	-2.33388800	-1.92661300
H	-5.81852100	-4.58786100	-1.16349200
C	5.45316600	-2.12352100	0.14631700
C	6.63856600	-2.58498300	-0.42007500
C	5.22655500	-2.26749200	1.51315200
C	7.59338300	-3.19633300	0.37777200
H	6.79814800	-2.46053600	-1.48212200
C	6.18708700	-2.87547900	2.30889900
H	4.30853100	-1.89900100	1.94753800
C	7.36737500	-3.34198500	1.74225200
H	8.51268500	-3.55886400	-0.06124000
H	6.01466600	-2.98370200	3.37079100
H	8.11341500	-3.81724700	2.36496500
O	-1.74318800	0.66498000	0.83179100
C	-2.93931100	1.29041200	0.82687800
O	-3.10052100	2.41883100	0.45211000
C	-4.03273200	0.39970900	1.30639000

C	-3.79824900	-0.92754400	1.66034300
C	-5.32866800	0.90904400	1.31827900
C	-4.86205800	-1.74268100	2.02072700
H	-2.79172500	-1.31966100	1.63814400
C	-6.38862200	0.09219200	1.68080000
H	-5.49180300	1.93913800	1.03373500
C	-6.15587700	-1.23380400	2.02959600
H	-4.68169100	-2.77498900	2.28802500
H	-7.39528600	0.48654000	1.68772000
H	-6.98476700	-1.87137300	2.30683300
H	2.57809200	3.34732000	-2.38506100
H	3.43393600	3.14039300	-0.83005600

I12

Electronic energy (E_e)	E_e + ZPV
Hartree	
-1554.338102	-1553.859145

XYZ coordinates

N	2.66372700	-0.74599200	0.33756600
H	4.79858000	-2.23219100	-1.57605400
C	3.65541600	-0.85684000	-0.62067400
N	4.08638800	-2.13856400	-0.86223500
C	3.69894900	-3.30204400	-0.19910500
C	2.73017600	-3.07458700	0.85107500
C	2.27440500	-1.83080800	1.07860500
O	4.09983000	0.11067500	-1.20539300
O	4.17783900	-4.37452000	-0.51606400

C	2.22502100	0.64200900	0.65267000
H	3.11259500	1.19971300	0.94819300
C	1.54383100	1.32319800	-0.55335800
H	1.64290100	0.72677900	-1.45464300
C	0.08415500	1.54867600	-0.10827800
H	-0.64957900	1.16496700	-0.81408500
C	-0.03228400	0.83878000	1.23436100
H	-0.53647800	1.46745400	1.96690500
C	-0.77530900	-0.48326300	1.10723900
H	-0.31683200	-1.08752800	0.31262500
H	-0.69466500	-1.03928600	2.04522100
O	1.31066500	0.62445400	1.70330100
O	-0.05650100	2.95350200	-0.04639700
O	-2.12257400	-0.18730100	0.82460900
H	2.40415900	-3.91068800	1.44684700
O	2.04132500	2.61095200	-0.80904400
C	1.22383700	3.56850400	-0.14182700
C	1.11422800	4.79299200	-1.01848700
H	0.43366200	5.51282800	-0.56612700
H	2.09268900	5.25806200	-1.13196000
H	0.73641100	4.50681400	-1.99823800
C	1.76815100	3.88861600	1.24068900
H	2.77724000	4.29079500	1.15685200
H	1.13101400	4.63231600	1.71824100
H	1.79096500	3.00104800	1.87203900
Si	-3.03955600	-1.14250800	-0.19520600
C	-4.73863400	-0.33496300	-0.19877000
C	-3.07553400	-2.88784900	0.46814500
H	-3.65222300	-3.54447100	-0.18599300

H	-2.06427700	-3.29691900	0.52929200
H	-3.51395400	-2.93313300	1.46629100
C	-2.26296100	-1.14375200	-1.89617700
H	-2.84734100	-1.76370300	-2.57945600
H	-2.20503700	-0.14033000	-2.32010900
H	-1.25294800	-1.55929300	-1.87240100
C	-5.24473800	-0.18518700	1.24078900
H	-4.58206700	0.44437200	1.83646600
H	-6.23609600	0.27893700	1.23996700
H	-5.33595500	-1.15104700	1.74281300
C	-5.72078100	-1.20435200	-0.99329900
H	-6.70050300	-0.71837400	-1.03631600
H	-5.38899500	-1.36018600	-2.02260300
H	-5.86003500	-2.18390800	-0.53089700
C	-4.64524400	1.05174300	-0.84807000
H	-4.36796800	0.98669100	-1.90224200
H	-5.61503900	1.55635700	-0.79283300
H	-3.91296500	1.68601800	-0.34343900
H	1.57494900	-1.61244000	1.86993900

I12 (protonated)

Electronic energy (E_e)	E_e + ZPV
Hartree	
-1554.745128	-1554.25349

XYZ coordinates

N	2.71707000	-0.68337100	0.36178200
H	5.08062800	-1.99962600	-1.41982900

C	3.78175400	-0.70290400	-0.52791100
N	4.30823000	-1.97539700	-0.75762400
C	3.87679700	-3.09790700	-0.16553800
C	2.83082000	-3.02594900	0.75079700
C	2.29719900	-1.79208200	0.99180700
O	4.22062500	0.27977200	-1.05912300
O	4.43015300	-4.24786100	-0.43611300
C	2.18094000	0.68943400	0.68146700
H	3.04057900	1.25952600	1.03154700
C	1.54448500	1.36160500	-0.55496800
H	1.69117200	0.77194600	-1.45528600
C	0.06226800	1.55593600	-0.16925800
H	-0.63318600	1.16644300	-0.90979400
C	-0.10490200	0.82642200	1.15845800
H	-0.62476300	1.44766500	1.88542400
C	-0.84745800	-0.48972200	0.99025000
H	-0.37302100	-1.07653200	0.18885900
H	-0.78235400	-1.06521700	1.91860100
O	1.22433400	0.58725300	1.67101000
O	-0.10246700	2.95489300	-0.10194000
O	-2.18224600	-0.17763000	0.68873700
H	2.48610300	-3.91078200	1.25772600
O	2.03241000	2.65389300	-0.77601000
C	1.16868300	3.59469300	-0.13366500
C	1.07836700	4.82432700	-1.00334900
H	0.37307900	5.53197400	-0.57050000
H	2.05484800	5.30142100	-1.07510700
H	0.74103200	4.54325000	-1.99914800
C	1.64745600	3.90638100	1.27373400

H	2.65463500	4.31986900	1.23839400
H	0.98114900	4.63881300	1.72762100
H	1.65064900	3.01560000	1.90171500
Si	-3.16138500	-1.19744500	-0.20791600
C	-4.85322800	-0.38078400	-0.17517200
C	-3.16095900	-2.88882100	0.58302700
H	-3.76034300	-3.59052500	0.00014200
H	-2.14754500	-3.29370400	0.63597900
H	-3.56239600	-2.86008400	1.59718300
C	-2.46492500	-1.31815600	-1.93873600
H	-3.12313400	-1.91092000	-2.57757400
H	-2.35096600	-0.33498500	-2.39779000
H	-1.49111500	-1.81247400	-1.94119300
C	-5.26111200	-0.09521800	1.27554100
H	-4.56234500	0.58793800	1.76046000
H	-6.25270000	0.36724300	1.29873600
H	-5.31221700	-1.00874900	1.87245400
C	-5.88589100	-1.31550900	-0.81687100
H	-6.86843000	-0.83408900	-0.82649300
H	-5.63234500	-1.55909500	-1.85142000
H	-5.98360300	-2.25132900	-0.26243500
C	-4.80324900	0.93729600	-0.95819300
H	-4.58877700	0.76861100	-2.01545000
H	-5.77043300	1.44539400	-0.89502900
H	-4.04638900	1.61773600	-0.56153300
H	1.51427900	-1.64489900	1.72119200
H	5.17545300	-4.19162900	-1.05675800

I12 (deprotonated)

Electronic energy (E_e)	E_e + ZPV
Hartree	
-1553.841104	-1553.375546

XYZ coordinates

N	2.53739700	-0.91576400	0.33662700
C	3.44726800	-1.19881200	-0.71455500
N	3.72708500	-2.47511600	-1.01411100
C	3.25082400	-3.50934500	-0.26836600
C	2.45531200	-3.19638100	0.91883200
C	2.13677000	-1.91742200	1.17363300
O	3.91993300	-0.21804400	-1.30420900
O	3.49120500	-4.69149500	-0.56501600
C	2.28213800	0.50466000	0.62448900
H	3.23073900	0.98462200	0.86141000
C	1.60766000	1.22851000	-0.56178900
H	1.60897500	0.60443200	-1.44790300
C	0.20280700	1.60559800	-0.05114800
H	-0.60063100	1.31805900	-0.72638800
C	0.07829300	0.89934900	1.28971300
H	-0.38773500	1.54713700	2.03168500
C	-0.72305600	-0.39057900	1.17181300
H	-0.28783700	-1.02322200	0.38856000
H	-0.67117000	-0.94049500	2.11462500
O	1.41785000	0.63820200	1.72622700
O	0.22074700	3.01958000	0.04629500
O	-2.06321800	-0.04833400	0.88279000
H	2.13422600	-3.99358200	1.57074800

O	2.20450300	2.46340100	-0.88341100
C	1.54389400	3.49996500	-0.17504800
C	1.48043100	4.71908900	-1.06630100
H	0.93577700	5.51878700	-0.56599700
H	2.48831700	5.06725300	-1.28913300
H	0.97483100	4.46537700	-1.99616800
C	2.23141800	3.79102500	1.15028300
H	3.26447400	4.08812800	0.97191000
H	1.71379300	4.60285400	1.66130700
H	2.22062600	2.91446700	1.79640000
Si	-2.97145100	-0.96672600	-0.17449800
C	-4.65872900	-0.12876800	-0.20458800
C	-3.06373400	-2.72379400	0.45337700
H	-3.60259300	-3.36227200	-0.24913600
H	-2.06004200	-3.14133400	0.56436300
H	-3.56119000	-2.78745000	1.42234600
C	-2.16339300	-0.96173600	-1.85998900
H	-2.73912900	-1.57322400	-2.55819600
H	-2.08535000	0.04238200	-2.27900200
H	-1.15824200	-1.38766200	-1.81738800
C	-5.19702900	0.01115200	1.22395700
H	-4.53979600	0.62486000	1.84178400
H	-6.18229200	0.48855400	1.20622400
H	-5.31203300	-0.95885400	1.71291100
C	-5.63535300	-0.97192600	-1.03342200
H	-6.60749600	-0.47191300	-1.09211900
H	-5.28150800	-1.11869700	-2.05672200
H	-5.79785500	-1.95600100	-0.58831800
C	-4.52897400	1.26468900	-0.83265500

H	-4.21834100	1.20940300	-1.87800400
H	-5.49357200	1.78172400	-0.80191500
H	-3.80452900	1.88250000	-0.29734600
H	1.56108600	-1.61363500	2.03400900

I13

Electronic energy (E_e)	E_e + ZPV
Hartree	
-1782.194815	-1781.673788

XYZ coordinates

N	2.15617400	-0.72673100	-0.55277400
H	2.49260100	-2.43395100	-3.27851800
C	2.44543300	-0.95518000	-1.88953800
N	2.32833900	-2.26629100	-2.29294100
C	2.07290500	-3.37465700	-1.49432300
C	2.02362800	-3.06410500	-0.07534100
C	2.10759200	-1.78732000	0.32974900
O	2.75166900	-0.06651800	-2.65464400
O	1.96105500	-4.48032300	-1.98202000
C	2.00959400	0.69063900	-0.09897500
H	3.00274000	1.12848100	-0.02606700
C	1.09823700	1.53065700	-1.03358800
H	0.82915000	0.99839500	-1.93723500
C	-0.11330300	1.90246700	-0.16274200
H	-1.06771100	1.78141400	-0.67248700
C	-0.00343300	0.98620500	1.03798600
H	-0.31377500	1.47912100	1.95705300

C	-0.79643400	-0.29901900	0.84131200
H	-0.50609800	-0.76342200	-0.11017900
H	-0.55737800	-1.00004500	1.64402500
O	1.40247100	0.70527700	1.16038600
O	0.08306800	3.26756700	0.15114900
O	-2.16763900	0.02962800	0.85581800
H	1.99919700	-3.87476700	0.63514200
O	1.67413700	2.75916600	-1.38629900
C	1.29494800	3.74602800	-0.42919900
C	1.01961400	5.03544400	-1.16708400
H	0.66088200	5.79026600	-0.46881300
H	1.93263300	5.39790800	-1.63782100
H	0.26399700	4.86604400	-1.93235700
C	2.35443000	3.90621100	0.64759800
H	3.30532500	4.18545700	0.19517900
H	2.05056300	4.69006800	1.34076100
H	2.47877700	2.98211200	1.21056300
Si	-3.25623500	-0.80504600	-0.09939800
C	-4.92259800	-0.00633000	0.26220900
C	-3.20883800	-2.61389200	0.35870500
H	-3.86591200	-3.20422800	-0.28239200
H	-2.19752600	-3.01139500	0.24116300
H	-3.50914900	-2.77432000	1.39518800
C	-2.76674900	-0.60322300	-1.89455200
H	-3.49109800	-1.09699600	-2.54561800
H	-2.71846900	0.44702300	-2.18718100
H	-1.79496200	-1.05924800	-2.09764500
C	-5.19822600	-0.04010400	1.76994400
H	-4.43732800	0.50610000	2.32939000

H	-6.16784000	0.42147500	1.98251800
H	-5.23053600	-1.06224100	2.15343200
C	-6.02788900	-0.77422900	-0.47295700
H	-6.99771300	-0.30019800	-0.29203200
H	-5.86763700	-0.78717500	-1.55346100
H	-6.09868800	-1.80832000	-0.12877800
C	-4.90444600	1.44962300	-0.21859200
H	-4.75823000	1.51593400	-1.29868300
H	-5.85700900	1.93580000	0.01472600
H	-4.11194800	2.02324800	0.26680400
C	2.31686300	-1.54104700	1.80260100
O	1.62215500	-2.00584400	2.66233900
O	3.43608700	-0.85866700	1.99729800
C	3.73037900	-0.54750400	3.36752100
H	2.91744400	0.03631500	3.79479200
H	3.86930800	-1.46386600	3.93670100
H	4.64782400	0.03054500	3.34678900

I13 (protonated)

Electronic energy (E_e)	E_e + ZPV
Hartree	
-1782.596338	-1782.061465

XYZ coordinates

N	2.36112700	-0.52208400	-0.41866700
H	3.41987100	-1.94009400	-3.13717300
C	2.89360200	-0.58807100	-1.70092000
N	3.09461100	-1.89110700	-2.17385800

C	2.96804400	-2.99495300	-1.43529400
C	2.61498300	-2.87020100	-0.08830400
C	2.36022300	-1.61597100	0.38063400
O	3.15494400	0.36763000	-2.37457500
O	3.19754800	-4.17192300	-1.93730400
C	1.94576700	0.85359700	0.07177000
H	2.86824200	1.38927500	0.28779600
C	1.07870600	1.61624500	-0.96735600
H	0.95886600	1.06598300	-1.89339900
C	-0.25231600	1.87098800	-0.24128800
H	-1.12442100	1.66979200	-0.86133900
C	-0.21575500	0.94960300	0.96093300
H	-0.62561800	1.42547200	1.84894700
C	-0.94048800	-0.35923100	0.68925700
H	-0.55332900	-0.80008300	-0.24184500
H	-0.74792400	-1.06163300	1.50252900
O	1.18508800	0.71050600	1.22059100
O	-0.21540300	3.24299500	0.08815600
O	-2.31083300	-0.06062100	0.59154300
H	2.59949000	-3.73447700	0.55567800
O	1.58349000	2.88705700	-1.25189400
C	1.01034300	3.83118500	-0.34156600
C	0.71870300	5.09960100	-1.10610000
H	0.22994900	5.81989700	-0.45223400
H	1.64771100	5.53331300	-1.47384600
H	0.06668200	4.87743100	-1.94897100
C	1.91610000	4.06108300	0.85435600
H	2.89082300	4.41661500	0.52265100
H	1.47023200	4.81110800	1.50638200

H	2.04034000	3.14509700	1.43124800
Si	-3.36804600	-1.03018900	-0.26865100
C	-5.07010800	-0.31174300	0.07698100
C	-3.18878200	-2.78756400	0.33821500
H	-3.97527800	-3.42737000	-0.06656000
H	-2.23160500	-3.21496900	0.03049800
H	-3.24247700	-2.83309800	1.42716500
C	-2.92942300	-0.93152000	-2.08447300
H	-3.52048800	-1.64354700	-2.66379400
H	-3.11268100	0.06461200	-2.49113300
H	-1.87755000	-1.17517700	-2.25246700
C	-5.46829800	-0.60040300	1.52944900
H	-4.74315700	-0.19107500	2.23620000
H	-6.43898000	-0.14472800	1.74813700
H	-5.55730100	-1.67213300	1.71899900
C	-6.09271400	-0.95133300	-0.87060100
H	-7.09628500	-0.57905200	-0.64355200
H	-5.88095800	-0.71112500	-1.91470100
H	-6.11900100	-2.03943500	-0.77326200
C	-5.04619600	1.20460200	-0.15093500
H	-4.75507400	1.45836200	-1.17285400
H	-6.04325800	1.62304500	0.01807800
H	-4.35444900	1.70145400	0.53078800
C	2.18697000	-1.47235200	1.88213400
O	1.36082400	-2.07887500	2.49795900
O	3.14552800	-0.71135500	2.36467800
C	3.06490800	-0.45236200	3.78128400
H	2.11543700	0.03012200	4.00325900
H	3.15401300	-1.38472600	4.33271200

H	3.89591700	0.20714500	4.00336000
H	3.46962400	-4.16152800	-2.87081700

I13 (deprotonated)

Electronic energy (E_e)	E_e + ZPV
Hartree	
-1781.703663	-1781.196068

XYZ coordinates

N	2.09603600	-0.83701600	-0.60249600
C	2.31324600	-1.11648700	-1.98117500
N	1.98951300	-2.33040000	-2.45354000
C	1.61252000	-3.34120400	-1.63008800
C	1.74162900	-3.12904200	-0.18085300
C	1.98783900	-1.89097800	0.27155600
O	2.76317900	-0.19325800	-2.66572700
O	1.21336500	-4.43271200	-2.05897100
C	2.08453600	0.56319600	-0.14272200
H	3.10289300	0.94343700	-0.08060300
C	1.21171000	1.47394000	-1.04367400
H	0.92625500	0.97591500	-1.96045300
C	0.01938600	1.87931000	-0.16088900
H	-0.94372700	1.79120200	-0.66059100
C	0.11472800	0.96019100	1.04031100
H	-0.16607600	1.47105900	1.96023900
C	-0.74153500	-0.28848100	0.86834200
H	-0.50424200	-0.77370500	-0.08567300
H	-0.51511600	-0.99784000	1.66724200

O	1.50544300	0.62895700	1.14341400
O	0.25474300	3.24059900	0.15729600
O	-2.10114800	0.10036100	0.92276600
H	1.68166300	-3.96665800	0.49667000
O	1.83541600	2.69226900	-1.37349400
C	1.49099000	3.67331900	-0.40562200
C	1.27435900	4.98858800	-1.11984800
H	0.94908700	5.74734900	-0.40911000
H	2.20265600	5.31827100	-1.58497800
H	0.51269000	4.86523300	-1.88789400
C	2.54933500	3.77337000	0.68113800
H	3.51032600	4.03583500	0.23972100
H	2.26626100	4.54596900	1.39588700
H	2.64425200	2.82870000	1.21421200
Si	-3.21594700	-0.63751600	-0.07775000
C	-4.86093800	0.17813000	0.35196700
C	-3.22081100	-2.47505900	0.25176300
H	-3.90157400	-2.99602000	-0.42378500
H	-2.22265600	-2.88964300	0.08931000
H	-3.51243600	-2.70415900	1.27793000
C	-2.74444300	-0.33185700	-1.86125900
H	-3.48118000	-0.77740100	-2.53313600
H	-2.68224500	0.73249900	-2.09263800
H	-1.78099200	-0.78771400	-2.10151300
C	-5.11173700	0.08518700	1.86149200
H	-4.33260300	0.59639500	2.42888400
H	-6.07050900	0.55136200	2.11172100
H	-5.15199300	-0.95114800	2.20415900
C	-5.99562900	-0.53310200	-0.39541900

H	-6.95096700	-0.04091900	-0.18616300
H	-5.84831200	-0.51259700	-1.47782700
H	-6.08913600	-1.57700300	-0.08816600
C	-4.82380000	1.65379800	-0.06419400
H	-4.71195200	1.76566000	-1.14469900
H	-5.75619500	2.15048100	0.22393100
H	-4.00206200	2.18804400	0.41803000
C	2.31287700	-1.70908700	1.72306900
O	1.66347600	-2.16143600	2.62892500
O	3.47862000	-1.08205800	1.87537200
C	3.86099000	-0.82962800	3.23012600
H	3.10059400	-0.22883100	3.72647500
H	3.99652800	-1.76667500	3.76652400
H	4.79891800	-0.28637300	3.17630900

2.10.2 Heterodimers of I1-A5 and I2-A1

I1-A5 heterodimer

Electronic energy (E_e)	E_e + ZPV
Hartree	
-804.5205426	-804.2000390

XYZ coordinates

C	-2.05627600	1.48504500	-0.27579000
C	-1.22581600	-0.82166400	-0.45785300
C	-2.63979400	-1.33731700	-0.49512000
C	-3.63838000	-0.43776600	0.23772000
C	-3.47507800	0.97737600	-0.32304600

H	-2.91432400	-1.41091800	-1.55265500
H	-2.62889100	-2.34746800	-0.08793600
H	-3.77591500	0.99475300	-1.37573700
H	-4.09582900	1.69799600	0.20772900
H	-0.07622900	0.87898100	-0.34964000
N	-1.04996000	0.53766100	-0.38357100
O	-0.25990000	-1.56443400	-0.51046400
O	-1.77381300	2.65746600	-0.17293800
C	-3.35881400	-0.44880700	1.74329000
H	-4.06607600	0.19734800	2.26513200
H	-3.46303500	-1.45859900	2.14287300
H	-2.35135400	-0.09836800	1.97719900
C	-5.05825300	-0.93262300	-0.02151500
H	-5.18710700	-1.94689500	0.36040600
H	-5.78540100	-0.28970000	0.47735200
C	3.64185300	-1.62520100	0.00022100
C	5.00460800	-0.96871700	-0.12979300
C	5.04469400	0.29976800	0.71272600
C	4.00512600	1.28721100	0.19256400
C	2.64991500	0.67323500	-0.07192800
N	2.56497000	-0.65743800	-0.19468500
H	4.82958000	0.04967400	1.75475400
H	3.51864000	-2.41488100	-0.73962800
H	5.76805900	-1.67877300	0.18642700
H	3.84726200	2.12156400	0.87379700
H	1.62614800	-1.01716100	-0.34733600
O	1.65473800	1.39769200	-0.19977400
H	-5.28420200	-0.93891300	-1.08904200
H	3.54172400	-2.07956100	0.98943200

H	5.19514700	-0.72009600	-1.17675800
H	6.03349700	0.75610200	0.68719200
H	4.33231700	1.71432400	-0.75921800

I2-A1 heterodimer

Electronic energy (E_e)	E_e + ZPV
Hartree	
-804.513376166	-804.194751

XYZ coordinates

O	-2.63978900	1.36473500	-0.29686400
N	-2.90249600	-0.87325600	0.10734900
C	-3.34136600	0.35928300	-0.18293700
C	-3.96605700	-1.85312100	0.28651200
C	-5.15202300	-1.18821500	-0.42990200
C	-4.84895700	0.30868600	-0.32129600
H	-1.93116700	-1.03742000	0.35738000
H	-6.10452100	-1.46973400	0.01006700
H	-5.15444600	-1.48798600	-1.47706800
O	0.00881100	-1.00627900	0.70539700
N	0.15121600	1.16740200	0.00462500
C	0.67027000	-0.02266100	0.43743200
C	1.11518400	2.12956500	-0.23049400
C	2.46943500	1.51629500	0.05338800
C	2.18846300	0.08354100	0.53862700
H	-0.86293800	1.31701700	-0.12187500
H	3.05190100	1.54598000	-0.86748700
H	2.98542700	2.12959300	0.79110500

O	0.88447700	3.25274000	-0.60341200
C	2.80133600	-0.99174200	-0.37272000
H	2.38662400	-1.96128000	-0.08661800
H	2.47826800	-0.79890000	-1.40102400
C	4.32483700	-1.05864900	-0.32883700
H	4.74730300	-0.06557000	-0.50463500
H	4.65006700	-1.36549200	0.66657900
C	4.87209300	-2.03728100	-1.36220700
H	5.95929200	-2.09932100	-1.31447800
H	4.47144100	-3.03918500	-1.19831900
H	4.59739100	-1.73189300	-2.37310800
C	2.57471100	-0.11741800	2.00682700
H	2.33800900	-1.13115400	2.32983800
H	3.64114400	0.05480700	2.14444200
H	2.03574700	0.58341400	2.64649700
H	-5.17613300	0.90245500	-1.17070900
H	-5.27085100	0.75049500	0.58414100
H	-3.69000100	-2.80811700	-0.15428700
H	-4.16914300	-2.00599800	1.34840500

2.10.3 A2···$CHCl_3$ complexes

Cx-1

Electronic energy (E_e)	E_e + ZPV
Hartree	
-1741.829204	-1741.719637

XYZ coordinates

C	3.33613300	-0.72711800	0.87797700
C	1.89560900	0.23621200	-0.65918500
C	4.05476000	0.33593900	0.04746400
H	1.18360900	-0.98814900	0.80499500
N	1.95975600	-0.40336000	0.53840500
O	0.92035400	0.45737200	-1.33646400
H	4.98670800	-0.00786400	-0.38858700
H	3.52954800	-0.61162100	1.94068500
H	3.59935200	-1.73820500	0.56495900
H	4.21972100	1.24910100	0.61766800
O	3.13557300	0.63505200	-1.01868000
H	-1.28063400	0.48831100	-1.05753900
C	-1.82868300	0.11834600	-0.20392400
Cl	-1.50090700	-1.62048900	-0.06533000
Cl	-1.24362000	0.97437900	1.23080300
Cl	-3.55846900	0.40565600	-0.44687600

Cx-2

Electronic energy (E_e)	E_e + ZPV
Hartree	
-1741.831369	-1741.721785

XYZ coordinates

C	-3.36561400	0.45329500	-0.49154800
C	-1.78020300	-0.80089200	0.64246300
C	-2.18648800	1.32712800	-0.05926300
H	-3.32025300	-1.71382900	-0.33194300
N	-2.78775100	-0.86141300	-0.26363200
O	-1.20785400	-1.70673800	1.19852500
H	-2.48069700	2.23024900	0.46488300
H	-3.62124400	0.60724100	-1.53611500
H	-4.24698600	0.61826600	0.12937400
H	-1.53919600	1.57492100	-0.89995400
O	-1.44461400	0.49532600	0.84981400
H	0.84834800	-0.45737000	0.93045900
C	1.45887300	-0.05882900	0.13084400
Cl	3.07932600	-0.76302600	0.25220800
Cl	0.70024200	-0.50861300	-1.40784800
Cl	1.53423300	1.70325600	0.29487800

2.10.4 Compounds studied in CCl_4

A1

Electronic energy (E_e)	E_e + ZPV
Hartree	
-286.6105564	-286.49861

XYZ coordinates

C	-1.33056700	-0.83686600	0.00023600
C	0.90111800	-0.00389100	-0.00000100
C	0.01538600	1.22994800	0.00023900
C	-1.43056700	0.71036300	-0.00031900
H	0.26104600	1.82617600	0.87720300
H	0.26147400	1.82690400	-0.87610000
H	-1.97543900	1.05317800	0.87503800
H	-1.97445100	1.05258100	-0.87652600
H	0.49212800	-2.01825600	-0.00009300
N	0.09682100	-1.09217800	-0.00014000
O	2.11584700	-0.01874200	-0.00007200
H	-1.80103600	-1.27169800	-0.88144200
H	-1.80046900	-1.27102900	0.88255200

A1 (protonated)

Electronic energy (E_e)	E_e + ZPV
Hartree	
-287.0051477	-286.879757

XYZ coordinates

C	-1.36661700	-0.84004100	0.10543500
C	0.77672900	0.01821200	0.00286500
C	-0.08349800	1.22277800	0.13744400
C	-1.47942300	0.66471300	-0.18227300
H	0.01335000	1.57256800	1.16884300
H	0.25536100	2.02109800	-0.51850500
H	-2.25975600	1.13032400	0.40969700
H	-1.70369100	0.82093100	-1.23475000
H	0.49330800	-2.00329000	-0.05604300
N	0.08701900	-1.07472000	-0.02851500
O	2.06817500	0.11197300	-0.04527000
H	-1.66339000	-1.10561500	1.11839300
H	-1.90041600	-1.46217600	-0.60561300
H	2.52755700	-0.74055600	-0.10108400

A1 (deprotonated)

Electronic energy (E_e)	E_e + ZPV
Hartree	
-286.0651134	-285.96761

XYZ coordinates

C	-1.28368600	-0.82029700	0.11680800

C	0.86342500	-0.11227300	-0.00648800
C	0.00863400	1.16966500	0.15989100
C	-1.38983400	0.68852300	-0.20009200
H	0.07880600	1.49351300	1.20273600
H	0.39670400	1.97215500	-0.46533700
H	-2.19808500	1.18651900	0.33835700
H	-1.56381100	0.82260300	-1.26982000
N	0.11023000	-1.19457400	-0.05183800
O	2.11026900	-0.03045700	-0.06034800
H	-1.62180100	-1.00815900	1.14891200
H	-1.93681300	-1.41465300	-0.52991100

A1 homodimer

Electronic energy (E_e)	E_e + ZPV
Hartree	
-573.239106	-573.013622

XYZ coordinates

O	0.99254300	1.60181100	-0.03256400
N	1.70954600	-0.57363300	-0.03217900
C	1.88237900	0.75700800	0.00780300
C	2.93856300	-1.33322300	0.13667600
C	4.02182800	-0.29990300	-0.21360200
C	3.36820800	1.03858300	0.13833400
H	0.77606600	-0.98979700	-0.00320000
H	2.95456100	-2.19820700	-0.52261500
H	3.03619800	-1.68396600	1.16687200
H	4.22508500	-0.33985100	-1.28307100

H	4.95151100	-0.48374600	0.31808800
H	3.65887800	1.87027400	-0.49759100
H	3.55315800	1.32673700	1.17555400
O	-0.99254500	-1.60181700	0.03242800
N	-1.70954100	0.57362900	0.03214000
C	-1.88238100	-0.75701000	-0.00786300
C	-2.93856700	1.33323000	-0.13659900
C	-4.02181300	0.29990000	0.21371000
C	-3.36821900	-1.03857700	-0.13831000
H	-0.77606200	0.98979200	0.00311500
H	-2.95451700	2.19818600	0.52273000
H	-3.03627000	1.68401800	-1.16677300
H	-4.22500800	0.33981300	1.28319200
H	-4.95152600	0.48376200	-0.31792000
H	-3.65885200	-1.87028700	0.49760700
H	-3.55323500	-1.32669700	-1.17552700

A3

Electronic energy (E_e)	E_e + ZPV
Hartree	
-365.2250446	-365.054783

XYZ coordinates

C	-1.38219300	0.03188400	0.02701700
C	-0.61949100	-1.14458800	0.59900700
C	0.58559100	-1.55766900	-0.25976300
C	1.84212100	-0.71980900	-0.02954900
C	0.63172500	1.48947000	0.41778800

C	1.70733600	0.75868200	-0.38308200
H	-0.28568000	-0.91040300	1.61387900
H	0.30242500	-1.52761400	-1.31535300
H	2.12759600	-0.80244200	1.02410000
H	0.73516900	1.25214500	1.48037000
H	-1.33801600	-1.95744400	0.66278500
H	0.82056700	-2.59793700	-0.03201300
H	2.66261600	-1.14906900	-0.60730300
H	0.77280800	2.56419200	0.32188100
H	2.66316600	1.25216200	-0.19496100
H	1.48876900	0.87607400	-1.44746800
N	-0.72856200	1.21952400	-0.02281700
O	-2.52320100	-0.08193200	-0.39183100
H	-1.25441700	1.97130100	-0.44005500

A3 (protonated)

Electronic energy (E_e)	E_e + ZPV
Hartree	
-365.6244961	-365.440382

XYZ coordinates

C	1.26349800	-0.03560900	0.07988100
C	0.55703400	1.15310100	0.62072200
C	-0.63627900	1.55935600	-0.26753400
C	-1.88327500	0.70669100	-0.05419000
C	-0.67800200	-1.50113100	0.42805200
C	-1.72648200	-0.77144600	-0.40143900
H	0.20802300	0.91761000	1.62970000

H	-0.32958700	1.54359900	-1.31564600
H	-2.19310300	0.79293300	0.99101400
H	-0.76193800	-1.25478200	1.48686000
H	1.28699800	1.95493000	0.68852800
H	-0.86837100	2.59503900	-0.02575200
H	-2.69323100	1.12196900	-0.65333000
H	-0.78250800	-2.57721600	0.32591200
H	-2.67647200	-1.27672000	-0.22561300
H	-1.49212300	-0.89884700	-1.46084900
N	0.70541200	-1.20074500	-0.00118400
O	2.47993400	0.17501200	-0.34380200
H	1.22995000	-1.96876600	-0.40585700
H	2.91603200	-0.61040000	-0.70921600

A3 (deprotonated)

Electronic energy (E_e)	E_e + ZPV
Hartree	
-364.672207	-364.516189

XYZ coordinates

C	1.35756600	-0.15839600	0.00674600
C	0.62685500	1.04383000	0.64458200
C	-0.53523400	1.55104900	-0.21773600
C	-1.81646900	0.73282600	-0.06121200
C	-0.58497600	-1.47374500	0.41661200
C	-1.67251400	-0.75013900	-0.39639800
H	0.24959100	0.79308000	1.64183900
H	-0.22108100	1.55255800	-1.26588600

H	-2.15154900	0.81990200	0.97875100
H	-0.69398300	-1.16709500	1.47192400
H	1.37350100	1.82694000	0.76046700
H	-0.75877500	2.58959700	0.04288800
H	-2.60553700	1.17390700	-0.67777300
H	-0.82118700	-2.54164300	0.39972300
H	-2.63934100	-1.23642200	-0.22666200
H	-1.43127900	-0.87398000	-1.45607000
N	0.76484400	-1.33041300	-0.08617300
O	2.51179500	0.08793700	-0.42769400

A3 homodimer

Electronic energy (E_e)	E_e + ZPV
Hartree	
-730.4681104	-730.124847

XYZ coordinates

C	1.90426000	0.83427600	-0.59194900
C	3.30516600	1.39518200	-0.49640800
C	3.97667500	1.10077000	0.85436200
C	4.58492900	-0.29664700	0.96284400
C	2.83738100	-1.47606500	-0.49050000
C	3.58897400	-1.44769500	0.83885300
H	3.91881600	0.99230700	-1.30697500
H	3.25125300	1.25707800	1.65702800
H	5.34522000	-0.40675600	0.18282300
H	3.54222600	-1.34912300	-1.31724600
H	3.20953400	2.46720200	-0.64766300

H	4.76938600	1.83464900	1.00395900
H	5.10664900	-0.38107700	1.91795700
H	2.36661200	-2.44814600	-0.62346500
H	4.12998600	-2.39039900	0.94281400
H	2.85769400	-1.40598100	1.64982900
N	1.76395100	-0.49916300	-0.58913200
O	0.92811100	1.58764600	-0.64701800
H	0.80630500	-0.85381100	-0.62172100
C	-1.90428000	-0.83423000	-0.59206500
C	-3.30518700	-1.39513800	-0.49654100
C	-3.97666400	-1.10083900	0.85427000
C	-4.58490600	0.29657200	0.96289700
C	-2.83738100	1.47611200	-0.49036900
C	-3.58894300	1.44762300	0.83899800
H	-3.91885100	-0.99219200	-1.30706100
H	-3.25122600	-1.25722500	1.65690700
H	-5.34521100	0.40676100	0.18290100
H	-3.54224500	1.34925600	-1.31711100
H	-3.20956400	-2.46714500	-0.64788900
H	-4.76937800	-1.83472400	1.00382100
H	-5.10660700	0.38091200	1.91802900
H	-2.36660500	2.44820100	-0.62325600
H	-4.12994200	2.39032200	0.94306700
H	-2.85764500	1.40581800	1.64995200
N	-1.76396100	0.49920900	-0.58911700
O	-0.92813300	-1.58759900	-0.64718900
H	-0.80631200	0.85385300	-0.62165700

A5

Electronic energy (E_e)	E_e + ZPV
Hartree	
-325.9205094	-325.779034

XYZ coordinates

C	1.04119500	-1.27708000	0.13824500
C	-1.13579700	-0.01309700	-0.01955000
C	-0.37220500	1.29286300	-0.11999000
C	1.08508900	1.20199700	0.32072200
C	1.73818700	-0.01112900	-0.32953400
H	1.29759800	-1.47031100	1.18422500
H	1.36890500	-2.13590200	-0.44662900
H	-0.42825400	1.59221600	-1.17021800
H	-0.93397300	2.03186600	0.44771600
H	1.61199800	2.11845800	0.05700300
H	1.13866400	1.10202400	1.40801000
H	1.66107600	0.07004600	-1.41659300
H	2.79598500	-0.07667800	-0.07652700
H	-0.95983100	-1.99515300	0.09735500
N	-0.40633800	-1.15728900	-0.00109200
O	-2.35582700	-0.03710800	-0.00200700

A5 (protonated)

Electronic energy (E_e)	E_e + ZPV
Hartree	
-326.320572	-326.165568

XYZ coordinates

C	-1.06970500	-1.29904600	-0.12710500
C	1.01399900	0.00718600	0.00812200
C	0.29813000	1.30854000	0.06158300
C	-1.17752700	1.18193700	-0.30202300
C	-1.76879100	-0.04977400	0.37091900
H	-1.33481100	-1.52189300	-1.16027500
H	-1.30001300	-2.16652900	0.48602800
H	0.43036800	1.67856300	1.08291200
H	0.83946400	1.99822400	-0.58670400
H	-1.69925900	2.08390600	0.00759100
H	-1.28427700	1.09945000	-1.38478800
H	-1.65909400	0.02584400	1.45455300
H	-2.83046500	-0.14536900	0.15338100
H	0.95190400	-1.97832700	-0.14010600
N	0.40091900	-1.12795300	-0.08061800
O	2.31657900	0.08725300	0.06506800
H	2.77048600	-0.76927500	0.06222100

A5 (deprotonated)

Electronic energy (E_e)	E_e + ZPV
Hartree	
-325.3682475	-325.241559

XYZ coordinates

C	0.99646900	-1.26849200	0.13912400
C	-1.11516200	-0.13252400	-0.00610800
C	-0.38970900	1.23212900	-0.07770200
C	1.08550800	1.19815200	0.30115500
C	1.71380800	-0.01648700	-0.36385500
H	1.31832200	-1.43690700	1.18096800
H	1.36758000	-2.13665100	-0.41803200
H	-0.50136600	1.58517600	-1.10751000
H	-0.95611500	1.92310000	0.54683500
H	1.58918400	2.12649800	0.01884600
H	1.18671500	1.09647800	1.38683900
H	1.59466800	0.06451500	-1.44930900
H	2.78429900	-0.08778400	-0.15179600
N	-0.45229500	-1.26456000	0.06367700
O	-2.37033800	-0.04489700	-0.05103300

A5 homodimer

Electronic energy (E_e)	E_e + ZPV
Hartree	
-651.8589625	-651.57426

XYZ coordinates

C	-2.85338500	-1.50484100	-0.04116100
C	-1.90793100	0.80968200	0.01913100
C	-3.30393900	1.39624900	-0.00331100
C	-4.39305400	0.40767100	-0.40514500
C	-4.19118300	-0.90024300	0.34860700
H	-3.48707000	1.77104200	1.00723900
H	-3.27110100	2.26643700	-0.65642000
H	-5.37540600	0.83288700	-0.20192100
H	-4.34164300	0.21526000	-1.47985200
H	-4.20784700	-0.70969600	1.42458000
H	-4.98279600	-1.61507400	0.12636200
H	-0.81761100	-0.87629800	0.04989500
N	-1.77815700	-0.52358700	0.04918300
O	-0.92662200	1.55877200	0.04137900
C	2.85338400	1.50484000	-0.04114600
C	1.90793300	-0.80968300	0.01919900
C	3.30394100	-1.39624800	-0.00330500
C	4.39304900	-0.40766700	-0.40515100
C	4.19118500	0.90024400	0.34860900
H	3.48709300	-1.77105500	1.00723600
H	3.27108900	-2.26642900	-0.65642500
H	5.37540500	-0.83288100	-0.20194400
H	4.34162100	-0.21525000	-1.47985600

H	4.20786300	0.70969400	1.42458100
H	4.98279300	1.61507600	0.12635500
H	0.81761100	0.87629600	0.04993600
N	1.77815700	0.52358700	0.04922400
O	0.92662400	-1.55877500	0.04137200
H	-2.59861200	-2.34088800	0.60915700
H	-2.90877100	-1.88981200	-1.06346000
H	2.59861600	2.34088900	0.60917100
H	2.90875700	1.88980700	-1.06344700

A8

Electronic energy (E_e)	E_e + ZPV
Hartree	
-247.2710398	-247.190192

XYZ coordinates

C	-1.44686000	0.12673400	-0.00005500
C	0.64271000	-0.01668800	0.00000800
C	-0.46924900	-1.07466600	0.00020700
H	-2.05992900	0.21921900	0.89384500
H	-2.05981600	0.21889100	-0.89406800
H	-0.48577900	-1.69266100	0.89340000
H	-0.48575600	-1.69306500	-0.89270600
H	-0.15577200	1.99759100	-0.00042900
O	1.84558600	-0.02869900	0.00000300
N	-0.26817800	0.99533500	-0.00014700

A8 (protonated)

Electronic energy (E_e)	E_e + ZPV
Hartree	
-247.6564532	-247.561997

XYZ coordinates

C	-1.50497400	-0.16334400	-0.00000400
C	0.51516500	0.03827500	-0.00000800
C	-0.55801000	1.06905600	0.00001000
H	-2.09159400	-0.30332700	-0.90106200
H	-2.09159300	-0.30333200	0.90105500
H	-0.57762500	1.68390400	-0.89565800
H	-0.57760900	1.68386900	0.89570400
H	-0.10246100	-1.99105900	0.00001800
O	1.79602700	0.15002900	-0.00001700
N	-0.27249400	-0.99060800	-0.00000400
H	2.26703900	-0.69994700	0.00011600

A8 (deprotonated)

Electronic energy (E_e)	E_e + ZPV
Hartree	
-246.7323529	-246.66468

XYZ coordinates

C	1.41319700	-0.18604100	-0.00007200
C	-0.59472300	-0.06500200	-0.00014100
C	0.48215700	1.03943700	0.00020000
H	2.05266800	-0.27711300	0.88595300

H	2.05254900	-0.27684400	-0.88621000
H	0.50372900	1.66587200	0.89239900
H	0.50378700	1.66631900	-0.89168000
O	-1.83183500	0.02034800	0.00006000
N	0.24830900	-1.09591100	-0.00012300

A8 homodimer

Electronic energy (E_e)	E_e + ZPV
Hartree	
-494.5582987	-494.3938

XYZ coordinates

C	-2.98017600	1.07106600	-0.03583000
C	-1.86130900	-0.67742600	0.01682200
C	-3.36790800	-0.42948900	-0.03130800
H	-3.29552900	1.63151800	0.84137600
H	-3.23862100	1.61052000	-0.94419700
H	-3.90169100	-0.77475400	0.84969500
H	-3.84660200	-0.79537000	-0.93527400
H	-0.69137300	1.14337500	0.03223400
O	-1.15008800	-1.66351500	0.04505500
N	-1.58056100	0.64152900	0.01408000
C	2.98001100	-1.07116000	-0.03584100
C	1.86139400	0.67748700	0.01677200
C	3.36796600	0.42934400	-0.03116900
H	3.29516900	-1.63168700	0.84138600
H	3.23852400	-1.61058700	-0.94420300
H	3.90165900	0.77446400	0.84995200
H	3.84689700	0.79523400	-0.93500700

H	0.69117000	-1.14313900	0.03179200
O	1.15024800	1.66361400	0.04491400
N	1.58045300	-0.64142800	0.01389500

A9

Electronic energy (E_e)	E_e + ZPV
Hartree	
-404.521883	-404.32214

XYZ coordinates

C	-1.45819400	-0.06886600	-0.11588000
C	1.37127700	1.34970800	-0.45694900
C	0.36582400	-1.48290400	0.86443500
C	1.43104700	-0.06775900	-1.03719800
C	1.59870700	-1.20213600	-0.02297300
H	0.37680900	-0.88475300	1.77043600
H	0.38765100	-2.52103900	1.19234200
H	2.45533200	-1.00651400	0.62779500
H	1.83249900	-2.11264900	-0.57797900
C	-0.99776800	1.18929100	0.61406600
H	-1.46468000	1.15176900	1.60293700
H	-1.48849600	1.99489500	0.07093600
C	0.49277800	1.52601100	0.78182800
H	0.53683600	2.57488200	1.08159800
H	0.93098900	0.97460900	1.61112200
H	-1.25009700	-2.02331500	-0.38803300
N	-0.90433100	-1.27416000	0.19385900
O	-2.37974700	-0.00115200	-0.91382400

H	2.38287400	1.66191600	-0.18635000
H	1.03594400	2.03309000	-1.24096800
H	0.54212600	-0.26348200	-1.63866800
H	2.26848500	-0.11113200	-1.73556700

A9 (protonated)

Electronic energy (E_e)	E_e + ZPV
Hartree	
-404.9233772	-404.710271

XYZ coordinates

C	-1.41877600	0.00910200	-0.02947800
C	1.69440000	1.19495200	-0.24197900
C	0.38161100	-1.47633800	0.85999100
C	1.53851400	-0.12025300	-1.00434000
C	1.51654400	-1.39131300	-0.16035600
H	0.53285000	-0.82985300	1.71329600
H	0.28450200	-2.48990800	1.23919800
H	2.44891200	-1.48750900	0.39906000
H	1.45714500	-2.25592900	-0.82312200
C	-0.83927500	1.36221900	0.23411500
H	-1.55635900	1.82223400	0.92096900
H	-0.97202800	1.89650500	-0.71125000
C	0.58319500	1.55161700	0.75405500
H	0.66464000	2.61524100	0.97854400
H	0.71758000	1.05579100	1.71084000
H	-1.47602100	-1.96354200	-0.01960900
N	-0.92954500	-1.15551800	0.26061400

O	-2.59111000	0.09892200	-0.61195800
H	2.64596900	1.19609700	0.29247200
H	1.75764900	1.99601600	-0.98066300
H	0.64546900	-0.08037100	-1.63633700
H	2.37039600	-0.20534600	-1.70392700
H	-3.02229400	-0.75209200	-0.78015800

A9 (deprotonated)

Electronic energy (E_e)	E_e + ZPV
Hartree	
-403.9682719	-403.782855

XYZ coordinates

C	-1.46786700	-0.17816800	-0.13178100
C	1.37982800	1.33432100	-0.46184400
C	0.27577400	-1.46737000	0.83316700
C	1.47212700	-0.09209800	-1.01673400
C	1.57993400	-1.20594200	0.02651600
H	0.25802400	-0.83680000	1.72985500
H	0.34410600	-2.49247400	1.21267400
H	2.40806300	-1.00954400	0.71811100
H	1.82984900	-2.12653800	-0.50709600
C	-0.98595800	1.11066300	0.61776500
H	-1.43277600	1.06920000	1.61741500
H	-1.49863600	1.91639200	0.09340600
C	0.49426200	1.50227900	0.77427900
H	0.51906500	2.55945500	1.05910000
H	0.95956000	0.97079400	1.60334200

N	-0.91778800	-1.36176600	0.03516000
O	-2.47473900	0.02886800	-0.85702000
H	2.38449300	1.67953500	-0.19653400
H	1.02564300	1.99865100	-1.25569600
H	0.59798300	-0.30524000	-1.63059900
H	2.33845900	-0.14412300	-1.68214800

A9 homodimer

Electronic energy (E_e)	E_e + ZPV
Hartree	
-809.0615731	-808.659941

XYZ coordinates

C	1.92731800	-0.81330800	-0.71668300
C	4.68132500	-0.29430100	1.14954800
C	2.77613900	1.56820000	-0.62420300
C	3.49568200	0.54047700	1.63296900
C	3.19698800	1.80535000	0.83260500
H	3.63169600	1.36881600	-1.25943600
H	2.31834400	2.47723300	-1.01196600
H	4.06923400	2.46479100	0.83232200
H	2.39192600	2.34519800	1.33540500
C	3.27950300	-1.51758900	-0.64412100
H	3.38102700	-1.99105700	-1.62382700
H	3.10668400	-2.33805700	0.05519400
C	4.59553500	-0.82530500	-0.28648500
H	5.37611600	-1.57974200	-0.40604700
H	4.84214000	-0.05011100	-1.00670500

H	0.80728800	0.82969900	-0.80147300
N	1.78151800	0.51982000	-0.78001000
O	0.93199200	-1.54556000	-0.76498500
H	5.60153900	0.28676600	1.25224000
H	4.78133100	-1.14930900	1.82331200
H	2.59838200	-0.08265500	1.66053400
H	3.68352200	0.82954800	2.66878000
C	-1.92733600	0.81326300	-0.71675300
C	-4.68122200	0.29442600	1.14965200
C	-2.77618600	-1.56821000	-0.62418700
C	-3.49560500	-0.54042400	1.63301000
C	-3.19702400	-1.80532000	0.83263500
H	-3.63174100	-1.36880400	-1.25941700
H	-2.31842900	-2.47726700	-1.01193900
H	-4.06931700	-2.46469900	0.83237500
H	-2.39199100	-2.34522500	1.33542000
C	-3.27953400	1.51752900	-0.64426200
H	-3.38111500	1.99081000	-1.62405400
H	-3.10669500	2.33813100	0.05488900
C	-4.59554300	0.82529400	-0.28643500
H	-5.37612300	1.57973000	-0.40600500
H	-4.84222800	0.05002600	-1.00654800
H	-0.80731000	-0.82975600	-0.80145700
N	-1.78153600	-0.51986700	-0.78003100
O	-0.93201100	1.54551600	-0.76504800
H	-5.60147800	-0.28654700	1.25248900
H	-4.78107800	1.14949800	1.82335900
H	-2.59826800	0.08265400	1.66053100
H	-3.68340900	-0.82948200	2.66883200

I14

Electronic energy (E_e)	E_e + ZPV
Hartree	
-478.5775888	-478.399648

XYZ coordinates

C	0.37823300	1.18768300	-0.16209800
C	1.76021700	-0.67578700	-0.05173600
C	0.36189500	-1.19362100	0.21441700
C	-0.55700200	0.04223100	0.22684100
H	0.11209800	-1.89581000	-0.58174500
H	0.36223000	-1.74702200	1.15202000
H	2.46068600	1.26355300	-0.49536200
N	1.66172200	0.68562300	-0.27348700
O	2.78632800	-1.30242400	-0.08346000
O	0.07707800	2.33982100	-0.33385900
C	-1.09318000	0.34337600	1.62954300
H	-1.70032000	1.24833800	1.61967800
H	-1.70253100	-0.48532700	1.98766100
H	-0.27503900	0.48791400	2.33690100
C	-1.68458400	-0.03300100	-0.81335500
H	-2.15844800	0.94793600	-0.87242500
H	-1.23710500	-0.22524200	-1.79220800
C	-2.73018300	-1.09952100	-0.50886500
H	-3.43942300	-1.17947100	-1.33185200
H	-2.27618800	-2.08225700	-0.36779800
H	-3.29763500	-0.85930800	0.38961500

I14 (protonated)

Electronic energy (E_e)	E_e + ZPV
Hartree	
-478.952086345	-478.760912

XYZ coordinates

C	0.40540700	1.08748300	-0.13002100
C	1.74943700	-0.75789700	-0.04180300
C	0.35650600	-1.24461700	0.25624500
C	-0.55436000	0.00043100	0.24259500
H	0.08326600	-1.97094400	-0.50950500
H	0.36514200	-1.76068100	1.21466400
H	2.44031500	1.24793100	-0.52213200
N	1.63386000	0.67522500	-0.28369100
O	2.78942600	-1.31433200	-0.09891600
O	-0.00589900	2.29863600	-0.26604700
C	-1.12700300	0.32266200	1.63235800
H	-1.76225900	1.20648800	1.59921100
H	-1.72297300	-0.52258000	1.96937300
H	-0.33171200	0.48537800	2.36001600
C	-1.65383700	-0.06700200	-0.84231000
H	-2.12514600	0.91307900	-0.92181600
H	-1.17706600	-0.27715300	-1.80278700
C	-2.70948400	-1.12298700	-0.54210300
H	-3.39874800	-1.19769100	-1.38110300
H	-2.26786900	-2.10829300	-0.38732700
H	-3.29217900	-0.86555600	0.34069800
H	0.68400200	2.94058300	-0.50351700

I14 (deprotonated)

Electronic energy (E_e)	E_e + ZPV
Hartree	
-478.0603247	-477.896067

XYZ coordinates

C	0.43913600	1.17981500	-0.18671200
C	1.80101600	-0.55580900	-0.07788300
C	0.42223900	-1.17002400	0.20841900
C	-0.51860600	0.02994600	0.22555600
H	0.19331900	-1.88435900	-0.58512400
H	0.45854000	-1.72574600	1.14617300
N	1.72590100	0.77648800	-0.31273200
O	2.81918800	-1.24144600	-0.08975900
O	0.01451900	2.31996400	-0.34984100
C	-1.04927900	0.33836100	1.62664400
H	-1.64481100	1.25253000	1.61444500
H	-1.66509900	-0.47767200	2.00902600
H	-0.22146800	0.48572400	2.32311800
C	-1.65612600	-0.05940000	-0.79751800
H	-2.13062000	0.92161700	-0.86368600
H	-1.21618600	-0.25855300	-1.77899700
C	-2.70366400	-1.12503600	-0.48683600
H	-3.42163200	-1.21421100	-1.30344000
H	-2.24477900	-2.10558000	-0.34128900
H	-3.26653700	-0.88443100	0.41567400

I14 homodimer

Electronic energy (E_e)	E_e + ZPV
Hartree	
-957.1692518	-956.811884

XYZ coordinates

C	-2.57069400	1.99944000	-0.12589800
C	-2.02801100	-0.23996500	-0.05777300
C	-3.55018600	-0.23444800	-0.14637700
C	-3.89691200	1.26524700	-0.13076900
H	-0.56567100	1.26751300	-0.03284100
N	-1.56413000	1.04717000	-0.06186200
O	-1.32556300	-1.22890500	-0.00725000
C	2.57076100	-1.99949300	-0.12544200
C	2.02798700	0.23989100	-0.05758000
C	3.55014300	0.23440900	-0.14608900
C	3.89697000	-1.26525500	-0.12946800
H	0.56565300	-1.26762300	-0.03329000
N	1.56412400	-1.04725500	-0.06190700
O	1.32551700	1.22881500	-0.00701400
O	2.38739000	-3.18634900	-0.16047200
O	-2.38727500	3.18630200	-0.16050800
H	-4.44692800	1.56237600	0.76272000
H	-4.47594300	1.58723900	-0.99519200
H	4.44603700	-1.56180500	0.76480100
H	4.47696900	-1.58763700	-0.99308600
C	-3.93666300	-0.90499000	-1.46802100
H	-3.63765000	-1.95299300	-1.46498400
H	-5.01324100	-0.84703800	-1.62191700

H	-3.45045300	-0.41103900	-2.31078800
C	-4.10796700	-0.99527700	1.06717500
H	-3.64876300	-1.98477300	1.08936700
H	-3.78817100	-0.47686000	1.97504600
C	-5.62664000	-1.12548600	1.05879500
H	-5.97477900	-1.56139600	1.99447100
H	-6.11335600	-0.15486000	0.94511100
H	-5.96829700	-1.76970200	0.24948100
C	3.93633400	0.90377200	-1.46845000
H	3.63750700	1.95182700	-1.46618000
H	5.01284500	0.84547600	-1.62268400
H	3.44971800	0.40919600	-2.31061800
C	4.10808200	0.99626100	1.06668400
H	3.64907500	1.98586300	1.08796900
H	3.78824300	0.47874400	1.97504700
C	5.62678000	1.12615000	1.05807800
H	5.97505900	1.56311600	1.99319800
H	6.11321600	0.15524700	0.94555900
H	5.96852700	1.76928500	0.24794000

I15

Electronic energy (E_e)	E_e + ZPV
Hartree	
-1319.156376	-1319.054065

XYZ coordinates

C	-0.77892300	0.68239800	0.98995000
C	1.11747700	1.12764900	-0.27839500

C	0.73178200	-0.32029700	-0.59681500
C	-0.68534300	-0.46499400	-0.02983000
H	0.81192900	-0.54137900	-1.65418700
H	0.34299300	2.46137700	1.15587500
N	0.24654200	1.56291400	0.70194500
O	2.02049700	1.75248100	-0.75035100
O	-1.59729900	0.80294600	1.85332100
C	-1.12690800	-1.79585500	0.52069400
H	-2.16258700	-1.72234600	0.84371600
H	-1.03787800	-2.56924900	-0.24004700
H	-0.51515700	-2.06771200	1.37905200
Cl	-1.80969400	0.04352800	-1.36487600
Cl	1.92150700	-1.35634600	0.25573400

I15 (protonated)

Electronic energy (E_e)	E_e + ZPV
Hartree	
-1319.50671881	-1319.392226

XYZ coordinates

C	-1.08221800	1.05485000	0.10374900
C	1.16289000	1.24167900	-0.29434600
C	0.81762200	-0.22819500	-0.47473500
C	-0.58032100	-0.36633000	0.16963800
H	0.74119600	-0.39879200	-1.54965400
H	-0.19919200	2.92353200	-0.08486400
N	-0.11745300	1.90828700	-0.10134200
O	2.18738100	1.81624400	-0.30930300

O	-2.31700200	1.31083300	0.30246700
C	-0.56456800	-0.78177900	1.63818300
H	-1.56678300	-0.71644000	2.05501900
H	-0.21179800	-1.80773300	1.70752600
H	0.11473400	-0.13942800	2.20154200
Cl	-1.65424000	-1.41843300	-0.78071800
Cl	2.06721100	-1.28807500	0.15175700
H	-2.55994400	2.25353100	0.27191500

I15 (deprotonated)

Electronic energy (E_e)	E_e + ZPV
Hartree	
-1318.6615	-1318.572541

XYZ coordinates

C	-1.01311600	1.26751900	0.11512000
C	1.17294800	1.24701000	-0.26207100
C	0.73005200	-0.22332300	-0.46676600
C	-0.63578700	-0.24607600	0.18394800
H	0.65260200	-0.40682600	-1.53439500
N	0.09112000	2.03071000	-0.04729700
O	2.33646300	1.58949300	-0.35106600
O	-2.16004200	1.64125200	0.27278900
C	-0.67600400	-0.70230200	1.62915100
H	-1.66885100	-0.51499400	2.03371800
H	-0.44203000	-1.76191800	1.71219900
H	0.05445700	-0.13530100	2.20864500
Cl	-1.80895700	-1.24749600	-0.76826600

Cl	1.91990100	-1.41714400	0.14125600

I15 homodimer

Electronic energy (E_e)	E_e + ZPV
Hartree	
-2638.325484	-2638.119728

XYZ coordinates

C	2.91283200	1.55499700	-0.11469200
C	1.94604400	-0.54328100	-0.03186400
C	3.45615900	-0.79309100	0.08841000
C	4.04673900	0.55375500	-0.34835500
H	0.80415900	1.21089100	-0.04705600
N	1.74372500	0.80815200	-0.07156400
O	1.08736300	-1.38920900	-0.02148400
O	2.99087500	2.74202600	-0.03046300
C	-2.91283300	-1.55499400	-0.11468100
C	-1.94604300	0.54327700	-0.03178300
C	-3.45615800	0.79308400	0.08850700
C	-4.04674500	-0.55374300	-0.34828700
H	-0.80415700	-1.21089300	-0.04708100
N	-1.74372400	-0.80815400	-0.07154800
O	-1.08736200	1.38920600	-0.02140700
O	-2.99086900	-2.74203000	-0.03054100
H	-4.24093400	-0.54460800	-1.41911600
H	4.24089200	0.54465900	-1.41919100
Cl	5.55309600	1.04300900	0.44060800
Cl	3.93633200	-2.11102900	-1.02513100

Cl	-5.55307200	-1.04299900	0.44072900
Cl	-3.93635200	2.11105800	-1.02498500
C	-3.75669900	1.19596500	1.52331300
H	-3.18313900	2.08508200	1.77572200
H	-4.81769300	1.40260700	1.63787800
H	-3.48253800	0.38669400	2.20256300
C	3.75669100	-1.19603500	1.52320400
H	3.18312300	-2.08515800	1.77557500
H	4.81768200	-1.40269100	1.63776800
H	3.48253200	-0.38679200	2.20248800

I16

Electronic energy (E_e)	E_e + ZPV
Hartree	
-858.3353243	-858.24765

XYZ coordinates

C	0.34512200	1.34133200	0.00000000
C	-1.64489100	0.21323000	0.00000000
C	-0.54334500	-0.81492600	0.00000000
C	0.60343800	-0.14103000	0.00000000
H	-1.52771000	2.33944800	0.00000300
N	-1.03478400	1.45962000	0.00000100
O	-2.82565000	0.00019200	0.00000000
O	1.14646900	2.22987100	-0.00000100
Cl	2.20330900	-0.73482000	0.00000000
C	-0.83814400	-2.26542800	0.00000000
H	0.07772500	-2.85084500	0.00000000

H	-1.43120800	-2.52678600	0.87711400
H	-1.43120800	-2.52678500	-0.87711400

I16 (protonated)

Electronic energy (E_e)	E_e + ZPV
Hartree	
-858.692487938	-858.592313

XYZ coordinates

C	-0.39731800	-1.33819200	-0.00021600
C	1.53574700	-0.21122800	0.00010900
C	0.48388400	0.81872700	-0.00021300
C	-0.67070300	0.13902800	0.00007400
H	1.54815900	-2.31416700	0.00241400
N	1.04455400	-1.43281000	-0.00072500
O	2.77233800	0.12270700	0.00069200
O	-1.13279200	-2.25742300	-0.00032600
Cl	-2.25334600	0.70807800	0.00037600
C	0.77599400	2.27156200	-0.00058600
H	-0.14989100	2.83980000	0.00030800
H	1.35841500	2.54322600	0.88024800
H	1.35659900	2.54312500	-0.88265000
H	3.39973100	-0.62129200	0.00043300

I16 (deprotonated)

Electronic energy (E_e)	E_e + ZPV
Hartree	
-857.8277409	-857.753285

XYZ coordinates

C	0.30139100	1.37701100	-0.00071400
C	-1.62741200	0.34978600	-0.00036400
C	-0.58100700	-0.77239400	-0.00036500
C	0.57258700	-0.13029600	-0.00034800
N	-1.04007200	1.57473300	0.00055500
O	-2.82277900	0.09092900	0.00077200
O	1.19766100	2.20271700	0.00073300
Cl	2.17525700	-0.78174700	-0.00020200
C	-0.93035800	-2.21283400	-0.00021900
H	-0.03916000	-2.83892600	-0.00104900
H	-1.53418000	-2.45575700	0.87550700
H	-1.53578400	-2.45554700	-0.87488700

I16 homodimer

Electronic energy (E_e)	E_e + ZPV
Hartree	
-1716.683243	-1716.507499

XYZ coordinates

C	-3.03301900	1.22809900	0.01005700
C	-1.89799000	-0.74045100	0.01292800
C	-3.36263500	-1.08010900	-0.00445300

C	-4.01072400	0.08156000	-0.00609000
H	-0.88710900	1.11899000	0.02823300
N	-1.78058700	0.62923800	0.02148100
O	-0.98745400	-1.53643100	0.01817000
C	3.03270500	-1.22801500	0.00964500
C	1.89813100	0.74078000	0.01227200
C	3.36285100	1.08010500	-0.00459300
C	4.01069600	-0.08169800	-0.00608000
H	0.88683600	-1.11845500	0.02723400
N	1.78043400	-0.62891200	0.02058400
O	0.98778200	1.53697500	0.01737000
O	3.27393300	-2.39865200	0.01223300
O	-3.27445600	2.39867500	0.01264500
C	-3.83719100	-2.48247300	-0.01463500
H	-3.43809200	-3.00658700	-0.88367400
H	-3.47517500	-3.00688700	0.87047900
H	-4.92296700	-2.52744700	-0.03701400
C	3.83766300	2.48238900	-0.01461600
H	3.43951700	3.00636300	-0.88418100
H	3.47486200	3.00705800	0.87002000
H	4.92346800	2.52716900	-0.03596800
Cl	5.68940700	-0.38053100	-0.02033600
Cl	-5.68950400	0.38005200	-0.02081300

I17

Electronic energy (E_e)	E_e + ZPV
Hartree	
-757.5994171	-757.538535

XYZ coordinates

C	-1.16923300	0.93990700	0.05648900
C	1.16925200	0.93988400	-0.05648200
C	0.76631500	-0.54411700	0.09372000
C	-0.76633200	-0.54411100	-0.09373000
H	0.00003400	2.68910900	-0.00011900
N	0.00001200	1.67562600	-0.00004300
O	2.28053700	1.35242000	-0.16723700
O	-2.28050800	1.35245000	0.16733100
F	-1.07580000	-0.95110900	-1.33510500
F	-1.39784300	-1.31591700	0.78765300
F	1.07577200	-0.95115800	1.33509000
F	1.39783000	-1.31590800	-0.78767300

I17 (protonated)

Electronic energy (E_e)	E_e + ZPV
Hartree	
-757.930492896	-757.856999

XYZ coordinates

C	-1.16172000	0.80406600	0.02729700
C	1.13397100	1.01949500	-0.03564200

C	0.87999000	-0.50403300	0.06067600
C	-0.66597800	-0.64754000	-0.06436500
H	-0.26746600	2.67078300	0.06527100
N	-0.17700600	1.65379200	0.03269500
O	2.14172700	1.60639700	-0.13138700
O	-2.41131000	1.01166900	0.07044300
F	-1.01823200	-1.13305700	-1.24780100
F	-1.19670800	-1.36601000	0.90838100
F	1.28025300	-0.92804800	1.25552000
F	1.51688600	-1.14729300	-0.89954100
H	-2.69420200	1.94588100	0.11657800

I17 (deprotonated)

Electronic energy (E_e)	E_e + ZPV
Hartree	
-757.1211851	-757.072931

XYZ coordinates

C	-1.11462400	1.01005200	0.01052200
C	1.11462600	1.01004500	-0.01074400
C	0.76518300	-0.49705600	0.01517700
C	-0.76518600	-0.49705500	-0.01518700
N	0.00000400	1.77478300	-0.00006500
O	2.27679600	1.36372700	-0.03003100
O	-2.27678800	1.36373700	0.03010800
F	-1.24089400	-1.09060700	-1.13326600
F	-1.29569700	-1.15379000	1.03831000
F	1.24088800	-1.09046900	1.13333000

F	1.29569300	-1.15392500	-1.03823900

I17 homodimer

Electronic energy (E_e)	E_e + ZPV
Hartree	
-1515.210257	-1515.087036

XYZ coordinates

C	3.06800700	1.41527500	-0.04708200
C	1.91163700	-0.60541900	-0.03033500
C	3.39976000	-1.01233300	-0.00003100
C	4.16806500	0.33311800	0.04432200
H	0.94519100	1.23977200	-0.09413000
N	1.84587800	0.75877400	-0.06661400
O	0.99593800	-1.38143600	-0.02966800
C	-3.06819200	-1.41530100	-0.04683900
C	-1.91157500	0.60525200	-0.02995100
C	-3.39964900	1.01234200	0.00039000
C	-4.16811400	-0.33301800	0.04471200
H	-0.94536800	-1.24006200	-0.09395300
N	-1.84597800	-0.75894300	-0.06631700
O	-0.99578600	1.38116400	-0.02924100
O	-3.25402000	-2.58878100	-0.08471300
O	3.25369600	2.58878400	-0.08483800
F	-3.64912200	1.76643700	1.07279000
F	-3.69792600	1.70711000	-1.10223500
F	-4.84090000	-0.46693500	1.19228700
F	-5.01950600	-0.44996000	-0.97667300

F	4.84092900	0.46704700	1.19184900
F	5.01936100	0.45022300	-0.97712700
F	3.64933800	-1.76641900	1.07235300
F	3.69811600	-1.70704400	-1.10267000

I18

Electronic energy (E_e)	E_e + ZPV
Hartree	
-611.3369802	-611.106084

XYZ coordinates

O	-1.53597300	2.35527700	0.25927200
N	-2.90572600	0.54367100	0.20261100
N	-0.61321000	0.30601400	-0.12612100
C	-1.67761800	1.15919900	0.12114000
C	-0.82126300	-1.03350500	-0.31075200
C	-2.03089800	-1.61694600	-0.24313200
C	-3.19947300	-0.81149600	0.04137300
H	-3.68596400	1.15901900	0.39065000
O	-4.34231300	-1.20727500	0.14115200
H	0.06294000	-1.61447000	-0.51857900
C	0.71291200	0.94547100	-0.20974900
H	0.79722500	1.60522700	0.65247300
H	-2.15383400	-2.67628300	-0.39109500
H	0.72778200	1.57997400	-1.09682400
C	1.87904500	-0.02594100	-0.23289300
H	1.85717300	-0.63832700	-1.13831400
H	1.81229100	-0.70176700	0.62236900

C	3.20030000	0.74055200	-0.18788200
H	3.20433400	1.48962300	-0.98364900
H	3.26418100	1.28753200	0.75697100
C	4.42767500	-0.15765600	-0.33626800
H	4.39472200	-0.65421600	-1.30964100
H	5.31978200	0.47102500	-0.34038200
C	4.55608900	-1.20321400	0.76731300
H	4.52089100	-0.73465500	1.75291500
H	3.75603900	-1.94247300	0.72145200
H	5.50065400	-1.74081800	0.68793500

I18 (protonated)

Electronic energy (E_e)	E_e + ZPV
Hartree	
-611.725649329	-611.481928

XYZ coordinates

O	-1.49694700	2.36758200	0.23959500
N	-2.88028800	0.56679400	0.15642300
N	-0.56481900	0.30297000	-0.09574600
C	-1.62426400	1.18542600	0.10999000
C	-0.77069600	-1.01116700	-0.24597300
C	-2.01120600	-1.59072100	-0.20157000
C	-3.09380000	-0.74889000	0.01301100
H	-3.65004100	1.21194000	0.30995400
O	-4.30318600	-1.23979100	0.07390700
H	0.10979800	-1.61355600	-0.40878000
C	0.77822200	0.94345800	-0.13964500

H	0.84811000	1.55372000	0.75934900
H	-2.15598200	-2.65028300	-0.32275700
H	0.77125600	1.62086400	-0.99289900
C	1.93516300	-0.03097300	-0.21961800
H	1.89104300	-0.60974900	-1.14641600
H	1.88997500	-0.73184000	0.61718200
C	3.25602300	0.73949200	-0.17659200
H	3.24745800	1.50463400	-0.95628200
H	3.33145000	1.26413600	0.77968300
C	4.47936500	-0.15698700	-0.36225500
H	4.43604700	-0.62594000	-1.34851000
H	5.36865400	0.47452400	-0.36015800
C	4.62267800	-1.23019700	0.71190200
H	4.59454700	-0.79004400	1.71064200
H	3.83087000	-1.97783600	0.65202400
H	5.57076500	-1.75655800	0.60899000
H	-4.99605400	-0.58134300	0.22972100

I18 (deprotonated)

Electronic energy (E_e)	E_e + ZPV
Hartree	
-610.8125614	-610.595737

XYZ coordinates

O	-1.49378200	2.35349600	0.26960500
N	-2.98205600	0.62038100	0.25946500
N	-0.64982600	0.29641900	-0.13832900
C	-1.75661800	1.15183900	0.14396000

C	-0.86198300	-1.02894700	-0.36559700
C	-2.08971700	-1.56723400	-0.28098100
C	-3.22251700	-0.70644900	0.07327100
O	-4.35590600	-1.19431400	0.19176300
H	0.00608800	-1.62286600	-0.61125900
C	0.66188400	0.92769300	-0.25220300
H	0.75326400	1.62322100	0.58091900
H	-2.25403300	-2.61914800	-0.45440200
H	0.68874900	1.53913300	-1.15860800
C	1.83817200	-0.03679800	-0.24180200
H	1.83675500	-0.66604400	-1.13630100
H	1.75466800	-0.70238300	0.62029400
C	3.15776800	0.73012100	-0.18294900
H	3.16939300	1.47928300	-0.97917200
H	3.20928500	1.28049400	0.76106500
C	4.39316400	-0.16026000	-0.31497000
H	4.37538200	-0.65786500	-1.28858000
H	5.28481700	0.47040000	-0.30631800
C	4.50916800	-1.20753500	0.78885600
H	4.45485200	-0.74017500	1.77431000
H	3.70933000	-1.94566200	0.72902700
H	5.45621000	-1.74402300	0.72461800

I18 homodimer

Electronic energy (E_e)	E_e + ZPV
Hartree	
-1222.689185	-1222.225625

XYZ coordinates

O	-3.15651700	-2.12801600	-0.08286100
O	-0.60355400	1.61872700	0.13522600
N	-4.22761700	-0.11988300	-0.11100600
N	-1.89913900	-0.23726400	0.03210600
C	-3.09035900	-0.91880000	-0.05580200
C	-1.72670500	1.13345200	0.06056000
C	-2.95181200	1.89249700	-0.00316500
C	-4.12654100	1.23998200	-0.08537600
H	-1.04771100	-0.80987200	0.07735700
H	-5.05944200	1.77884500	-0.13479400
O	3.15654700	2.12799100	-0.08284800
N	1.89914100	0.23726900	0.03223200
N	4.22760300	0.11983400	-0.11110000
C	3.09036900	0.91877600	-0.05579700
C	4.12649400	-1.24003100	-0.08553900
C	2.95175300	-1.89251600	-0.00325600
C	1.72667100	-1.13344100	0.06062800
H	1.04773700	0.80990600	0.07748000
C	-5.50409500	-0.84958000	-0.20954800
H	-5.47966100	-1.43328500	-1.13002500
O	0.60351600	-1.61869500	0.13538200
H	5.05937400	-1.77891900	-0.13508100
C	5.50408400	0.84951800	-0.20973300

H	5.53056100	1.56295100	0.61337400
H	-2.90265200	2.96776700	0.01544800
H	2.90255800	-2.96778500	0.01530700
H	5.47964000	1.43315100	-1.13025900
H	-5.53058200	-1.56294700	0.61362000
C	-6.74111500	0.02829700	-0.17452900
H	-6.74598300	0.62872000	0.73740600
H	-6.74909200	0.71730400	-1.02364000
C	-7.99658400	-0.84152800	-0.22841500
H	-8.02424600	-1.48410000	0.65620100
H	-7.93090500	-1.50512400	-1.09398200
C	-9.29252100	-0.03609900	-0.31082900
H	-9.27718400	0.57826200	-1.21495400
H	-10.12567200	-0.73163300	-0.42517300
C	-9.54343300	0.84925400	0.90570800

3. References

1. C. S. C. Kumar, W. S. Loh, S. Chandraju, Y. F. Win, W. K. Tan, C. K. Quah and H. K. Fun, *PLoS One*, 2015, **10**, 1–23.
2. M. R. Willcott, *J. Am. Chem. Soc.*, 2009, **131**, 13180–13180.
3. www.supramolecular.org (accessed from October 2016 to January 2018)
4. C. Frassineti, S. Ghelli, P. Gans, A. Sabatini, M. S. Moruzzi and A. Vacca, *Anal. Biochem*., 1995, **231**, 374–382.
5. Y. Zhao and D. G. Truhlar, *Theor. Chem. Acc.*, 2008, **120**, 215–241.
6. M. J. Frisch, G. W. Trucks, H. B. Schlegel, G. E. Scuseria, M. A. Robb, *et al*. Gaussian 09; Gaussian, Inc., Wallingford, CT, **2013**.
7. Y. Zhao and D. G. Truhlar, *Acc. Chem. Res.*, 2008, **41**, 157–167.
8. A. V. Marenich, C. J. Cramer and D. G. Truhlar, *J. Phys. Chem. B*, 2009, **113**, 6378–6396.
9. G. G. Hammes and P. J. Lillford, *J. Am. Chem. Soc.*, 1970, **92**, 7578–7585.
10. P. Pandey, A. K. Samanta, B. Bandyopadhyay and T. Chakraborty, *Vib. Spectrosc.*, 2011, **55**, 126–131.
11. P. Pandey and T. Chakraborty, *J. Phys. Chem. A*, 2012, **116**, 8972–8979.
12. E. C. Aguiar, J. B. P. da Silva and M. N. Ramos, *Spectrochim. Acta - Part A Mol. Biomol. Spectrosc.*, 2008, **71**, 5–9.
13. O. Lukin and J. Leszczynski, *J. Phys. Chem. A*, 2002, **106**, 6775–6782.
14. M. A. Blanco, A. Martín Pendás and E. Francisco, *J. Chem. Theory Comput.*, 2005, **1**, 1096–1109.
15. Keith, T. A. AIMAll (Version 16.10.31); TK Gristmill Software, Overland Parks KS, USA, **2016** (aim.tkgristmill.com).
16. J. M. Guevara-Vela, E. Romero-Montalvo, V. A. Mora Gomez, R. Chavez-Calvillo, M. Garcia-Revilla, E. Francisco, A. M. Pendas and T. Rocha-Rinza, *Phys. Chem. Chem. Phys.*, 2016, **18**, 19557–19566.
17. E. Espinosa, E. Molins and C. Lecomte, *Chem. Phys. Lett.*, 1998, **285**, 170–173.

18. M. A. Blanco, A. Martín Pendás and E. Francisco, *J. Chem. Theory Comput.*, 2005, **1**, 1096–1109.

19. J. M. Guevara-Vela, R. Chávez-Calvillo, M. García-Revilla, J. Hernández-Trujillo, O. Christiansen, E. Francisco, Á. Martín Pendás and T. Rocha-Rinza, *Chem. - A Eur. J.*, 2013, **19**, 14304–14315.

20. E. Francisco, J. L. Casals-Sainz, T. Rocha-Rinza and A. Martín Pendás, *Theor. Chem. Acc.*, 2016, **135**, 170.

21. (a) J. M. Guevara-Vela, E. Romero-Montalvo, V. A. Mora Gómez, R. Chávez-Calvillo, M. García-Revilla, E. Francisco, Á. M. Pendás and T. Rocha-Rinza, *Phys. Chem. Chem. Phys.*, 2016, **18**, 19557–19566. (b) E. Romero-Montalvo, J. M. Guevara-Vela, W. E. Vallejo Narváez, A. Costales, Á. M. Pendás, M. Hernández-Rodríguez and T. Rocha-Rinza, *Chem. Commun.*, 2017, **53**, 3516-35129. (c) E. Romero-Montalvo, J. M. Guevara-Vela, A. Costales, Á. M. Pendás and T. Rocha-Rinza, *Phys. Chem. Chem. Phys*., 2017, **19**, 97–107.

22. F. G. Bordwell and H. E. Fried, *J. Org. Chem.*, 1991, **56**, 4218–4223.

23. F. G. Bordwell, J. E. Bartmess and J. A. Hautala, *J. Org. Chem.*, 1978, **43**, 3095–3101.

24. F. G. Bordwell, *Acc. Chem. Res.*, 1988, **21**, 456–463.

25. F. G. Bordwell and G. Z. Ji, *J. Am. Chem. Soc.*, 1991, **113**, 8398–8401.

26. G. Jakab, C. Tancon, Z. Zhang, K. M. Lippert and P. R. Schreiner, *Org. Lett.*, 2012, **14**, 1724–1727.

27. F. G. Bordwell and D. Algrim, *J. Org. Chem.*, 1976, **41**, 2507–2508.

28. X. Ni, X. Li, Z. Wang and J. P. Cheng, *Org. Lett.*, 2014, **16**, 1786–1789.

29. R. Cox and L. Druet, *Can. J. Chem.*, 1981, **59**, 1568–1573.

30. A. Bagno, G. Lovato and G. Scorrano, *J. Chem. Soc. Perkin Trans. 2*, 1993, 1091–1098.

31. M. Liler, *J. Chem. Soc. B Phys. Org.*, 1969, 385.

32. G. Stojkovic and E. Popovski, *J. Serbian Chem. Soc.*, 2006, **71**, 1061–1071.

33. D. W. Farlow and R. B. Moodie, *J. Chem. Soc. B Phys. Org.*, 1970, 334–336.

34. B. García, R. M. Casado, J. Castillo, S. Ibeas, I. Domingo and J. M. Leal, *J. Phys. Org. Chem.*, 1993, **6**, 101–106.

35. N. A. Prokopenko, I. A. Bethea, C. J. Clemens, 4th, A. Klimek, K. Wargo, C. Spivey, K. Waziri and A. Grushow, *Phys. Chem. Chem. Phys.*, 2002, **4**, 490–495.
36. J. Hine, S. Hahn and J. Hwang, *J. Org. Chem.*, 1988, **53**, 884–887.
